# Metabolic labeling with stable isotope nitrogen (^15^N) to follow amino acid and protein turnover of three plastid proteins in *Chlamydomonas reinhardtii*

**DOI:** 10.1186/1477-5956-12-14

**Published:** 2014-03-03

**Authors:** Marie-Laure A Sauer, Bing Xu, Fedora Sutton

**Affiliations:** 1 Bayer CropScience, 3500 Paramount Parkway, Morrisville, NC 27560, USA; 2 Plant Science Department, South Dakota State University, Brookings, SD 57007, USA

**Keywords:** Metabolic labeling, Stable isotopes, Protein turnover, *Chlamydomonas reinhardtii*

## Abstract

**Background:**

The length of time that a protein remains available to perform its function is significantly influenced by its turnover rate. Knowing the turnover rate of proteins involved in different processes is important to determining how long a function might progress even when the stimulus has been removed and no further synthesis of the particular proteins occurs. In this article, we describe the use of ^15^N-metabolic labeling coupled to GC-MS to follow the turnover of free amino acids and LC-MS/MS to identify and LC-MS to follow the turnover of specific proteins in *Chlamydomonas reinhardtii*.

**Results:**

To achieve the metabolic labeling, the growth medium was formulated with standard Tris acetate phosphate medium (TAP) in which^14^NH_4_Cl was replaced with ^15^NH_4_^15^NO_3_ and (^14^NH_4_)_6_Mo_7_O_24_.4H_2_O was replaced with Na_2_MoO_4_.2H_2_O. This medium designated ^15^N-TAP allowed CC-125 algal cells to grow normally. Mass isotopic distribution revealed successful ^15^N incorporation into 13 amino acids with approximately 98% labeling efficiency. Tryptic digestion of the 55 kDa SDS-PAGE bands from ^14^N- and ^15^N-labeled crude algal protein extracts followed by LC-MS/MS resulted in the identification of 27 proteins. Of these, five displayed peptide sequence confidence levels greater than 95% and protein sequence coverage greater than 25%. These proteins were the RuBisCo large subunit, ATP synthase CF_1_ alpha and beta subunits, the mitochondrial protein (F_1_F_0_ ATP synthase) and the cytosolic protein (S-adenosyl homocysteine hydroxylase). These proteins were present in both labeled and unlabeled samples. Once the newly synthesized ^15^N-labeled free amino acids and proteins obtained maximum incorporation of the ^15^N-label, turnover rates were determined after transfer of cells into ^14^N-TAP medium. The t_½_ values were determined for the three plastid proteins (RuBisCo, ATP synthase CF1 alpha and beta) by following the reduction of the ^15^N-fractional abundance over time.

**Conclusion:**

We describe a more rapid and non-radioactive method to measure free amino acid and protein turnover. Our approach is applicable for determination of protein turnover for various proteins, which will lead to a better understanding of the relationship between protein lifetime and functionality.

## Background

There are various methods used to quantify proteins from different plant species [1] including the single cellular green alga *Chlamydomonas reinhardtii*[2,3]. ^15^NH_4_Cl (98% ^15^N) has been used as the label source coupled to blue-native PAGE, to examine dimerization of the chloroplast CF_o_F_1_ ATP synthase [4]. SDS PAGE coupled to MS, MALDI-TOF and MALDI-TOF-TOF have been used to describe induction of the light harvesting polypeptide LHCBM9 as a result of sulfur starvation and photobiological hydrogen production [5].

The regulation of protein synthesis and degradation (protein turnover) is central to understanding how protein abundance changes in different biological processes [6]. Protein turnover has been measured in *Chlamydomonas reinhardtii* and other algal systems for several individual proteins: the D1 protein of the PSII reaction center [7-9]; RuBisCo large subunit [10] and the flagella [11]. Those studies utilized techniques that included assessment of protein synthesis and/or degradation by immunodetection [8-10,12] or radiolabeling (^35^S [7,11], [^14^C]acetate [8]) followed by dilution of label by growth in non-radioactive media. The proteins and their specific radioactivity were then measured over time by various techniques to derive turnover information.

Stable isotope labeling by amino acids in cell culture (SILAC) procedures with l-[^13^C_6_, ^15^N_4_]arginine [13] has also been used to study the major light harvesting complex II in *Chlamydomonas reinhardtii*. SILAC has some disadvantages, which include only partial labeling of peptides as well as the expense of the labeled amino acids.

In this article, we describe an inexpensive, rapid and non-radioactive method to measure amino acid and protein turnover in *Chlamydomonas reinhardtii*. Utilizing crude protein extracts resolved by SDS-PAGE, the 55 kDa band was excised, and we were able to follow the turnover rates of the co-resolving plastid proteins; ATP synthase CF1 α and β subunit and the RuBisCo large subunit.

## Results and discussion

### Effect on growth in ^15^N-labeled medium

**Figure 1:**
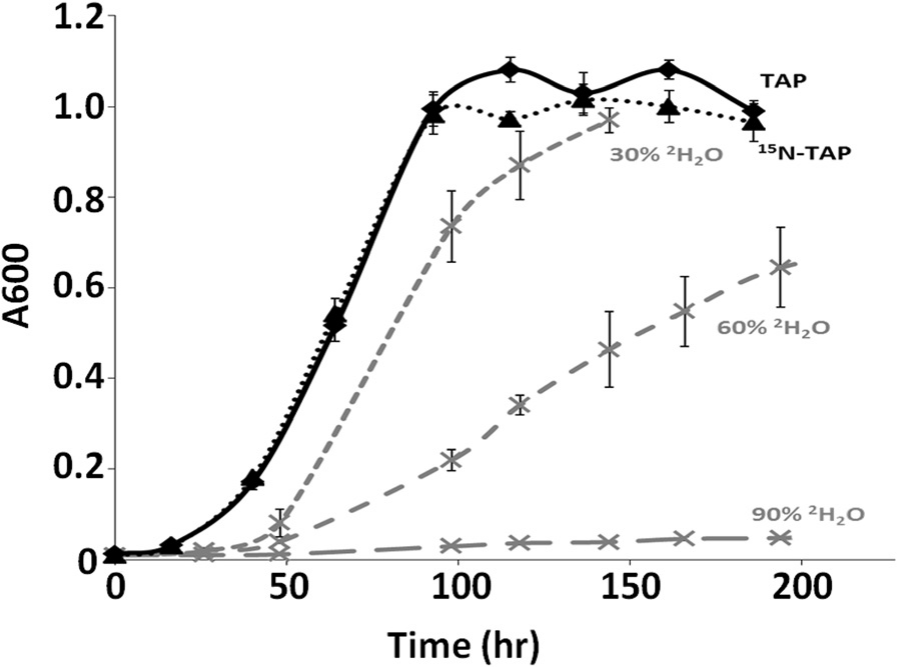
***Chlamydomonas reinhardtii *growth is normal in TAP and **^**15**^ **N-TAP but impaired in media containing D**_**2**_**O.** Algal cell cultures were grown in each medium TAP, ^15^N-TAP and TAP containing each of the following: 30%, 60% and 90% D_2_O. Log phase algae grown in TAP (100 mL) were used to inoculate 6 mL of fresh media (TAP or labeled media) in 10 mL glass tubes. The tubes were shaken horizontally under fluorescent light (16:8) light:dark photoperiod) for synchronization of cell growth, and the OD600 determined twice daily by directly placing the tubes in a Spectronic 20 (Milton Roy Company) spectrophotometer. Each data point represents the mean of five replicates. The bars indicate the coefficient of variation.

Growth curves are excellent indicators of the response of cells to stressful conditions. Therefore, growth curves were obtained for cells cultured in (i) standard tris-acetate-phosphate medium (TAP), (ii) ^15^N-TAP in which all ^14^N-labeled components were replaced with ^15^N-labeled components and (iii) media prepared with different percentages of deuterium (^2^H_2_O). The curves are depicted in Figure 1. Cells grew with the same doubling time in ^15^N-TAP as in ^14^N-TAP as reflected by the superimposition of the growth curves obtained with both media. In both cases, the curves did not display the significant lag-phase in growth as was observed in media containing ^2^H_2_O. Since cells grown in ^15^N-TAP medium in which NH_4_Cl was replaced with ^15^NH_4_^15^NO_3_ and (NH_4_)_6_Mo_7_O_24_.4H_2_O with Na_2_MoO_4_.2H_2_O allowed normal growth of the algae, we concluded that the changes made to the composition of ^14^N-TAP medium to generate ^15^N-TAP did not have an adverse impact on the cells. If cells grown in ^15^N-TAP were experiencing stress, the growth curve would have resembled that of cells grown in media containing different percentages of ^2^H_2_O (Figure 1).

Even though the strain used in this study, CC-125, carries the nit1 and nit2 mutations and cannot use nitrate as a N-source, no adverse effects were observed as depicted by the superimposed normal growth curves (Figure 1; TAP and ^15^N-TAP). Therefore, ^15^NH_4_^15^NO_3_ was a suitable replacement for ^14^NH_4_Cl and this ^15^N-TAP medium should allow healthy growth of other algal strains for similar studies.

Alternating light and dark periods are physiological conditions that result in synchronization of the cell cycle and production of homogeneous populations of cells [14]. The algal cells, cultured under the 16:8 light: dark cycle, were not negatively impacted in either growth or isotope incorporation.

### Incorporation of ^15^N in amino acids

**Figure 2:**
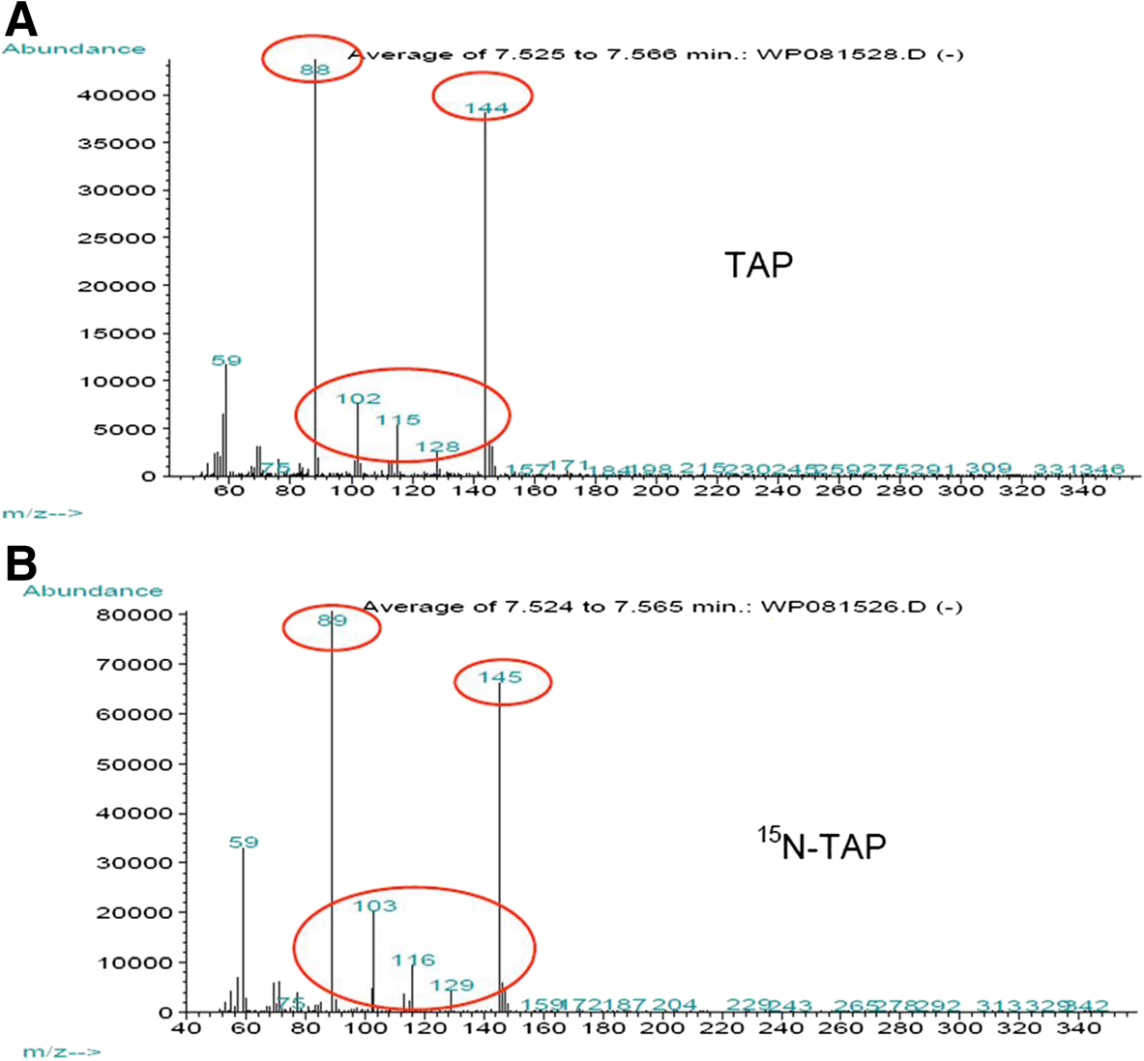
**Spectrum of Leu when grown in TAP (A) or in **^**15**^ **N-TAP (B).** Free amino acids isolated from algae subcultured three times in fresh TAP or in fresh ^15^N-TAP medium were analyzed by GC-MS. **(A)** The major fragment ions for ^14^N-Leu circled in red are 144,115,102 and 88 (m/z). **(B)** The spectrum for ^15^N-Leu, from algae grown in ^15^N-TAP, display a shift of each fragment ion peak by +1 corresponding to the replacement of ^14^N to ^15^N; peaks 145, 116, 103 and 89; circled in red on the figure.

Having determined that ^15^N-metabolic labeling does not perturb algal growth, we examined ^15^N-incorporation into free amino acids by GC-MS. The mass isotopomer distribution (MID) of N-methoxycarbonyl Leu methyl ester obtained from algae cultured in (A) TAP and (B) ^15^N-TAP are displayed in Figure 2. The unlabeled Leu-mass fragment ions were 144, 115,102 and 88 (m/z) (Figure 2A). The ^15^N- labeled ions were shifted by an m/z of 1 (145, 116, 103 and 89) (Figure 2B). Similar results were obtained for other amino acids and reflected success in metabolic labeling of the amino acids using ^15^N-TAP. The amino acids detected in our samples showed between 93.4% and 99% labeling after three subcultures of the algae in ^15^N-TAP.

### Free amino acid turnover

**Figure 3:**
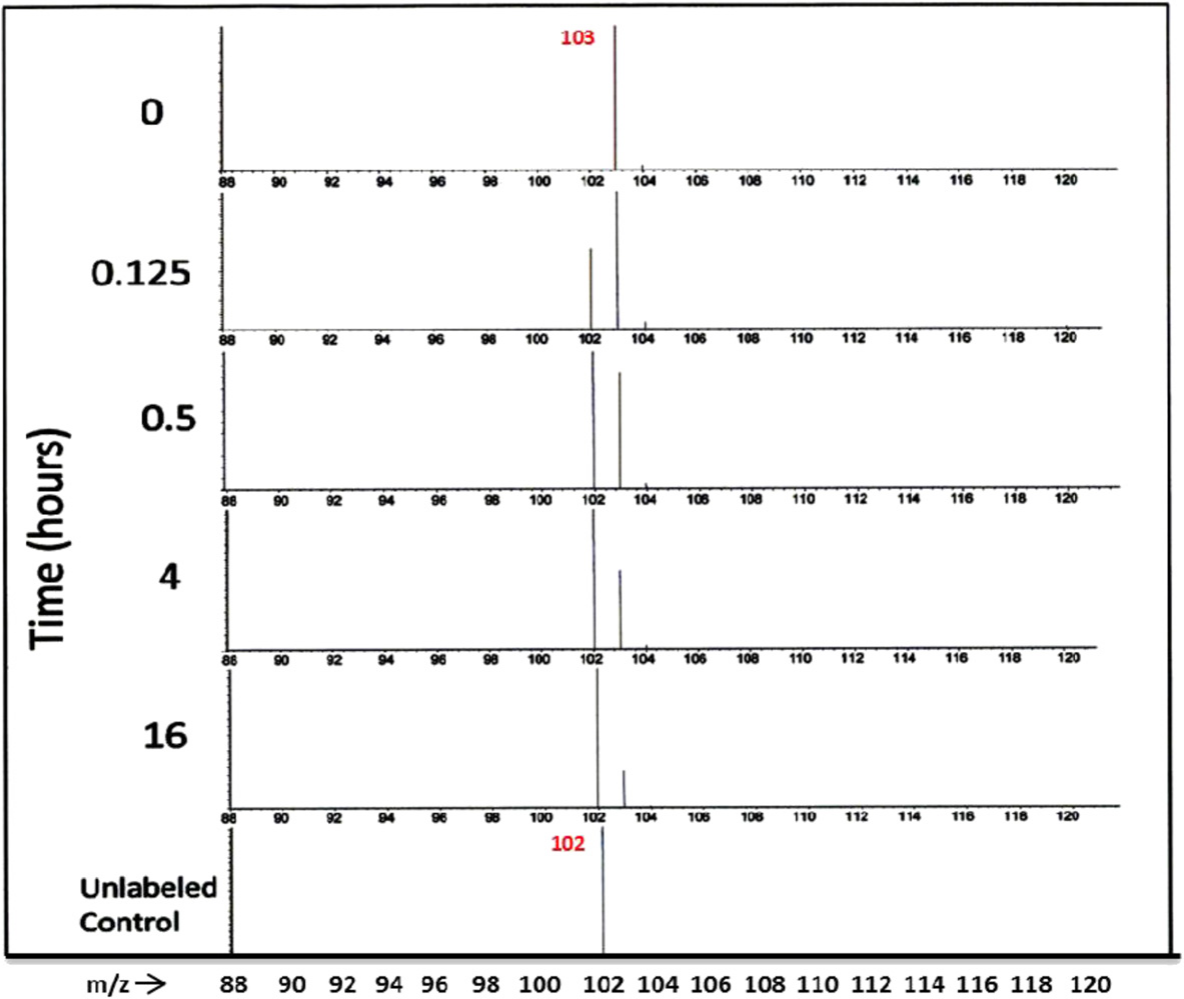
**Mass spectra of a major fragment ion of derivatized Ala at different time points of the chase experiment.** Cells were initially grown in TAP medium made with 99 atom % [^15^N] salts as the only source of nitrogen. The cells were collected by centrifugation and resuspended in fresh ^14^N- TAP medium. Aliquots (1.5 ml) of the cultures were removed for each of the time points. The cells were centrifuged at 14,000 g for 30 s. The medium discarded and the cell pellet quickly frozen in liquid nitrogen and stored at −80°C until sampling for amino acid analysis using GC-MS. The top panel depicts the ^15^N-labeled fragment ion of derivatized Ala (*m/z* 103). The bottom panel depicts the natural abundance spectrum of unlabeled Ala control (*m/z* 102). At different time points, the mass spectrum depicts the degradation of the ^15^N-labeled fragment (*m/z* 103) and emergence of the lighter fragment (*m/z* 102) during the isotopic dilution period.

GC-MS data obtained for free amino acids isolated over time after transfer from ^15^N-TAP to ^14^N-TAP displayed loss of the ^15^N-label from the amino acid major fragment ions and appearance of the newly synthesized ^14^N-labeled fragment ion. An example of such an amino acid turnover is depicted in Figure 3. The dominant Ala ^15^N-labeled fragment (*m/z =* 103) at time 0 (the uppermost panel) is displayed. Since Ala has one N, 100% of the ^15^N-label is present in the dominant Ala fragment (*m/z =* 103). By 0.125 hr, the natural abundance fragment (*m/z =* 102) noticeably increased in abundance relative to the ^15^N- *m/z* 103 fragment. By 16 hr, the majority of the ^15^N-labeled derivatized Ala fragment ion has disappeared and has been replaced with the natural abundance fragment (*m/z =* 102).

### Peptides from tryptic digest of proteins co-resolved in the 55 kDa SDS_PAGE band

With the amino acids labeled, we proceeded to examine the incorporation of ^15^N into proteins. The resulting datasets were from the Protein Pilot program. All identified peptides from the tryptic digestions of the (55 kDa) bands from crude protein extracts of ^15^N-labeled and unlabeled algae resolved by 7.5% SDS PAGE followed by LC-MS/MS are listed. The peptides for each of the proteins identified from the search of the *Chlamydomonas* database along with accession numbers are presented in order of confidence levels largest to smallest (Additional file 1).

Only proteins (20) with a minimum of two unique peptides with percent confidence levels of 95 and greater are presented (Additional file 2). Unique peptides for each protein compared with the total amino acid sequence length revealed the percent coverage for each protein. Further sorting of the peptide datasets based on proteins with unique peptides representing greater than 25% coverage of the protein limited the study to a total of five of the 20 proteins (Additional file 3: Table S1). These were: the chloroplast ATP synthase CF_1_ α and β subunits, RuBisCo, the mitochondrial F_1_F_0_ ATP synthase and cytosolic S-adenosyl homocysteine hydroxylase. The accession numbers and percent coverage of these proteins are listed in Table 1.

**Table 1:** ***C. reinhardtii *** **protein coverage**

**Protein**	**Accession #**	**Protein length**	**MW (kDa)**	**# aa from unique peptides**	**% coverage**
**RuBisCo**	gi|41179049	475	52.5	142	29.9
**ATP synthase CF1 α subunit**	gi|41179050	508	54.8	219	43.1
**ATP synthase CF1 β subunit**	gi|41179057	491	53.2	199	40.5
**Mt F1F0 ATP synthase, α subunit**	gi|159483185	569	61.5	170	29.9
**S-Adenosyl homocysteine hydrolase**	gi|159470383	483	52.7	144	29.8

Five proteins identified as co-resolving by SDS-PAGE at about 55 kDa. The band was excised from SDS-PAGE gels, the proteins extracted, tryptic digested and the samples analyzed by LC-MS/MS. Numbers of peptides were identified by Protein Pilot for each protein (Additional files 1 and 2).

**Figure 4:**
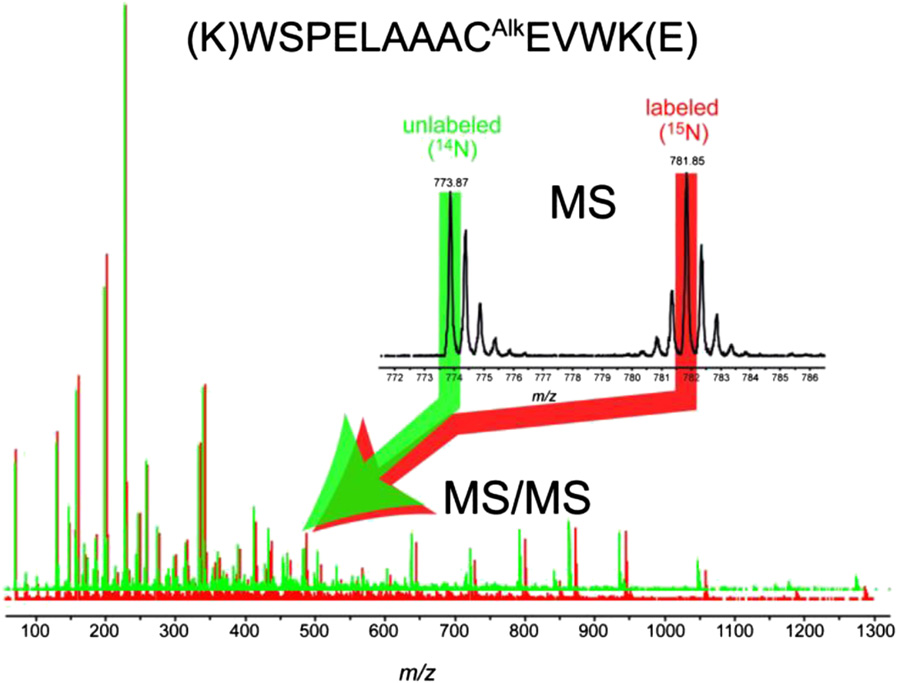
**MS and MS/MS spectrum for the RuBisCo peptide (K)WSPELAAC**^**Alk **^**EVWK(E).** The MS/MS ^15^N- and ^14^N- fragmentation patterns for the RuBusCo peptide are overlaid and slightly vertically offset for ease of comparison. The inset shows the natural abundance peptide with its monoisotopic peak highlighted in green and the ~98% atom enriched ^15^N labeled peptide with its monoisotopic peak (all ^15^N) highlighted in red.

Using RuBisCo as the model protein, the MS and MS/MS data for the RuBisCo peptide, (K)WSPELAAC^Alk^EVWK(E) is presented (Figure 4). The inset represents the isotopic distribution of the unlabeled peptide (monoisotopic peak highlighted in green) and its corresponding ^15^N-labeled peptide ~98% atom enriched (monoisotopic peak highlighted in red). Labeled and unlabeled fragmentation patterns for each one of the monoisotopic peaks are overlaid and slightly vertically offset for ease of comparison. Examination of the various peptides revealed a high incorporation of ^15^N.

### Protein turnover

**Figure 5:**
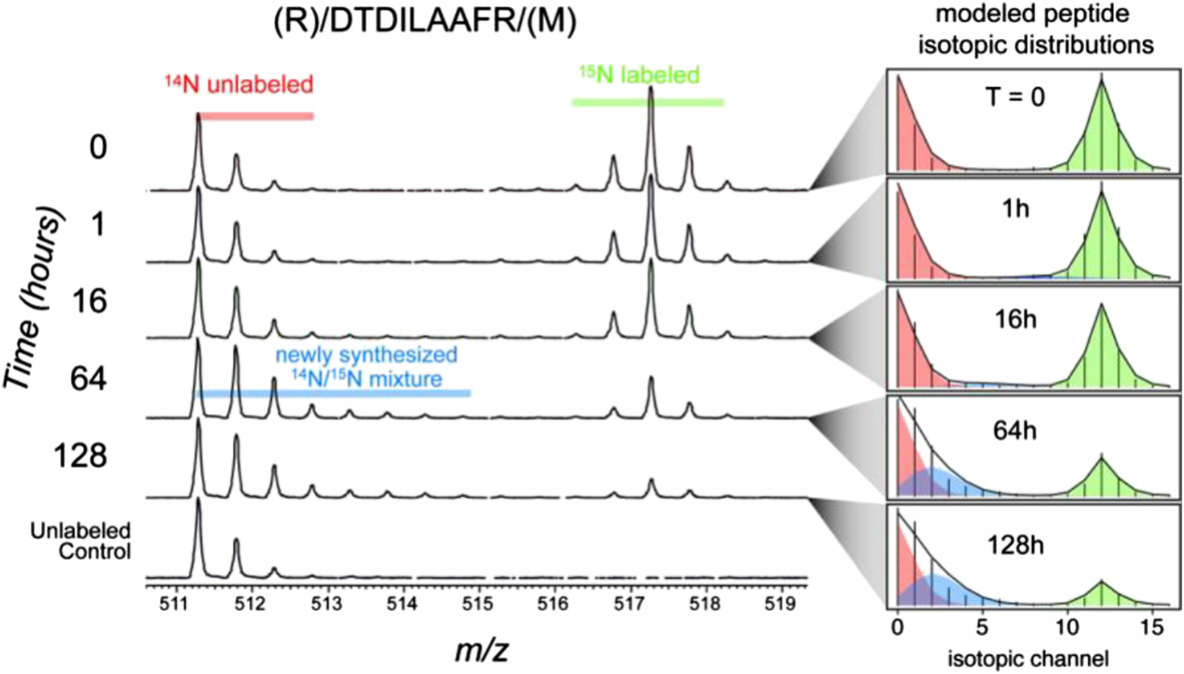
**RuBisCo turnover example peptide (R)/DTDILAAFR/(M).** Mass spectra showing degradation of old ^15^N labeled peptide (green) and emergence of newly synthesized partially labeled peptide (blue). Equal quantities of unlabeled peptide (red) were spiked into each sample to increase the success rate of peptide identification. The spike was 1:1 (unlabeled: time point) based on total protein concentration (Bradford assay). The old versus new peptide distributions are binomial or beta binomial distributions (old and new respectively) that are fitted to the data using maximum likelihood estimation. The ratio of the areas under the distributions provides relative quantities.

An example of data obtained for RuBisCo protein turnover is presented in Figure 5. The overlapping isotope distributions of ^14^N-unlabeled, ^15^N- labeled and newly synthesized ^14^N/^15^N mixture for the RuBisCo peptide, (R)/DTDILAAFR/(M) over time from 0 to 128 hr is presented. As observed, the degree of ^15^N- enrichment decreased with time and a corresponding envelope of newly synthesized, lower abundance, partially labeled ^14^N- peptide appeared (blue). By 128 hr the starting ^15^N-labeled peptide isotope distribution approached background levels.

The data for the protein turnover were obtained from MS analyses. Compared with the MS-MS datasets, there were fewer peptides identified for each of the five proteins at various time points in the experiments (Additional file 4). As a result, we focused on the three plastid proteins for which there were at least seven peptides for each protein throughout the turnover analysis (Additional file 5). It was of interest to determine whether the two ATP synthase CF1 α and β subunits share similar protein turnover profiles with each other or with RuBisCo. The calculated fractional abundance {*R*_*t*_ = ^15^*N*/(^15^*N* + ^14^*N*} for each peptide for each protein with time are presented (Additional files 4 and 5).

**Figure 6:**
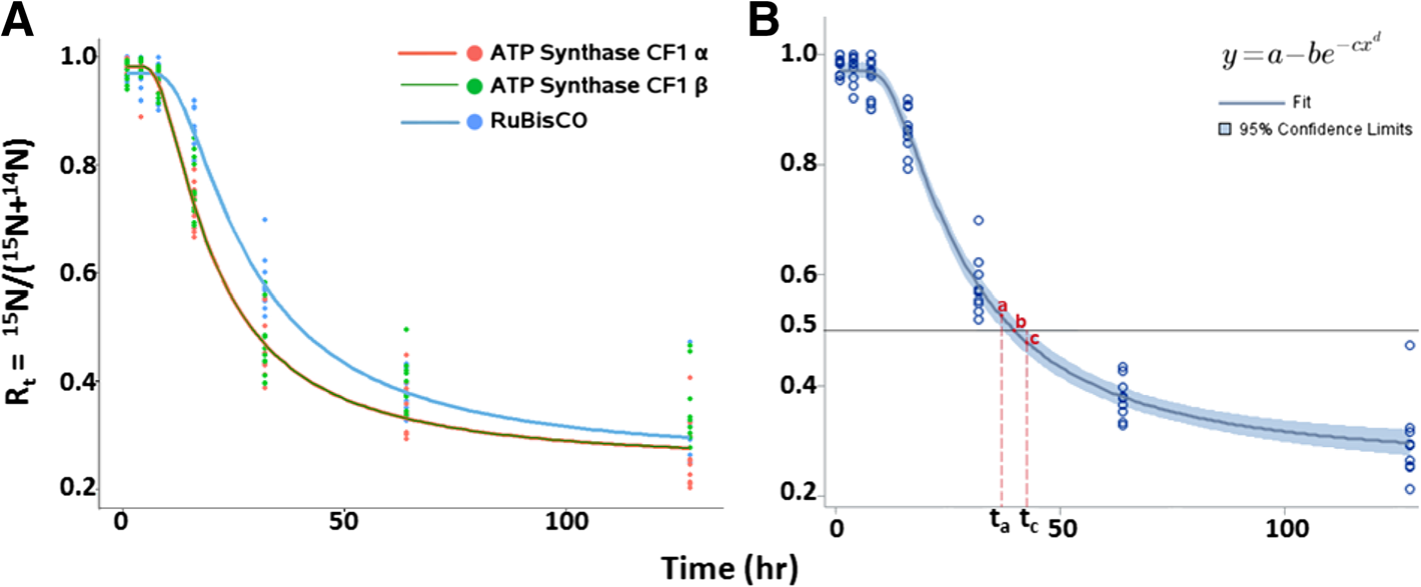
**Non-linear curve fitting of Rt (**^**15**^ **N/(**^**15**^ **N +** ^**14**^ **N)) against time (t) and estimation of t**_**1/2**_**. A**: Algal cells were collected at 1, 4, 8, 16, 32, 64, and128 hr after transfer from ^15^N-medium to ^14^N-medium. Data points for several peptides for each protein at each time point are presented. The non-linear regression model was generated with the program CurveExpert. The Parameters were estimated with SAS. The data points for all three proteins were best fitted by the Weibull model, equation {*y* = *a* − *β* * *e*^− *γ* * *xθ*^}. **B**: Three points are marked on the regression curve (a, b, and c). With respect to time, the point (a) designates the lower 95% confidence limit, and the point (c) designates the upper 95% confidence limit, of R_t_ equals to 50%. The 95% confidence interval of time when R_t_ equals 50% is estimated as: t_a_ ≤ t_1/2_ ≤ t_**c**_. This figure presented the RuBisCO data only. Similar analyses were performed for ATP synthase CF1 α and β subunits. Results for all three proteins are presented in Table 2.

The graphs of the non-linear curve fitting of the plots of values for R_t_ against time (t) are presented for all three proteins (Figure 6A). The regression curves for ATP synthase CF1 α and β subunits were superimposed indicating that the turnover rates for these proteins are identical (Table 2). The data points were best fitted by the Weibull model, equation {*y* = *a* − *β* * *e*^− *γ* * *xθ*^}.

**Table 2:** **Curve fit and 95% confidence limits of t**_**1/2**_

	**t**_**a**_	**t**_**b**_	**t**_**c**_	**Function**
**RuBisCO**	37	39	42	$0.9689-0.7163*e^{-177.7*\mathrm{tim}e^{-1.6389}}$
**ATP CF1 Alpha**	27	29	31	$0.9820-0.7355*e^{-80.4803*\mathrm{tim}e^{-1.5597}}$
**ATP CF1 Beta**	27	29	31	$0.9820-0.7355*e^{-80.4788*\mathrm{tim}e^{-1.5597}}$

The parameters for the proposed models for each protein are listed in Table 2. The 95% confidence band for the RuBisCo data is shown (Figure 6B) and allows for the determination of the t _½_ range as depicted by points (a) and (c). Similar graphing was performed for the ATP synthase CF1 α and β. The resulting values are presented in Table 2.

**Figure 7:**
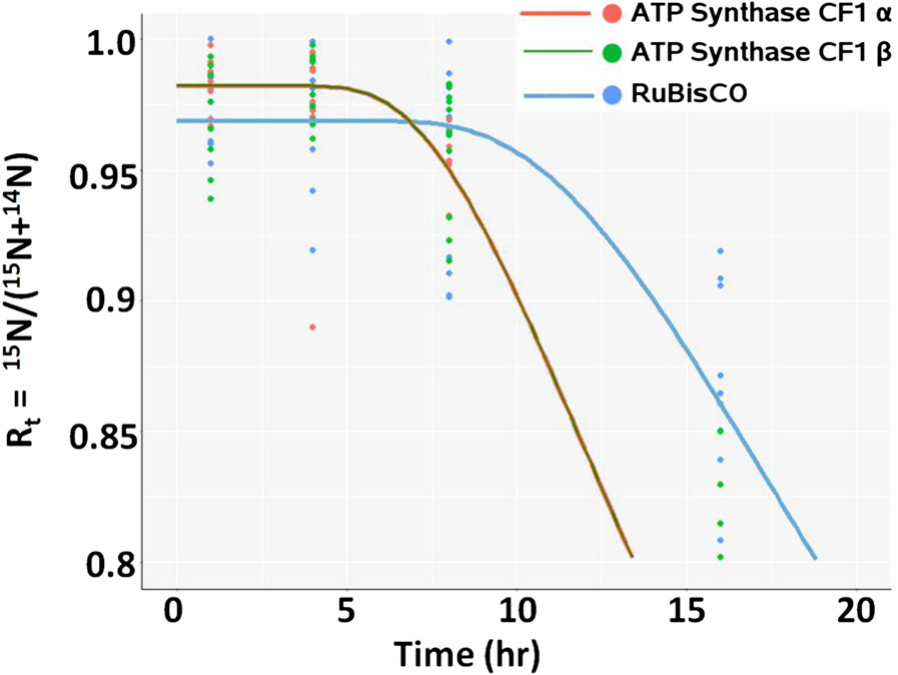
**Protein turnover within the initial 20 hr.** This is a close-up of the first 20 hr of the data displayed in Figure 6A. The curves for ATP synthase CF1 α and β are superimposed. There is no significant change in R_t_ for any of the three proteins in the first 5 hr. However, the initial R_t_ value for RuBisCo (blue curve) remains effectively unchanged until about 7.5 hr.

The regression model for all three proteins displayed a delay before rapid turnover began. The delay can be seen within the first 20 hr (Figure 7). For the R_t_ value to remain constant over time, both degradation and synthesis as reflected by the ^15^N- and ^14^N- abundances must remain unchanged. With respect to degradation, the absence of loss of the ^15^N-label from these plastid proteins could reflect partitioning of these proteins from the protein degradation machinery. The five-minute delay for the ATP synthase CF1 subunits compared to the seven-minute delay for the RuBisCo large subunit suggests that the ATP synthase CF1 subunits may come into contact with the proteasome before RuBisCo. Future immunolocalization experiments coupled to protein turnover studies will allow for testing that hypothesis. With respect to synthesis, the absence of incorporation of ^14^N-label into newly synthesized plastid proteins could reflect a delay in transport of newly synthesized ^14^N-labeled amino acids into the chloroplast where these proteins are synthesized.

## Conclusions

The availability of the genome sequences and the deduced protein databases coupled to LC-MS/MS allows for ease of study of various proteins without the need to purify each protein to homogeneity. This is exemplified by our results in which we obtained peptides representing co-resolved proteins. The regression curves from which the turnover rates were determined (Figure 6) differ between RuBisCo and the ATP synthase CF1 subunits. These curves also differed from the growth curves (Figure 1). Thus we can be confident that we are observing protein turnover and not cellular doubling time.

The capacity to measure changing enrichments in a number of peptides of the same protein allows a more accurate assessment of the actual turnover rate since multiple measurements can be combined. The protein-turnover measurement procedure followed the temporal evolution of those peptides through their fully labeled, partially labeled and unlabeled forms that occurs after labeled nutrients are substituted for unlabeled nutrients. Knowing the rates of protein turnover is critical to understanding the cellular regulatory processes that allow cells to respond to changing environmental conditions. We are now positioned to study the impact of various physiological conditions on protein turnover.

## Methods

### Algal strain and culture conditions

*Chlamydomonas reinhardtii* (CC-125 wild type mt + [137c]) was obtained from the Chlamydomonas Center culture collection. CC-125 cannot grow on nitrate as the sole N source, since it carries the nit1 and nit2 mutations. It was grown and maintained in Tris Acetate Phosphate medium (TAP) [15]. Algal growth was assessed daily by measuring the OD_600_ of each culture.

#### ^15^N-growth medium

Ammonium chloride (NH_4_Cl) in TAP salt stock was replaced with [^15^N]-ammonium nitrate (^15^NH_4_^15^NO_3_, 23.91 g/L). Ammonium molybdate (NH_4_)_6_Mo_7_O_24_.4H_2_O in Hunter’s trace elements was replaced with molybdic acid Na_2_MoO_4_.2H_2_O (1.51 g/L) as described [16]. For ^2^H_2_O labeling, water in the TAP medium was replaced with ^2^H_2_O [17]. Stable isotopes were all obtained from Cambridge Isotope Laboratories (Andover, MA).

#### ^15^N-labeling of algae

Colonies of algal cells from plates composed of ^14^N-TAP medium were used to inoculate 50 mL of ^15^N-TAP medium. After greater than seven doublings, 10 ml cell cultures were pelleted at 3000 g and resuspended in fresh ^15^N-TAP medium (50 mL). These samples were shaken under cool-white fluorescent lamps (33 μmol photons m^−2^ s^−1^) with a 16:8 hr light: dark cycle. This light regime was chosen, since it allows for synchronization of the algal cells which results in all cells being of the same size. The cultures were grown for more than seven doublings to ensure maximum label incorporation. Aliquots (10 mL) of the saturated cultures were removed; cells were collected by centrifugation at 3,000 g and resuspended in 50 mL of ^15^N-TAP medium. Cells were grown to saturation and transferred to ^15^N-TAP medium an additional time before switching to ^14^N-TAP medium to initiate the turnover experiments.

### Turnover experiment

Cells from ^15^N-TAP (50 mL) were collected by centrifugation at 3000 g and resuspended in 30 mL of ^14^N-TAP medium. Two minutes prior to the collection for each time point (4, 8, 12, 18, 30 min, 1, 2, 4, 8, 16, 32, 64 and 128 hours), 1.5 mL aliquots were transferred to microcentrifuge tubes, and centrifuged at 14,000 g for 30 s. The medium was discarded and the cell pellets quickly frozen in liquid nitrogen and subsequently placed at −80°C.

### Amino acids analysis

Amino acid purification, derivatization, GC-MS and data analysis were performed as described [18]. To the frozen algal pellets (10–50 mg wet weight) for each time point, 250 μL of 0.01 M HCl was added. Each tube was sonicated twice for 20 s and shaken for 45 min at room temperature. Samples were centrifuged at 14,000 g for 3 min and supernatants transferred to new tubes. The free amino acids were bound to a column of Dowex 50 W X2-200 resin (Sigma, Saint Louis, MO). The column was rinsed five times with 80% methanol and the amino acids eluted with 4 M NH_4_OH/50% MeOH. The free amino acids were derivatized with methyl chloroformate (MCF) [18]. The experimental samples and controls were run on a Hewlett Packard 5890/5970 GC-MS equipped with a 30 meter column (HP-5MS, 30 m × 25 mm ID, 0.25 μm film thickness, Agilent J&W Scientific, Folsom, CA) as follows: injection temperature: 240°C, oven temperature: 70°C for two minutes then 25°C/min increase until 280°C, hold for five minutes.

### Protein and peptide analysis

#### Isolation of 55 kDa band from SDS-PAGE

Algal pellets (50 mg wet weight) were resuspended in 100 μL of PBS-5% SDS buffer, heated at 95°C for one minute, centrifuged at 14,000 g for 10 min and the supernatants transferred to clean tubes. Samples were mixed with equal volumes of buffer [120 mM Tris–HCl pH 6.8, 4% (w/v) SDS, 20% (w/v) sucrose, 0.1% (w/v) bromophenol blue, 1% (v/v) β-mercaptoethanol], heated for 5 min at 95°C and resolved on a 7.5% SDS-PAGE gel at 200 V for 45 min. Protein bands were visualized by standard Coomassie staining and destaining. The band corresponding to the large subunit of RuBisCo (55 kDa) was excised from the gel, washed with 200 μL of 50 mM NH_4_HCO_3_/50% (v/v) acetonitrile, dehydrated with 100 μL acetonitrile and air dried for 10 min. The gel pieces were stored at −20°C until they were shipped on dry ice to the Center for Mass Spectrometry and Proteomics, at UMN, St. Paul, MN.

#### In-gel digestion and LC-MS/MS analysis

The excised bands from unlabeled and labeled samples were subjected to enzymatic trypsin digestion using a ProPrep™ system (Genomic Solutions, Ann Arbor, MI, USA). All digested extracts were evaporated to dryness *in vacuo* (SC210A SpeedVac® Plus, ThermoSavant, Asheville, NC USA), resuspended in LC-MS/MS loading buffer (98% H_2_O, 2% acetonitrile and 0.1% formic acid), and analyzed on an LC-MS/MS using a QSTAR Pulsar *i* quadrupole-TOF MS instrument (Applied Biosystems, Foster City, CA). MS/MS data were assigned using ProteinPilot (AB Sciex, Farmingham, MA) and using the non-redundant *Chlamydomonas reinhardtii* protein sequence database. Peptide identifications were accepted if they could be established at greater than 5% probability as specified by the Peptide Prophet algorithm [19]. Protein identifications were accepted if they could be established at greater than 20% probability and contained at least two identified peptide. Protein probabilities were assigned by the Protein Prophet algorithm [19]. Proteins that contained similar peptides and could not be differentiated based on MS/MS analysis alone were grouped to satisfy the principles of parsimony [20].

### Protein turnover analysis

From the MS data, isotopic distributions were generated representing the fully labeled (old peptide), fully unlabeled (spiked control). This was achieved by mixing the samples 1:1 (unlabeled protein: protein at a time point) based on total protein concentration (Bradford assay). The old versus new peptide distributions are binomial or beta binomial distributions (old and new respectively) that are fitted to the data using a maximum likelihood estimation. The ratio of the areas under the distributions provides relative quantities of partially labeled (newly synthesized) peptides at different time points of the chase experiment (Figure 5).

### Statistical analyses

The fractional abundance data was generated with the equation {R_t_ = ^15^N/(^15^N + ^14^N}. The abundance data was obtained from the MS spectral analyses using an in-house script written with the program R. In the regression analyses, fractional abundance data for each of the peptides for each protein was used in the graphing. CurveExpert professional software was used to identify the model with the best fit. The statistical analysis software (SAS) was used to calculate the parameters and t_1/2_ values.

## Competing interests

The authors declare that they have no competing interest.

## Supplementary Material

Additional file 1 List of peptides for each of the protein identified from the search of the Chlamydomonas database in order of confidence levels (largest to smallest). Click here for file

Additional file 2 List of proteins with a minimum of two unique peptides with percent confidence levels of 95 and greater. Click here for file

Additional file 3: Table S1 Identification of unique peptides and percent coverage for accepted proteins. Click here for file

Additional file 4 **List of peptides identified for each of the five proteins at various time points in the experiments.** Click here for file

Additional file 5 List of the peptides for the three plastid proteins. Click here for file
