# Supplementary material for: Metabolic labeling with stable isotope nitrogen (15N) to follow amino acid and protein turnover of three plastid proteins in Chlamydomonas reinhardtii
Source: Proteome Sci. 2014 Mar 3;12:14. doi: 10.1186/1477-5956-12-14 (PMC3943399; doi:10.1186/1477-5956-12-14)
Supplement: Additional file 1 — List of peptides for each of the protein identified from the search of the Chlamydomonas database in order of confidence levels (largest to smallest). [file 1477-5956-12-14-S1.pdf]

**ATP synthase CF1 alpha subunit**									
N	Unused	Total	%Cov	%Cov(50)	%Cov(95)	Accessions	Contr	Conf	Sequence
1	41.26	41.26	66.53543	47.44095	47.44095	gi|4117905	2	99	AIESPAPGI
1	41.26	41.26	66.53543	47.44095	47.44095	gi|4117905	2	99	ASSVAQVL
1	41.26	41.26	66.53543	47.44095	47.44095	gi|4117905	2	99	DLIEQYTPE
1	41.26	41.26	66.53543	47.44095	47.44095	gi|4117905	2	99	EILKQPQSS
1	41.26	41.26	66.53543	47.44095	47.44095	gi|4117905	2	99	IAEIPVGEA
1	41.26	41.26	66.53543	47.44095	47.44095	gi|4117905	2	99	LELAQFAEL
1	41.26	41.26	66.53543	47.44095	47.44095	gi|4117905	2	99	LKLELAQFA
1	41.26	41.26	66.53543	47.44095	47.44095	gi|4117905	2	99	LREILKQPQ
1	41.26	41.26	66.53543	47.44095	47.44095	gi|4117905	2	99	MVDFGIVF
1	41.26	41.26	66.53543	47.44095	47.44095	gi|4117905	2	99	QAINEYLEE
1	41.26	41.26	66.53543	47.44095	47.44095	gi|4117905	2	99	QAINEYLEE
1	41.26	41.26	66.53543	47.44095	47.44095	gi|4117905	2	99	QPQSSPLS
1	41.26	41.26	66.53543	47.44095	47.44095	gi|4117905	2	99	RSVYEPLAT
1	41.26	41.26	66.53543	47.44095	47.44095	gi|4117905	2	99	STLTFTPEA
1	41.26	41.26	66.53543	47.44095	47.44095	gi|4117905	2	99	SVYEPLAT
1	41.26	41.26	66.53543	47.44095	47.44095	gi|4117905	2	99	TAIAVDTIL
1	41.26	41.26	66.53543	47.44095	47.44095	gi|4117905	2	99	TPEELSNLI
1	41.26	41.26	66.53543	47.44095	47.44095	gi|4117905	2	99	TPEELSNLI
1	41.26	41.26	66.53543	47.44095	47.44095	gi|4117905	2	99	VVDGLARP
1	41.26	41.26	66.53543	47.44095	47.44095	gi|4117905	1.7	98	SYLANSYPK
1	41.26	41.26	66.53543	47.44095	47.44095	gi|4117905	1.4	96	EAYPGDVF
1	41.26	41.26	66.53543	47.44095	47.44095	gi|4117905	0.12	25	ELIIGDR
1	41.26	41.26	66.53543	47.44095	47.44095	gi|4117905	0.02	5.56	GLRPAINV
1	41.26	41.26	66.53543	47.44095	47.44095	gi|4117905	0.01	3	EMSLLLR
1	41.26	41.26	66.53543	47.44095	47.44095	gi|4117905	0	99	ASSVAQVL
1	41.26	41.26	66.53543	47.44095	47.44095	gi|4117905	0	99	LELAQFAEL
1	41.26	41.26	66.53543	47.44095	47.44095	gi|4117905	0	99	LKLELAQFA
1	41.26	41.26	66.53543	47.44095	47.44095	gi|4117905	0	99	LKLELAQFA
1	41.26	41.26	66.53543	47.44095	47.44095	gi|4117905	0	99	LKLELAQFA
1	41.26	41.26	66.53543	47.44095	47.44095	gi|4117905	0	99	MVDFGIVF
1	41.26	41.26	66.53543	47.44095	47.44095	gi|4117905	0	99	QAINEYLEE
1	41.26	41.26	66.53543	47.44095	47.44095	gi|4117905	0	99	QPQSSPLS
1	41.26	41.26	66.53543	47.44095	47.44095	gi|4117905	0	99	STLTFTPEA
1	41.26	41.26	66.53543	47.44095	47.44095	gi|4117905	0	99	STLTFTPEA
1	41.26	41.26	66.53543	47.44095	47.44095	gi|4117905	0	99	SVYEPLAT
1	41.26	41.26	66.53543	47.44095	47.44095	gi|4117905	0	99	TPEELSNLI
1	41.26	41.26	66.53543	47.44095	47.44095	gi|4117905	0	99	TPEELSNLI
1	41.26	41.26	66.53543	47.44095	47.44095	gi|4117905	0	99	TPEELSNLI
1	41.26	41.26	66.53543	47.44095	47.44095	gi|4117905	0	98	QAINEYLEE
1	41.26	41.26	66.53543	47.44095	47.44095	gi|4117905	0	91	QAINEYLEE
1	41.26	41.26	66.53543	47.44095	47.44095	gi|4117905	0	89	QAINEYLEE
1	41.26	41.26	66.53543	47.44095	47.44095	gi|4117905	0	86	EAYPGDVF
1	41.26	41.26	66.53543	47.44095	47.44095	gi|4117905	0	63	SVYEPLAT
1	41.26	41.26	66.53543	47.44095	47.44095	gi|4117905	0	58	VVDGLARP

1	41.26	41.26	66.53543	47.44095	47.44095	gi|4117905	0	57	TAIAVDTIL
1	41.26	41.26	66.53543	47.44095	47.44095	gi|4117905	0	56	SVYEPLATG
1	41.26	41.26	66.53543	47.44095	47.44095	gi|4117905	0	37	VVDGLARP
1	41.26	41.26	66.53543	47.44095	47.44095	gi|4117905	0	34	EAYPGDVF
1	41.26	41.26	66.53543	47.44095	47.44095	gi|4117905	0	13	RSVYEPLAT
1	41.26	41.26	66.53543	47.44095	47.44095	gi|4117905	0	4	STLTFTPEA
1	41.26	41.26	66.53543	47.44095	47.44095	gi|4117905	0	1	EMSLLLR
1	41.26	41.26	66.53543	47.44095	47.44095	gi|4117905	0	1	EMSLLLR
1	41.26	41.26	66.53543	47.44095	47.44095	gi|4117905	0	0	AMRTPEEL
1	41.26	41.26	66.53543	47.44095	47.44095	gi|4117905	0	0	ASDLDQAT
1	41.26	41.26	66.53543	47.44095	47.44095	gi|4117905	0	0	ASSVAQVL
1	41.26	41.26	66.53543	47.44095	47.44095	gi|4117905	0	0	DQATQNQ
1	41.26	41.26	66.53543	47.44095	47.44095	gi|4117905	0	0	EMSLLLR
1	41.26	41.26	66.53543	47.44095	47.44095	gi|4117905	0	0	GARLREIL
1	41.26	41.26	66.53543	47.44095	47.44095	gi|4117905	0	0	IGDRQTGK
1	41.26	41.26	66.53543	47.44095	47.44095	gi|4117905	0	0	LANSYPKY
1	41.26	41.26	66.53543	47.44095	47.44095	gi|4117905	0	0	LATGLVAV
1	41.26	41.26	66.53543	47.44095	47.44095	gi|4117905	0	0	NVGISVSR
1	41.26	41.26	66.53543	47.44095	47.44095	gi|4117905	0	0	SYLANSYPK
1	41.26	41.26	66.53543	47.44095	47.44095	gi|4117905	0	0	TAIAVDTIL
1	41.26	41.26	66.53543	47.44095	47.44095	gi|4117905	0	0	TAIAVDTIL
1	41.26	41.26	66.53543	47.44095	47.44095	gi|4117905	0	0	TAIAVDTIL
1	41.26	41.26	66.53543	47.44095	47.44095	gi|4117905	0	0	TAIAVDTIL
1	41.26	41.26	66.53543	47.44095	47.44095	gi|4117905	0	0	TAIAVDTIL
1	41.26	41.26	66.53543	47.44095	47.44095	gi|4117905	0	0	TGATLAEY
1	41.26	41.26	66.53543	47.44095	47.44095	gi|4117905	0	0	VMANANE

Modifications	Cleavages	dMass	Prec MW	Prec m/z	Theor MW	Theor m/z	Theor z
VAR		-0.00361	1179.658	590.8361	1179.661	590.8379	2
NTLK		-0.00116	1229.697	615.8557	1229.698	615.8563	2
VK		-0.00423	1333.672	667.8434	1333.677	667.8456	2
Deamidated(Q)@16	missed K-Q	0.026003	4076.121	1020.038	4076.095	1020.031	4
YLGR		0.004087	1386.755	694.3847	1386.751	694.3826	2
EAFSQFASDLDQATQNQLAR		0.00997	3253.599	1085.54	3253.589	1085.537	3
ELEAFSQFASDLDQATQNQLAR	missed K-L(	0.018749	3494.787	1165.936	3494.768	1165.93	3
SSPLSVEEQVASLYAGTNGYLDKLEVSQV	missed R-E	0.111451	4344.408	869.8889	4344.297	869.8666	5
Oxidation(M)@1		-0.0024	1738.869	870.4417	1738.871	870.4429	2
Gln->pyro-Glu@N-term		0.006669	1365.652	683.8333	1365.645	683.8299	2
FKSQAK	missed K-S(	0.016948	1796.911	599.9777	1796.894	599.9721	3
Deamidated(Q)@12	missed K-L(	0.00305	3592.793	1198.605	3592.79	1198.604	3
GLVAVDAMIPVGR	missed R-S(	0.013437	2313.265	772.0956	2313.251	772.0911	3
EGLVK		0.004831	1491.787	746.9008	1491.782	746.8983	2
Oxidation(M)@16		0.010154	2173.155	725.3924	2173.145	725.389	3
NQK		-0.00161	1285.723	643.8685	1285.724	643.8694	2
K		-0.01766	1142.601	572.3076	1142.618	572.3164	2
KDLIEQYTPEVK		0.012821	2458.297	820.4396	2458.284	820.4354	3
VDGK		-0.00179	1224.681	409.2342	1224.683	409.2348	3
		0.003406	1041.517	521.7656	1041.513	521.7638	2
YLHSR		-0.01455	1552.717	518.5795	1552.731	518.5843	3
		-0.0067	814.4482	408.2314	814.4549	408.2347	2
GISVSR	cleaved S-G	0.001625	1437.843	480.2884	1437.842	480.2878	3
Glu->pyro-Glu@N-term		-0.01144	842.457	422.2357	842.4684	422.2415	2
NTLK		0.004696	1229.703	615.8586	1229.698	615.8563	2
EAFSQFASDLDQATQNQLAR		0.016196	3253.605	1085.542	3253.589	1085.537	3
Deamidated(Q)@16	missed K-L(	0.031982	3495.784	1166.269	3495.752	1166.258	3
ELEAFSQFASDLDQATQNQLAR	missed K-L(	-0.00103	3494.767	1165.93	3494.768	1165.93	3
Deamidated(Q)@23	missed K-L(	0.000852	3495.753	1166.258	3495.752	1166.258	3
QVGDGIAR		-0.01019	1722.866	575.296	1722.876	575.2994	3
Gln->pyro-Glu@N-term	missed K-S(	0.008012	1779.876	890.9453	1779.868	890.9412	2
Gln->pyro-Glu@N-term; Deamidated(	missed K-L(	0.056953	3575.82	1192.947	3575.763	1192.928	3
EGLVK		0.006296	1491.788	746.9015	1491.782	746.8983	2
EGLVK		0.022288	1491.804	746.9095	1491.782	746.8983	2
LVAVDAMIPVGR		0.00312	2157.154	720.0585	2157.15	720.0574	3
K		-0.00887	1142.609	572.312	1142.618	572.3164	2
KDLIEQYTPEVK		-0.00201	2458.282	820.4347	2458.284	820.4354	3
KDLIEQYTPEVK		-0.00531	2458.279	820.4336	2458.284	820.4354	3
FK		0.007091	1382.679	692.3467	1382.672	692.3432	2
Gln->pyro-Glu@N-term		0.002641	1365.648	683.8312	1365.645	683.8299	2
FK		-0.00304	1382.669	692.3417	1382.672	692.3432	2
YLHSR		-0.00045	1552.731	518.5842	1552.731	518.5843	3
Trioxidation(Y)@3; Carbamidomethyl(E)@4; Deth		0.0277	2214.181	739.0676	2214.153	739.0583	3
VDGK		0.012859	1224.695	409.2391	1224.683	409.2348	3

NQK		-6.03943	1279.685	427.5688	1285.724	429.582	3
Oxidation(M)@16		0.005393	2173.151	725.3908	2173.145	725.389	3
VDGK		0.008648	1224.691	409.2377	1224.683	409.2348	3
Glu->pyro-Glu@N-term		0.014583	1534.735	512.5856	1534.72	512.5808	3
Oxidation(M)@17	missed R-S	0.003441	2329.25	777.4239	2329.246	777.4227	3
Cation:K(E)@8		-0.00187	1529.736	510.9193	1529.738	510.9199	3
		-0.0042	860.4748	431.2447	860.479	431.2468	2
		-0.00237	860.4766	431.2456	860.479	431.2468	2
SNLIK	cleaved M-	-0.06477	1500.732	751.3734	1500.797	751.4058	2
QNQLAR	cleaved F-A	-0.00731	1529.736	510.9193	1529.743	510.9217	3
Deamidated(N)@28	cleaved P-Y	-0.12196	4045.981	1012.503	4046.103	1012.533	4
Deamidated(Q)@2; Deamidated(Q)@	cleaved L-D	0.006785	1145.538	573.7763	1145.531	573.7729	2
Oxidation(M)@2		-0.0054	876.4685	439.2415	876.4739	439.2442	2
	cleaved L-K	-0.06882	926.4974	464.256	926.5661	464.2904	2
Oxidation(K)@8; Oxidation(K)@20	cleaved I-I@	-0.01482	2173.155	725.3924	2173.17	725.3974	3
Oxidation(P)@6	cleaved Y-L	-0.05118	1538.758	513.9266	1538.809	513.9437	3
Oxidation(P)@13	cleaved P-L	0.027386	1285.723	643.8685	1285.695	643.8549	2
Oxidation(R)@8	cleaved I-N	0.028669	846.4846	424.2496	846.4559	424.2352	2
		0.082634	1041.596	521.8052	1041.513	521.7638	2
Oxidation(N)@10	missed K-G	0.017402	1486.853	744.4337	1486.836	744.425	2
Deamidated(N)@10; Oxidation(K)@1	missed K-G	0.027339	1487.847	744.9307	1487.82	744.917	2
Oxidation(N)@10	missed K-G	0.00495	1486.84	744.4275	1486.836	744.425	2
NQKGK	missed K-G	0.011405	1470.852	736.4333	1470.841	736.4276	2
NQKGK	missed K-G	0.016288	1470.857	736.4357	1470.841	736.4276	2
Deamidated(R)@14	cleaved Y-T	-0.00494	2827.358	943.4598	2827.363	943.4615	3
Carbamidomethyl@N-term; Deamida	cleaved I-V	0.066022	902.4464	452.2305	902.3804	452.1975	2

Sc	Spectrum	Time
16	1.1.1.790.2	24.7116
15	1.1.1.855.4	39.3411
14	1.1.1.837.2	35.1562
16	1.1.1.996.3	57.2609
16	1.1.1.843.3	36.6026
24	1.1.1.1289.	71.1748
24	1.1.1.1185.	67.7608
20	1.1.1.975.2	55.5563
20	1.1.1.922.3	51.7456
14	1.1.1.905.3	48.8153
16	1.1.1.851.3	38.36
22	1.1.1.1009.	58.4368
19	1.1.1.964.4	54.7948
17	1.1.1.846.3	37.3001
19	1.1.1.914.2	50.3455
15	1.1.1.836.3	34.9755
13	1.1.1.833.4	34.381
25	1.1.1.1185.	67.7093
15	1.1.1.767.2	20.5614
13	1.1.1.781.3	22.6741
13	1.1.1.829.4	33.4528
9	1.1.1.786.3	23.8343
14	1.1.1.821.3	31.5438
8	1.1.1.823.5	32.1105
17	1.1.1.852.3	38.5925
22	1.1.1.1286.	71.0461
21	1.1.1.1185.	67.8637
18	1.1.1.1187.	68.2246
18	1.1.1.1190.	68.5085
15	1.1.1.961.2	54.1995
17	1.1.1.887.3	46.2187
17	1.1.1.1058.	62.5863
17	1.1.1.847.5	37.6352
15	1.1.1.849.2	37.9461
19	1.1.1.1013.	59.0077
17	1.1.1.830.2	33.5819
25	1.1.1.1187.	68.3274
18	1.1.1.1188.	68.4048
13	1.1.1.858.4	39.9362
12	1.1.1.905.5	48.9181
12	1.1.1.861.5	40.6848
12	1.1.1.832.3	34.0974
15	1.1.1.889.5	46.7868
11	1.1.1.773.3	21.1284

13	1.1.1.835.5	34.846
12	1.1.1.917.4	51.1467
10	1.1.1.761.2	19.9958
11	1.1.1.830.4	33.6848
11	1.1.1.892.4	47.4334
11	1.1.1.847.3	37.5324
8	1.1.1.822.3	31.7757
8	1.1.1.825.3	32.4722
6	1.1.1.917.2	51.0439
6	1.1.1.847.3	37.5324
8	1.1.1.962.2	54.3295
6	1.1.1.763.2	20.1499
7	1.1.1.791.3	24.995
7	1.1.1.797.2	26.3361
8	1.1.1.914.2	50.3455
7	1.1.1.853.4	38.8763
7	1.1.1.836.3	34.9755
6	1.1.1.799.3	26.8515
5	1.1.1.809.5	28.9142
9	1.1.1.964.3	54.7434
7	1.1.1.970.2	55.1632
7	1.1.1.971.3	55.2931
8	1.1.1.1055.	62.146
5	1.1.1.1057.	62.4053
6	1.1.1.893.2	47.5633
9	1.1.1.788.4	24.35

ATP synthase CF1 beta subunit									
N	Unused	Total	%Cov	%Cov(50)	%Cov(95)	Accessions	Contrib	Conf	Sequence
2	36.48	36.48	62.72913	50.9165	42.76986	gi|4117905	2	99	AHGGVSVFAGVGER
2	36.48	36.48	62.72913	50.9165	42.76986	gi|4117905	2	99	DVNKQDVLFFIDNIFR
2	36.48	36.48	62.72913	50.9165	42.76986	gi|4117905	2	99	ELQDIIAILGLDELSEEDRLIVAR
2	36.48	36.48	62.72913	50.9165	42.76986	gi|4117905	2	99	FLSQPFFVAEVFTGSPGKYVSLAET
2	36.48	36.48	62.72913	50.9165	42.76986	gi|4117905	2	99	FVQAGAEVSALLGR
2	36.48	36.48	62.72913	50.9165	42.76986	gi|4117905	2	99	GMEVVDTGKPLSVPVGK
2	36.48	36.48	62.72913	50.9165	42.76986	gi|4117905	2	99	GQVPNIYNALTIR
2	36.48	36.48	62.72913	50.9165	42.76986	gi|4117905	2	99	IFNVLGEPVDNMGNVK
2	36.48	36.48	62.72913	50.9165	42.76986	gi|4117905	2	99	IVQIIGPVLDIVFAK
2	36.48	36.48	62.72913	50.9165	42.76986	gi|4117905	2	99	QDVLFFIDNIFR
2	36.48	36.48	62.72913	50.9165	42.76986	gi|4117905	2	99	TAPAFVDLDTR
2	36.48	36.48	62.72913	50.9165	42.76986	gi|4117905	2	99	TVLIMELINNIAK
2	36.48	36.48	62.72913	50.9165	42.76986	gi|4117905	2	99	VALTALTMAEYFR
2	36.48	36.48	62.72913	50.9165	42.76986	gi|4117905	2	99	YKELQDIIAILGLDELSEEDR
2	36.48	36.48	62.72913	50.9165	42.76986	gi|4117905	2	99	YKELQDIIAILGLDELSEEDRLIVAR
2	36.48	36.48	62.72913	50.9165	42.76986	gi|4117905	1.699	98	IGLFGGAGVGK
2	36.48	36.48	62.72913	50.9165	42.76986	gi|4117905	1.155	93	VVDLLAPYR
2	36.48	36.48	62.72913	50.9165	42.76986	gi|4117905	1.155	93	VVDLLAPYRR
2	36.48	36.48	62.72913	50.9165	42.76986	gi|4117905	1.097	92	LSIFETGIK
2	36.48	36.48	62.72913	50.9165	42.76986	gi|4117905	1.046	91	AVSMNPTEGLMR
2	36.48	36.48	62.72913	50.9165	42.76986	gi|4117905	0.328	53	VEETLPIHR
2	36.48	36.48	62.72913	50.9165	42.76986	gi|4117905	0	99	FVQAGAEVSALLGR
2	36.48	36.48	62.72913	50.9165	42.76986	gi|4117905	0	99	IVQIIGPVLDIVFAK
2	36.48	36.48	62.72913	50.9165	42.76986	gi|4117905	0	99	TVLIMELINNIAK
2	36.48	36.48	62.72913	50.9165	42.76986	gi|4117905	0	99	TVLIMELINNIAK
2	36.48	36.48	62.72913	50.9165	42.76986	gi|4117905	0	99	TVLIMELINNIAK
2	36.48	36.48	62.72913	50.9165	42.76986	gi|4117905	0	99	TVLIMELINNIAK
2	36.48	36.48	62.72913	50.9165	42.76986	gi|4117905	0	99	VALTALTMAEYFR
2	36.48	36.48	62.72913	50.9165	42.76986	gi|4117905	0	99	YKELQDIIAILGLDELSEEDR
2	36.48	36.48	62.72913	50.9165	42.76986	gi|4117905	0	99	YKELQDIIAILGLDELSEEDRLIVAR
2	36.48	36.48	62.72913	50.9165	42.76986	gi|4117905	0	98	TVLIMELINNIAK
2	36.48	36.48	62.72913	50.9165	42.76986	gi|4117905	0	97	FLSQPFFVAEVFTGSPGKYVSLAET
2	36.48	36.48	62.72913	50.9165	42.76986	gi|4117905	0	95	DVNKQDVLFFIDNIFR
2	36.48	36.48	62.72913	50.9165	42.76986	gi|4117905	0	93	AHGGVSVFAGVGER
2	36.48	36.48	62.72913	50.9165	42.76986	gi|4117905	0	81	FLSQPFFVAEVFTGSPGKYVSLAET
2	36.48	36.48	62.72913	50.9165	42.76986	gi|4117905	0	43	IGLFGGAGVGK
2	36.48	36.48	62.72913	50.9165	42.76986	gi|4117905	0	17	VVDLLAPYR
2	36.48	36.48	62.72913	50.9165	42.76986	gi|4117905	0	5	IFNVLGEPVDNMGNVK
2	36.48	36.48	62.72913	50.9165	42.76986	gi|4117905	0	1	GMEVVDTGKPLSVPVGK
2	36.48	36.48	62.72913	50.9165	42.76986	gi|4117905	0	0	GGKIGLFGGAGVGK
2	36.48	36.48	62.72913	50.9165	42.76986	gi|4117905	0	0	IFAGELDDLPEQAFYLVGNITEAISK
2	36.48	36.48	62.72913	50.9165	42.76986	gi|4117905	0	0	MPSAVGYQPTLATEMGGLQER
2	36.48	36.48	62.72913	50.9165	42.76986	gi|4117905	0	0	QPWILGEK
2	36.48	36.48	62.72913	50.9165	42.76986	gi|4117905	0	0	VVDLLAPYRRGGK

Modificatio	Cleavages	dMass	Prec MW	Prec m/z	Theor MW	Theor m/z	Theor z	Sc	Spectrum	Time
		-0.00554	1341.673	448.2318	1341.679	448.2336	3	14	1.1.1.793.5	25.5619
	missed K-Q	0.009026	1982.035	661.6857	1982.026	661.6827	3	18	1.1.1.1059.	62.7672
	missed R-L	0.01284	2722.488	908.5033	2722.475	908.499	3	18	1.1.1.1117.	65.5477
Formyl(K)@	missed K-Y	0.047831	3379.75	1127.59	3379.702	1127.574	3	19	1.1.1.1186.	68.0956
		0.003164	1416.776	709.3951	1416.773	709.3935	2	15	1.1.1.873.5	43.3725
Oxidation(M)@2		0.013953	1727.927	576.9828	1727.913	576.9782	3	18	1.1.1.796.3	26.1554
		0.011544	1457.811	729.9126	1457.799	729.9068	2	16	1.1.1.868.4	42.2098
		-0.01823	1744.864	873.4391	1744.882	873.4482	2	17	1.1.1.867.3	41.9772
		0.003147	1624	813.007	1623.996	813.0055	2	16	1.1.1.1057.	62.4566
		-0.00346	1525.789	763.902	1525.793	763.9037	2	14	1.1.1.1070.	63.987
		-0.0006	1204.608	603.3114	1204.609	603.3117	2	15	1.1.1.820.5	31.4146
Oxidation(M)@5		-0.00244	1486.84	744.4275	1486.843	744.4287	2	17	1.1.1.971.3	55.2931
Oxidation(M)@8		-0.00336	1500.761	751.3879	1500.765	751.3896	2	16	1.1.1.871.5	42.9075
		-0.00529	2461.253	821.4251	2461.259	821.4269	3	23	1.1.1.1034.	61.2208
	missed R-L	-0.01626	3013.617	1005.546	3013.634	1005.552	3	24	1.1.1.1063.	63.6454
		-0.00425	974.5507	488.2826	974.5549	488.2847	2	13	1.1.1.820.4	31.3633
		-0.0055	1044.591	523.3029	1044.597	523.3057	2	12	1.1.1.842.5	36.4729
	missed R-R	-0.00797	1200.69	401.2372	1200.698	401.2399	3	12	1.1.1.819.4	31.1314
		-0.00618	1006.564	504.2891	1006.57	504.2922	2	12	1.1.1.839.5	35.7753
Oxidation(M)@4; Oxid		-0.00606	1336.605	669.31	1336.612	669.313	2	13	1.1.1.774.3	21.36
		-0.04794	1092.545	547.2797	1092.593	547.3037	2	10	1.1.1.781.5	22.7769
		-0.00025	1416.772	709.3934	1416.773	709.3935	2	18	1.1.1.870.3	42.5722
		-0.00686	1623.99	813.002	1623.996	813.0055	2	16	1.1.1.1060.	62.9996
Oxidation(M)@5		0.010008	1486.853	744.4337	1486.843	744.4287	2	18	1.1.1.964.3	54.7434
		-0.00026	1470.848	736.4312	1470.848	736.4313	2	15	1.1.1.1047.	61.7772
		0.004012	1470.852	736.4333	1470.848	736.4313	2	17	1.1.1.1055.	62.146
		0.008895	1470.857	736.4357	1470.848	736.4313	2	15	1.1.1.1057.	62.4053
		-0.00606	1484.764	743.3891	1484.77	743.3921	2	19	1.1.1.916.3	50.8624
		-0.00126	2461.258	821.4265	2461.259	821.4269	3	22	1.1.1.1032.	61.0131
	missed R-L	0.009584	3013.643	754.4181	3013.634	754.4157	4	17	1.1.1.1062.	63.4131
Oxidation(M)@5; Dear		0.019946	1487.847	744.9307	1487.827	744.9207	2	14	1.1.1.970.2	55.1632
Formyl(K)@	missed K-Y	0.047831	3379.75	1127.59	3379.702	1127.574	3	15	1.1.1.1186.	67.9928
	missed K-Q	-0.00361	1982.023	661.6815	1982.026	661.6827	3	13	1.1.1.1061.	63.2836
Methyl(H)@2		-0.06156	1355.633	678.8238	1355.695	678.8546	2	14	1.1.1.816.2	30.384
Formyl(K)@	missed K-Y	0.077496	3379.779	1127.6	3379.702	1127.574	3	14	1.1.1.1187.	68.276
		-0.00767	974.5473	488.2809	974.5549	488.2847	2	10	1.1.1.823.4	32.0591
		-0.00807	1044.589	523.3016	1044.597	523.3057	2	9	1.1.1.845.4	37.1188
Oxidation(M)@12		-0.00687	1760.87	881.4422	1760.877	881.4456	2	10	1.1.1.844.5	36.9378
		0.00875	1711.927	571.6495	1711.918	571.6466	3	9	1.1.1.821.5	31.6465
	missed K-I@	-0.05066	1216.642	406.5547	1216.693	406.5715	3	5	1.1.1.819.2	31.0286
		0.010259	2852.459	951.8268	2852.448	951.8234	3	6	1.1.1.1584.	79.1948
Deamidated(Q)@19; D		0.086154	2237.121	746.7141	2237.034	746.6854	3	6	1.1.1.888.4	46.5029
Oxidation(\	cleaved L-C	0.019223	985.5425	493.7785	985.5233	493.7689	2	7	1.1.1.817.5	30.7188
Carbamyl(F	missed R-R	0.027298	1486.853	744.4337	1486.826	744.4201	2	8	1.1.1.964.3	54.7434

RuBisCo										
N	Unused	Total	%Cov	%Cov(50)	%Cov(95)	Accessions	Conti	Conf	Sequence	Modificatio
3	29.26	29.26	65.89473	30.52632	29.89474	gi|4117904	2	99	DDENVNSQPFMR	
3	29.26	29.26	65.89473	30.52632	29.89474	gi|4117904	2	99	DRFLFVAEAIYK	
3	29.26	29.26	65.89473	30.52632	29.89474	gi|4117904	2	99	ELGVPIIMHDYLTGGFTANTSLAIYC	Carbamidor
3	29.26	29.26	65.89473	30.52632	29.89474	gi|4117904	2	99	EVTLGFVDLMR	
3	29.26	29.26	65.89473	30.52632	29.89474	gi|4117904	2	99	EVTLGFVDLMRDDYVEK	Oxidation(N
3	29.26	29.26	65.89473	30.52632	29.89474	gi|4117904	2	99	EVTLGFVDLMRDDYVEKDR	Oxidation(N
3	29.26	29.26	65.89473	30.52632	29.89474	gi|4117904	2	99	FLFVAEAIYK	
3	29.26	29.26	65.89473	30.52632	29.89474	gi|4117904	2	99	GGLDFTKDDENVNSQPFMR	
3	29.26	29.26	65.89473	30.52632	29.89474	gi|4117904	2	99	GLLGCTIKPK	Carbamidor
3	29.26	29.26	65.89473	30.52632	29.89474	gi|4117904	2	99	LTYYTPDYVVR	
3	29.26	29.26	65.89473	30.52632	29.89474	gi|4117904	2	99	TFVGPPHGIQVER	
3	29.26	29.26	65.89473	30.52632	29.89474	gi|4117904	2	99	WSPELAAACEVWK	Carbamidor
3	29.26	29.26	65.89473	30.52632	29.89474	gi|4117904	1.7	98	DTDILAAFR	
3	29.26	29.26	65.89473	30.52632	29.89474	gi|4117904	1.7	98	FEFDTIDKL	
3	29.26	29.26	65.89473	30.52632	29.89474	gi|4117904	0.9	86	LGCTIKPK	Carbamidor
3	29.26	29.26	65.89473	30.52632	29.89474	gi|4117904	0.7	81	EIKFEFDTIDKL	
3	29.26	29.26	65.89473	30.52632	29.89474	gi|4117904	0.1	19	ALRLEDLR	
3	29.26	29.26	65.89473	30.52632	29.89474	gi|4117904	0.1	14.1	GLLLHIHR	
3	29.26	29.26	65.89473	30.52632	29.89474	gi|4117904	0.1	11	VALEACTQAR	Carbamidor
3	29.26	29.26	65.89473	30.52632	29.89474	gi|4117904	0	8	MSGGDHLHSGTVVGK	Methyl(S)@
3	29.26	29.26	65.89473	30.52632	29.89474	gi|4117904	0	5	AVYECLR	Carbamidor
3	29.26	29.26	65.89473	30.52632	29.89474	gi|4117904	0	2.92	LEDLRIPPAYVK	
3	29.26	29.26	65.89473	30.52632	29.89474	gi|4117904	0	1	MTPQLGVPPEECGAAVAAESSTGT	Oxidation(N
3	29.26	29.26	65.89473	30.52632	29.89474	gi|4117904	0	99	DDENVNSQPFMR	Oxidation(N
3	29.26	29.26	65.89473	30.52632	29.89474	gi|4117904	0	99	ELGVPIIMHDYLTGGFTANTSLAIYC	Oxidation(N
3	29.26	29.26	65.89473	30.52632	29.89474	gi|4117904	0	99	ELGVPIIMHDYLTGGFTANTSLAIYC	Carbamidor
3	29.26	29.26	65.89473	30.52632	29.89474	gi|4117904	0	99	EVTLGFVDLMRDDYVEKDR	
3	29.26	29.26	65.89473	30.52632	29.89474	gi|4117904	0	99	FLFVAEAIYK	
3	29.26	29.26	65.89473	30.52632	29.89474	gi|4117904	0	99	GGLDFTKDDENVNSQPFMR	Dethiometh
3	29.26	29.26	65.89473	30.52632	29.89474	gi|4117904	0	99	GGLDFTKDDENVNSQPFMR	Oxidation(N
3	29.26	29.26	65.89473	30.52632	29.89474	gi|4117904	0	99	LTYYTPDYVVR	
3	29.26	29.26	65.89473	30.52632	29.89474	gi|4117904	0	99	TFVGPPHGIQVER	Oxidation(F
3	29.26	29.26	65.89473	30.52632	29.89474	gi|4117904	0	99	TFVGPPHGIQVER	
3	29.26	29.26	65.89473	30.52632	29.89474	gi|4117904	0	99	WSPELAAACEVWK	Formaldehy
3	29.26	29.26	65.89473	30.52632	29.89474	gi|4117904	0	98	EVTLGFVDLMR	Dethiometh
3	29.26	29.26	65.89473	30.52632	29.89474	gi|4117904	0	98	FLFVAEAIYK	
3	29.26	29.26	65.89473	30.52632	29.89474	gi|4117904	0	97	FLFVAEAIYK	
3	29.26	29.26	65.89473	30.52632	29.89474	gi|4117904	0	97	FLFVAEAIYK	
3	29.26	29.26	65.89473	30.52632	29.89474	gi|4117904	0	97	LTYYTPDYVVR	
3	29.26	29.26	65.89473	30.52632	29.89474	gi|4117904	0	96	TFVGPPHGIQVER	Oxidation(F
3	29.26	29.26	65.89473	30.52632	29.89474	gi|4117904	0	95	FLFVAEAIYK	
3	29.26	29.26	65.89473	30.52632	29.89474	gi|4117904	0	94	EVTLGFVDLMRDDYVEK	
3	29.26	29.26	65.89473	30.52632	29.89474	gi|4117904	0	90	GLLGCTIKPK	Carbamidor
3	29.26	29.26	65.89473	30.52632	29.89474	gi|4117904	0	88	DTDILAAFR	

3	29.26	29.26	65.89473	30.52632	29.89474	gi|4117904	0	86	DTDILAAFR	
3	29.26	29.26	65.89473	30.52632	29.89474	gi|4117904	0	85	TFVGPPHGIQVER	Oxidation(P
3	29.26	29.26	65.89473	30.52632	29.89474	gi|4117904	0	84	FLFVAEAIYK	
3	29.26	29.26	65.89473	30.52632	29.89474	gi|4117904	0	79	FLFVAEAIYK	
3	29.26	29.26	65.89473	30.52632	29.89474	gi|4117904	0	74	DTDILAAFR	
3	29.26	29.26	65.89473	30.52632	29.89474	gi|4117904	0	69	EVTLGFVDLMR	Oxidation(M
3	29.26	29.26	65.89473	30.52632	29.89474	gi|4117904	0	68	FLFVAEAIYK	
3	29.26	29.26	65.89473	30.52632	29.89474	gi|4117904	0	59	DDENVNSQPFMR	Dethiometh
3	29.26	29.26	65.89473	30.52632	29.89474	gi|4117904	0	56	DTDILAAFR	
3	29.26	29.26	65.89473	30.52632	29.89474	gi|4117904	0	52	EVTLGFVDLMR	Dethiometh
3	29.26	29.26	65.89473	30.52632	29.89474	gi|4117904	0	51	DTDILAAFR	
3	29.26	29.26	65.89473	30.52632	29.89474	gi|4117904	0	47	EVTLGFVDLMR	Glu->pyro-G
3	29.26	29.26	65.89473	30.52632	29.89474	gi|4117904	0	46	FLFVAEAIYK	
3	29.26	29.26	65.89473	30.52632	29.89474	gi|4117904	0	14	FLFVAEAIYK	
3	29.26	29.26	65.89473	30.52632	29.89474	gi|4117904	0	10	ELGVPIIMHDYLTGGFTANTSLAIYC	Carbamidor
3	29.26	29.26	65.89473	30.52632	29.89474	gi|4117904	0	9	EVTLGFVDLMRDDYVEK	Dethiometh
3	29.26	29.26	65.89473	30.52632	29.89474	gi|4117904	0	9	GGLDFTKDDENVNSQPFMR	Phospho(T)
3	29.26	29.26	65.89473	30.52632	29.89474	gi|4117904	0	6	TFVGPPHGIQVER	Oxidation(P
3	29.26	29.26	65.89473	30.52632	29.89474	gi|4117904	0	3	EIKFEFDTIDKL	Trimethyl(K
3	29.26	29.26	65.89473	30.52632	29.89474	gi|4117904	0	2	EVTLGFVDLMRDDYVEKDR	Carbamidor
3	29.26	29.26	65.89473	30.52632	29.89474	gi|4117904	0	1	MSGGDHLHSGTVVGK	Methyl(T)@
3	29.26	29.26	65.89473	30.52632	29.89474	gi|4117904	0	0	AGAGFKAGVK	Oxidation(F
3	29.26	29.26	65.89473	30.52632	29.89474	gi|4117904	0	0	ALRLEDLR	Deamidated
3	29.26	29.26	65.89473	30.52632	29.89474	gi|4117904	0	0	AVYECLR	Carbamidor
3	29.26	29.26	65.89473	30.52632	29.89474	gi|4117904	0	0	DDENVNSQPFMR	Dethiometh
3	29.26	29.26	65.89473	30.52632	29.89474	gi|4117904	0	0	DLAREGGDVIR	
3	29.26	29.26	65.89473	30.52632	29.89474	gi|4117904	0	0	EIKFEFDTIDKL	Carbamyl(K
3	29.26	29.26	65.89473	30.52632	29.89474	gi|4117904	0	0	EVTLGFVDLMR	
3	29.26	29.26	65.89473	30.52632	29.89474	gi|4117904	0	0	FGDDACLQFGGGTLGHPWGNAPG	Carbamidor
3	29.26	29.26	65.89473	30.52632	29.89474	gi|4117904	0	0	GHYLNATAGTCEEMMK	Carbamidor
3	29.26	29.26	65.89473	30.52632	29.89474	gi|4117904	0	0	GHYLNATAGTCEEMMKR	Carbamidor
3	29.26	29.26	65.89473	30.52632	29.89474	gi|4117904	0	0	GHYLNATAGTCEEMMKR	Carbamidor
3	29.26	29.26	65.89473	30.52632	29.89474	gi|4117904	0	0	GHYLNATAGTCEEMMKR	Carbamidor
3	29.26	29.26	65.89473	30.52632	29.89474	gi|4117904	0	0	LGCTIKPK	Carbamidor
3	29.26	29.26	65.89473	30.52632	29.89474	gi|4117904	0	0	LTYYTPDYVVR	Cation:K(D)
3	29.26	29.26	65.89473	30.52632	29.89474	gi|4117904	0	0	MSGGDHLHSGTVVGK	Methyl(T)@
3	29.26	29.26	65.89473	30.52632	29.89474	gi|4117904	0	0	MSGGDHLHSGTVVGKLEGER	
3	29.26	29.26	65.89473	30.52632	29.89474	gi|4117904	0	0	TGGFTANTSLAI	Deamidated
3	29.26	29.26	65.89473	30.52632	29.89474	gi|4117904	0	0	WSPELAAACEVWK	Carbamidor
3	29.26	29.26	65.89473	30.52632	29.89474	gi|4117904	0	0	WSPELAAACEVWKEIKFEFDTIDKL	Carbamidor

Cleavag	dMass	Prec MW	Prec m/z	Theor MW	Theor m/z	The	Sc	Spectrum	Time
	0.001657	1450.616	726.3154	1450.615	726.3146	2	14	1.1.1.790.5	24.8657
missed	-0.00646	1470.781	491.2675	1470.787	491.2696	3	15	1.1.1.878.3	44.227
nethyl(	-0.01841	3011.47	1004.831	3011.488	1004.837	3	19	1.1.1.987.2	56.7067
	0.003751	1278.668	640.3412	1278.664	640.3394	2	14	1.1.1.902.5	48.22
missed	0.016003	2043.998	682.34	2043.982	682.3347	3	18	1.1.1.865.5	41.6149
missed	0.015721	2315.126	579.7888	2315.11	579.7849	4	24	1.1.1.855.3	39.2898
	-0.00779	1199.651	600.8329	1199.659	600.8368	2	14	1.1.1.880.3	44.5896
	0.010609	2168.99	724.004	2168.98	724.0005	3	23	1.1.1.841.5	36.2405
nethyl(	-0.00806	1085.619	543.8166	1085.627	543.8206	2	15	1.1.1.784.4	23.4214
	0.003219	1388.701	695.3577	1388.698	695.3561	2	15	1.1.1.832.2	34.046
	0.006651	1435.764	479.5952	1435.757	479.593	3	15	1.1.1.798.4	26.6709
nethyl(	-0.00515	1545.724	773.869	1545.729	773.8716	2	19	1.1.1.872.2	42.9857
	-8.22E-05	1020.524	511.2692	1020.524	511.2693	2	13	1.1.1.863.3	41.047
missed	-0.0157	1126.539	564.2767	1126.555	564.2846	2	13	1.1.1.870.5	42.6749
cleaved	-0.00268	915.5186	458.7665	915.5212	458.7679	2	13	1.1.1.784.5	23.4728
missed	-0.0112	1496.765	499.929	1496.776	499.9327	3	12	1.1.1.884.3	45.5205
missed	-0.00538	984.5663	493.2904	984.5716	493.2931	2	9	1.1.1.795.4	25.9747
cleaved	-0.00731	957.58	479.7972	957.5872	479.8009	2	10	1.1.1.795.5	26.0261
nethyl(	-0.00579	1131.565	566.7897	1131.571	566.7926	2	11	1.1.1.764.2	20.227
9	-0.01825	1494.707	499.2428	1494.725	499.2489	3	11	1.1.1.773.4	21.1798
nethyl(	-0.00068	909.4372	455.7259	909.4378	455.7262	2	9	1.1.1.778.4	22.0297
missed	0.016676	1412.819	471.9471	1412.803	471.9415	3	9	1.1.1.825.4	32.5236
I)@1; O	0.164136	4045.981	1012.503	4045.817	1012.462	4	13	1.1.1.962.2	54.3295
I)@11	0.008508	1466.618	734.3163	1466.61	734.3121	2	15	1.1.1.777.3	21.7467
I)@8; C	-0.02237	3027.461	1010.161	3027.483	1010.168	3	15	1.1.1.921.2	51.4614
nethyl(	0.000813	3011.489	1004.837	3011.488	1004.837	3	19	1.1.1.989.3	56.8641
missed	0.001424	2299.117	575.7865	2299.115	575.7861	4	16	1.1.1.889.3	46.6841
	0.00649	1199.666	600.84	1199.659	600.8368	2	13	1.1.1.915.4	50.6811
hyl(M)@	0.017624	2120.994	531.2557	2120.976	531.2514	4	17	1.1.1.811.4	29.3269
I)@18	0.009952	2184.985	729.3354	2184.975	729.3321	3	17	1.1.1.824.3	32.2401
	-0.00459	1388.693	695.3538	1388.698	695.3561	2	14	1.1.1.829.2	33.3499
)@5	-0.00637	1451.746	484.9225	1451.752	484.9246	3	16	1.1.1.787.2	24.0151
	-4.03096	1431.726	478.2494	1435.757	479.593	3	16	1.1.1.797.5	26.4903
ydeAddu	-0.00215	1557.726	779.8705	1557.729	779.8716	2	18	1.1.1.882.4	45.1063
hyl(M)@	-0.00162	1230.659	411.227	1230.661	411.2276	3	14	1.1.1.841.3	36.1377
	0.007833	1199.667	600.8407	1199.659	600.8368	2	13	1.1.1.890.2	46.8653
	-0.00657	1199.652	600.8335	1199.659	600.8368	2	13	1.1.1.882.5	45.1578
	0.001241	1199.66	600.8374	1199.659	600.8368	2	13	1.1.1.883.3	45.2878
	-0.0024	1388.695	695.3549	1388.698	695.3561	2	13	1.1.1.831.3	33.8654
)@5	-0.00701	1451.745	484.9223	1451.752	484.9246	3	13	1.1.1.792.5	25.3297
	-0.00059	1199.658	600.8365	1199.659	600.8368	2	12	1.1.1.887.5	46.3214
missed	0.013547	2028.001	677.0076	2027.987	677.0031	3	13	1.1.1.906.4	49.0994
nethyl(	-0.00119	1099.641	550.8279	1099.642	550.8285	2	13	1.1.1.785.5	23.7048
	0.005106	1020.529	511.2718	1020.524	511.2693	2	12	1.1.1.860.4	40.4011

	-0.00728	1020.517	511.2656	1020.524	511.2693	2	12	1.1.1.862.2	40.7631
)@5; Ox	-0.00261	1483.739	742.877	1483.742	742.8782	2	13	1.1.1.801.3	27.2126
	0.004659	1199.664	600.8391	1199.659	600.8368	2	12	1.1.1.884.5	45.6234
	0.007833	1199.667	600.8407	1199.659	600.8368	2	11	1.1.1.911.3	49.8526
	-0.00674	1020.517	511.2659	1020.524	511.2693	2	11	1.1.1.866.3	41.7446
M)@10	0.000592	1294.66	648.3372	1294.659	648.3368	2	12	1.1.1.863.4	41.0984
	-0.01353	1199.646	600.83	1199.659	600.8368	2	11	1.1.1.893.4	47.666
hyl(M)@	0.011755	1402.623	468.5483	1402.611	468.5444	3	10	1.1.1.736.2	19.1991
	-6.02545	1014.499	508.2566	1020.524	511.2693	2	12	1.1.1.861.4	40.6334
hyl(M)@	-0.00162	1230.659	411.227	1230.661	411.2276	3	12	1.1.1.841.4	36.1891
	-0.00692	1020.517	511.2658	1020.524	511.2693	2	10	1.1.1.869.5	42.4425
Glu@N-t	0.006176	1212.656	405.2261	1212.65	405.224	3	12	1.1.1.842.3	36.3701
	-0.0045	1199.655	600.8345	1199.659	600.8368	2	11	1.1.1.886.3	45.9859
	0.014425	1199.673	600.844	1199.659	600.8368	2	10	1.1.1.902.4	48.1686
methyl(D	0.024253	3068.534	768.1408	3068.51	768.1348	4	12	1.1.1.892.5	47.4848
missed	0.017876	1980.002	496.0078	1979.984	496.0033	4	11	1.1.1.848.3	37.765
@6; Car	0.104507	2226.042	557.5179	2225.938	557.4917	4	14	1.1.1.812.2	29.4561
)@5; Ox	-0.00786	1483.734	742.8743	1483.742	742.8782	2	11	1.1.1.793.4	25.5105
missed	-0.02774	1538.795	513.9391	1538.823	513.9483	3	11	1.1.1.903.3	48.3499
missed	0.029107	2356.166	590.0488	2356.137	590.0415	4	11	1.1.1.839.4	35.7239
11	-0.01459	1494.71	499.244	1494.725	499.2489	3	10	1.1.1.765.3	20.4069
missed	-0.03238	936.4705	469.2425	936.5029	469.2587	2	7	1.1.1.779.2	22.1587
missed	-0.01314	985.5425	493.7785	985.5556	493.7851	2	5	1.1.1.817.5	30.7188
methyl(C	0.050547	925.4833	463.7489	925.4328	463.7237	2	7	1.1.1.784.3	23.37
hyl(M)@	0.017706	1402.629	468.5503	1402.611	468.5444	3	6	1.1.1.720.2	18.6858
missed	0.019667	1199.646	600.83	1199.626	600.8202	2	4	1.1.1.893.4	47.666
missed	0.005519	1539.788	514.2698	1539.782	514.268	3	9	1.1.1.904.4	48.6339
	-0.02295	1278.641	427.221	1278.664	427.2287	3	5	1.1.1.821.4	31.5952
cleaved	-0.06065	5552.548	926.4319	5552.609	926.4421	6	7	1.1.1.1016.	59.5004
methyl(C	0.017701	1763.778	441.9519	1763.761	441.9475	4	9	1.1.1.768.3	20.7413
missed	0.000837	2013.872	504.4752	2013.871	504.4749	4	10	1.1.1.602.2	15.4568
missed	0.03038	2013.901	504.4825	2013.871	504.4749	4	11	1.1.1.614.2	15.8671
missed	0.002003	1983.862	496.9728	1983.86	496.9723	4	9	1.1.1.779.3	22.2101
cleaved	-0.00453	929.5323	465.7734	929.5368	465.7757	2	9	1.1.1.786.4	23.8857
@7	-0.00269	1426.651	476.5576	1426.653	476.5584	3	9	1.1.1.831.5	33.9682
11	-0.03116	1494.694	499.2385	1494.725	499.2489	3	9	1.1.1.766.2	20.4841
missed	-0.01351	2064.988	517.2542	2065.001	517.2575	4	7	1.1.1.873.4	43.3211
cleaved	0.069124	1152.635	577.325	1152.566	577.2904	2	7	1.1.1.881.3	44.8222
methyl(C	0.025343	1560.717	781.3659	1560.692	781.3532	2	8	1.1.1.874.2	43.4508
missed	0.00604	3066.547	767.6441	3066.541	767.6426	4	10	1.1.1.1371.	73.4427

Mt F1F0 ATP synthase alpha subunit gi 159483										
N	Unused	Total	%Cov	%Cov(50)	%Cov(95)	Con	Cor	Sequence	Modif	Cleav
4	26.57	27.51	68.89279	39.89455	33.91916	2	99	AVDALVPIGR		
4	26.57	27.51	68.89279	39.89455	33.91916	2	99	EVAAFAQFGSDLDAATQYVLER		
4	26.57	27.51	68.89279	39.89455	33.91916	2	99	GMALNLQADHVGVVVFGNDSLIHQGDLVYR		
4	26.57	27.51	68.89279	39.89455	33.91916	2	99	GYLDKVPVNQITACEDVILK	Carba	misse
4	26.57	27.51	68.89279	39.89455	33.91916	2	99	TAVALDCILHQNYLNGLTNKK	Carba	misse
4	26.57	27.51	68.89279	39.89455	33.91916	2	99	TGQIVNVPVGPGTLGR		
4	26.57	27.51	68.89279	39.89455	33.91916	2	99	VGSAAQFPGMK		
4	26.57	27.51	68.89279	39.89455	33.91916	2	99	VLSVGDGIAR		
4	26.57	27.51	68.89279	39.89455	33.91916	2	99	VVDALGQPIDGK		
4	26.57	27.51	68.89279	39.89455	33.91916	2	99	VVDALGQPIDGKGPLTNVR		misse
4	26.57	27.51	68.89279	39.89455	33.91916	1.7	98	HAVIIYDDLSK		
4	26.57	27.51	68.89279	39.89455	33.91916	1.4	96	LFAQTGALK		
4	26.57	27.51	68.89279	39.89455	33.91916	1.2	99	GIRPALNVGLSVSR		
4	26.57	27.51	68.89279	39.89455	33.91916	1	90	QYQPPVIHLGR		
4	26.57	27.51	68.89279	39.89455	33.91916	0.9	86	EAFPGDVFYLHSR		
4	26.57	27.51	68.89279	39.89455	33.91916	0.4	58	LIPAVKEWEK		
4	26.57	27.51	68.89279	39.89455	33.91916	0	8	LELAQYR		
4	26.57	27.51	68.89279	39.89455	33.91916	0	4	QMSLLLR		
4	26.57	27.51	68.89279	39.89455	33.91916	0	1	ITPEINAHLAQQMSNLPVMTK	Oxidation(M	
4	26.57	27.51	68.89279	39.89455	33.91916	0	99	AVDALVPIGR		
4	26.57	27.51	68.89279	39.89455	33.91916	0	99	VVDALGQPIDGK		
4	26.57	27.51	68.89279	39.89455	33.91916	0	82	EAFPGDVFYLHSR		
4	26.57	27.51	68.89279	39.89455	33.91916	0	77	QYQPPVIHLGR	Gln->pyro-G	
4	26.57	27.51	68.89279	39.89455	33.91916	0	45	EAFPGDVFYLHSR	Oxidation(F)	
4	26.57	27.51	68.89279	39.89455	33.91916	0	44	GMALNLQADHVGVVVFGNDSLIHQGDLVYR	Deamidated	
4	26.57	27.51	68.89279	39.89455	33.91916	0	43	EAFPGDVFYLHSR		
4	26.57	27.51	68.89279	39.89455	33.91916	0	42	EAFPGDVFYLHSR	Oxidation(F)	
4	26.57	27.51	68.89279	39.89455	33.91916	0	25	ELIIGDR		
4	26.57	27.51	68.89279	39.89455	33.91916	0	16	EAFPGDVFYLHSR	Glu->pyro-G	
4	26.57	27.51	68.89279	39.89455	33.91916	0	3	QMSLLLR	Gln->pyro-G	
4	26.57	27.51	68.89279	39.89455	33.91916	0	1	QMSLLLR	Deamidated	
4	26.57	27.51	68.89279	39.89455	33.91916	0	0	ARAGLLQL	Deam	cleave
4	26.57	27.51	68.89279	39.89455	33.91916	0	0	AVDALVPIGR	Carbamidon	
4	26.57	27.51	68.89279	39.89455	33.91916	0	0	ETQAGDVSAYIATNVISITDGQIFLETELFYKGIRPA	Deam	cleave
4	26.57	27.51	68.89279	39.89455	33.91916	0	0	GARLTEVLKQK	Deam	misse
4	26.57	27.51	68.89279	39.89455	33.91916	0	0	GDRQTGKTAVALDCILHQNYLNGLTNK	Carba	cleave
4	26.57	27.51	68.89279	39.89455	33.91916	0	0	GQRELIIGDRQTGKTAVALDCILHQNYLNGLTNKK	Deam	misse
4	26.57	27.51	68.89279	39.89455	33.91916	0	0	KSVQAGELVCFDSGVK	Carba	cleave
4	26.57	27.51	68.89279	39.89455	33.91916	0	0	KSVQAGELVCFDSGVK	Carba	cleave
4	26.57	27.51	68.89279	39.89455	33.91916	0	0	LELAQYREVAAFAQFGSDLDAATQYVLERGAR	Deam	misse
4	26.57	27.51	68.89279	39.89455	33.91916	0	0	LFAQTGALKYTII		cleave
4	26.57	27.51	68.89279	39.89455	33.91916	0	0	NVGLSVSR	Oxida	cleave
4	26.57	27.51	68.89279	39.89455	33.91916	0	0	QMSLLLR	Deamidated	
4	26.57	27.51	68.89279	39.89455	33.91916	0	0	QMSLLLR	Deamidated	

4	26.57	27.51	68.89279	39.89455	33.91916	0	0	QSVAYRQMSLLLR		misse
4	26.57	27.51	68.89279	39.89455	33.91916	0	0	QSVREPLYTGVK		misse
4	26.57	27.51	68.89279	39.89455	33.91916	0	0	QSVREPLYTGVKAVDALVPIGR	Deam	misse
4	26.57	27.51	68.89279	39.89455	33.91916	0	0	QVAGTLKLELAQY	Gln->|	cleave
4	26.57	27.51	68.89279	39.89455	33.91916	0	0	TAVALDCILHQNYLNGLTNK	Carbamidon	
4	26.57	27.51	68.89279	39.89455	33.91916	0	0	VGSAAQFPGMKQVAGTLK		misse
4	26.57	27.51	68.89279	39.89455	33.91916	0	0	VVDALGQPIDGKGPLTNVRSSLVEIK	Deam	misse

dMass	Prec MW	Prec m/z	Theor MW	Theor m/z	Theo	Sc	Spectrum	Time
0.008058	1009.6	505.8073	1009.592	505.8033	2	13	1.1.1.817.2	30.5646
0.024242	2400.184	801.0686	2400.16	801.0605	3	20	1.1.1.1057.	62.3025
0.034095	3236.674	810.1758	3236.64	810.1673	4	21	1.1.1.915.2	50.5783
0.002531	2274.196	759.0724	2274.193	759.0716	3	18	1.1.1.881.4	44.8736
0.071589	2385.271	597.3251	2385.2	597.3072	4	17	1.1.1.881.5	44.925
0.000606	1563.874	782.9442	1563.873	782.9439	2	19	1.1.1.835.2	34.6919
-0.00581	1091.538	546.7761	1091.543	546.779	2	15	1.1.1.788.5	24.4014
-0.01033	985.5453	493.7799	985.5556	493.7851	2	13	1.1.1.789.2	24.4793
0.001011	1210.657	606.3356	1210.656	606.3352	2	17	1.1.1.791.5	25.0977
0.001255	1948.075	650.3657	1948.074	650.3653	3	16	1.1.1.832.5	34.2002
0.005494	1272.677	637.3457	1272.671	637.343	2	14	1.1.1.796.4	26.2068
-0.00246	947.5416	474.778	947.544	474.7793	2	12	1.1.1.788.2	24.2473
0.001625	1437.843	480.2884	1437.842	480.2878	3	14	1.1.1.821.3	31.5438
-0.00672	1306.708	436.5766	1306.715	436.5788	3	12	1.1.1.809.2	28.7601
2.021989	1538.758	513.9266	1536.736	513.2527	3	13	1.1.1.853.4	38.8763
-0.00493	1211.687	404.9028	1211.691	404.9044	3	11	1.1.1.808.2	28.5281
0.00373	891.4852	446.7498	891.4814	446.748	2	8	1.1.1.788.3	24.2986
-0.00101	859.494	430.7542	859.495	430.7548	2	9	1.1.1.818.2	30.7966
-0.01383	2367.179	790.0669	2367.193	790.0715	3	10	1.1.1.822.4	31.8271
0.00287	1009.595	505.8047	1009.592	505.8033	2	14	1.1.1.814.2	29.9201
0.00333	1210.659	606.3368	1210.656	606.3352	2	15	1.1.1.790.4	24.8143
0.000939	1536.737	513.253	1536.736	513.2527	3	12	1.1.1.856.2	39.4709
0.00873	1289.697	645.8557	1289.688	645.8513	2	11	1.1.1.838.3	35.44
-0.01455	1552.717	518.5795	1552.731	518.5843	3	13	1.1.1.829.4	33.4528
0.010583	3237.635	810.416	3237.624	810.4133	4	12	1.1.1.915.5	50.7325
0.028223	1536.764	513.2621	1536.736	513.2527	3	11	1.1.1.859.2	40.0659
-0.00045	1552.731	518.5842	1552.731	518.5843	3	12	1.1.1.832.3	34.0974
-0.0067	814.4482	408.2314	814.4549	408.2347	2	9	1.1.1.786.3	23.8343
0.014583	1534.735	512.5856	1534.72	512.5808	3	11	1.1.1.830.4	33.6848
-0.01144	842.457	422.2357	842.4684	422.2415	2	8	1.1.1.823.5	32.1105
-0.00237	860.4766	431.2456	860.479	431.2468	2	8	1.1.1.825.3	32.4722
0.008348	841.5105	421.7625	841.5022	421.7584	2	8	1.1.1.802.4	27.3933
0.01627	1066.63	534.3221	1066.613	534.314	2	10	1.1.1.828.2	33.1177
0.007491	5616.907	937.1584	5616.899	937.1571	6	7	1.1.1.983.3	56.2892
-0.01284	1285.723	643.8685	1285.735	643.875	2	7	1.1.1.836.3	34.9755
0.039689	3047.549	762.8945	3047.509	762.8846	4	10	1.1.1.1063.	63.594
0.159615	5616.907	937.1584	5616.747	937.1318	6	9	1.1.1.983.3	56.2892
0.012861	1738.869	870.4417	1738.856	870.4353	2	7	1.1.1.922.3	51.7456
0.005063	1722.866	575.296	1722.861	575.2943	3	6	1.1.1.961.2	54.1995
0.066849	3575.82	1192.947	3575.753	1192.925	3	7	1.1.1.1058.	62.5863
0.020069	1437.843	480.2884	1437.823	480.2817	3	6	1.1.1.821.3	31.5438
0.028669	846.4846	424.2496	846.4559	424.2352	2	6	1.1.1.799.3	26.8515
-0.0054	876.4685	439.2415	876.4739	439.2442	2	7	1.1.1.791.3	24.995
-0.0042	860.4748	431.2447	860.479	431.2468	2	8	1.1.1.822.3	31.7757

0.018365	1563.874	782.9442	1563.856	782.935	2	5	1.1.1.835.2	34.6919
0.004213	1375.75	459.5907	1375.746	459.5893	3	6	1.1.1.782.3	22.9061
-0.04999	2369.245	790.7557	2369.295	790.7724	3	6	1.1.1.913.2	50.1128
0.022212	1416.772	709.3934	1416.75	709.3823	2	7	1.1.1.870.3	42.5722
0.064068	2274.196	759.0724	2274.131	759.0511	3	8	1.1.1.881.4	44.8736
-0.00206	1788.954	597.3251	1788.956	597.3258	3	6	1.1.1.881.5	44.925
0.004626	2737.491	913.5042	2737.486	913.5027	3	7	1.1.1.1174.	67.2632

S-Adenosyl homocysteine hydrolase gi 159470										
N	Unused	Total	%Cov	%Cov(50)	%Cov(95)	Contr	Conf	Sequence	Modific	Cleavag
5	16.98	16.98	36.853	31.88406	29.81367	2	99	ALSVDHVNGR	Protein	cleaved
5	16.98	16.98	36.853	31.88406	29.81367	2	99	DGTLPNPDSTDNAEFK		
5	16.98	16.98	36.853	31.88406	29.81367	2	99	FIFPDGHGVIVLAEGR		
5	16.98	16.98	36.853	31.88406	29.81367	2	99	ITGSLHMTIQTAVLIETLTALGAEVR		
5	16.98	16.98	36.853	31.88406	29.81367	2	99	LSADQAAYINVPVDGPYKPAHYR		
5	16.98	16.98	36.853	31.88406	29.81367	2	99	NNAIVGNIGHFDNEVDMAGLYAWPGI	Oxidation(M)@	
5	16.98	16.98	36.853	31.88406	29.81367	2	99	TAFIAGYGDVGK		
5	16.98	16.98	36.853	31.88406	29.81367	2	99	VMGVSEETTTGVKR		missed
5	16.98	16.98	36.853	31.88406	29.81367	0.92	88	SEFGPAQPFK		
5	16.98	16.98	36.853	31.88406	29.81367	0.06	13	HSLPDGIMR		
5	16.98	16.98	36.853	31.88406	29.81367	0	99	ITGSLHMTIQTAVLIETLTALGAEVR		
5	16.98	16.98	36.853	31.88406	29.81367	0	99	ITGSLHMTIQTAVLIETLTALGAEVR	Oxidation(M)@	
5	16.98	16.98	36.853	31.88406	29.81367	0	94	NNAIVGNIGHFDNEVDMAGLYAWPGI	Oxidation(N)@7	
5	16.98	16.98	36.853	31.88406	29.81367	0	86	FIFPDGHGVIVLAEGR		
5	16.98	16.98	36.853	31.88406	29.81367	0	57	NNAIVGNIGHFDNEVDMAGLYAWPGIK		
5	16.98	16.98	36.853	31.88406	29.81367	0	6	SEFGPAQPFK		
5	16.98	16.98	36.853	31.88406	29.81367	0	0	LSADQAAYINV	Deamid	cleaved
5	16.98	16.98	36.853	31.88406	29.81367	0	0	NNAIVGNIGHF		cleaved
5	16.98	16.98	36.853	31.88406	29.81367	0	0	NVNDSVTKSK	Oxidati	cleaved
5	16.98	16.98	36.853	31.88406	29.81367	0	0	SVDHVNGREYKVK		cleaved

dMass	Prec MW	Prec m/z	Theor MW	Theor m/z	Theor z	Sc	Spectrum	Time
0.007009	1108.57	555.2921	1108.563	555.2885	2	14	1.1.1.782.4	22.9574
0.004737	1719.764	860.889	1719.759	860.8867	2	19	1.1.1.799.2	26.8001
0.001732	1725.922	576.3146	1725.92	576.314	3	20	1.1.1.884.2	45.4691
-0.014	2737.491	913.5042	2737.505	913.5089	3	24	1.1.1.1174.	67.2632
0.022434	2544.299	637.0819	2544.276	637.0763	4	15	1.1.1.836.5	35.0783
0.019214	2930.421	977.8144	2930.402	977.808	3	17	1.1.1.921.3	51.5128
0.000419	1197.603	599.809	1197.603	599.8088	2	15	1.1.1.811.2	29.2241
0.00734	1492.763	498.5949	1492.756	498.5925	3	16	1.1.1.774.2	21.3086
0.002288	1106.542	554.2783	1106.54	554.2771	2	12	1.1.1.798.3	26.6195
0.001685	1024.514	513.2643	1024.512	513.2635	2	9	1.1.1.783.3	23.1381
0.006691	2737.511	913.5111	2737.505	913.5089	3	21	1.1.1.1172.	67.1589
0.009331	2753.509	918.8436	2753.5	918.8405	3	19	1.1.1.1064.	63.7234
0.014327	2914.403	972.4749	2914.389	972.4701	3	15	1.1.1.956.2	53.7547
-0.06053	1725.86	576.2938	1725.92	576.314	3	12	1.1.1.890.3	46.9167
-0.00924	2914.398	972.4733	2914.407	972.4763	3	12	1.1.1.955.2	53.6759
-0.00284	1106.537	554.2757	1106.54	554.2771	2	9	1.1.1.801.2	27.1612
-0.0248	1164.542	583.278	1164.566	583.2904	2	7	1.1.1.783.4	23.1895
0.002931	1154.586	578.3004	1154.583	578.2989	2	5	1.1.1.834.5	34.6136
0.007644	1106.564	554.2895	1106.557	554.2857	2	7	1.1.1.781.4	22.7255
-0.05895	1529.736	510.9193	1529.795	510.939	3	6	1.1.1.847.3	37.5324

Sequences for various other proteins

N	Unus	Total	%Cov	%Cov(50)	%Cov(95)	Accessions	Names	Contr	Conf
6	15	15	30.7	22.7991	20.54176	gi|1594718	beta tubulin 1 [Chlamydomonas reinhard	2	99
6	15	15	30.7	22.7991	20.54176	gi|1594718	beta tubulin 1 [Chlamydomonas reinhard	2	99
6	15	15	30.7	22.7991	20.54176	gi|1594718	beta tubulin 1 [Chlamydomonas reinhard	2	99
6	15	15	30.7	22.7991	20.54176	gi|1594718	beta tubulin 1 [Chlamydomonas reinhard	2	99
6	15	15	30.7	22.7991	20.54176	gi|1594718	beta tubulin 1 [Chlamydomonas reinhard	2	99
6	15	15	30.7	22.7991	20.54176	gi|1594718	beta tubulin 1 [Chlamydomonas reinhard	2	99
6	15	15	30.7	22.7991	20.54176	gi|1594718	beta tubulin 1 [Chlamydomonas reinhard	2	99
6	15	15	30.7	22.7991	20.54176	gi|1594718	beta tubulin 1 [Chlamydomonas reinhard	0	99
6	15	15	30.7	22.7991	20.54176	gi|1594718	beta tubulin 1 [Chlamydomonas reinhard	0.9	88
6	15	15	30.7	22.7991	20.54176	gi|1594718	beta tubulin 1 [Chlamydomonas reinhard	0.1	17
6	15	15	30.7	22.7991	20.54176	gi|1594718	beta tubulin 1 [Chlamydomonas reinhard	0	0
6	15	15	30.7	22.7991	20.54176	gi|1594718	beta tubulin 1 [Chlamydomonas reinhard	0	0
6	15	15	30.7	22.7991	20.54176	gi|1594718	beta tubulin 1 [Chlamydomonas reinhard	0	0
6	15	15	30.7	22.7991	20.54176	gi|1594718	beta tubulin 1 [Chlamydomonas reinhard	0	0

N	Unus	Total	%Cov	%Cov(50)	%Cov(95)	Accessions	Names	Contr	Conf
7	14	13.6	40.18	20.54054	18.55856	gi|1594893	acetohydroxy acid isomeroreductase [Ch	2	99
7	14	13.6	40.18	20.54054	18.55856	gi|1594893	acetohydroxy acid isomeroreductase [Ch	2	99
7	14	13.6	40.18	20.54054	18.55856	gi|1594893	acetohydroxy acid isomeroreductase [Ch	2	99
7	14	13.6	40.18	20.54054	18.55856	gi|1594893	acetohydroxy acid isomeroreductase [Ch	2	99
7	14	13.6	40.18	20.54054	18.55856	gi|1594893	acetohydroxy acid isomeroreductase [Ch	2	99
7	14	13.6	40.18	20.54054	18.55856	gi|1594893	acetohydroxy acid isomeroreductase [Ch	1.5	97
7	14	13.6	40.18	20.54054	18.55856	gi|1594893	acetohydroxy acid isomeroreductase [Ch	1.5	97
7	14	13.6	40.18	20.54054	18.55856	gi|1594893	acetohydroxy acid isomeroreductase [Ch	0.5	71
7	14	13.6	40.18	20.54054	18.55856	gi|1594893	acetohydroxy acid isomeroreductase [Ch	0	38
7	14	13.6	40.18	20.54054	18.55856	gi|1594893	acetohydroxy acid isomeroreductase [Ch	0	6.69
7	14	13.6	40.18	20.54054	18.55856	gi|1594893	acetohydroxy acid isomeroreductase [Ch	0	0.95
7	14	13.6	40.18	20.54054	18.55856	gi|1594893	acetohydroxy acid isomeroreductase [Ch	0	0
7	14	13.6	40.18	20.54054	18.55856	gi|1594893	acetohydroxy acid isomeroreductase [Ch	0	0
7	14	13.6	40.18	20.54054	18.55856	gi|1594893	acetohydroxy acid isomeroreductase [Ch	0	0
7	14	13.6	40.18	20.54054	18.55856	gi|1594893	acetohydroxy acid isomeroreductase [Ch	0	0
7	14	13.6	40.18	20.54054	18.55856	gi|1594893	acetohydroxy acid isomeroreductase [Ch	0	0
7	14	13.6	40.18	20.54054	18.55856	gi|1594893	acetohydroxy acid isomeroreductase [Ch	0	0
7	14	13.6	40.18	20.54054	18.55856	gi|1594893	acetohydroxy acid isomeroreductase [Ch	0	0

N	Unus	Total	%Cov	%Cov(50)	%Cov(95)	Accessions	Names	Contr	Conf
8	10	10.1	27.67	17.61006	17.61006	gi|1594899	enolase [Chlamydomonas reinhardtii]	2	99
8	10	10.1	27.67	17.61006	17.61006	gi|1594899	enolase [Chlamydomonas reinhardtii]	2	99
8	10	10.1	27.67	17.61006	17.61006	gi|1594899	enolase [Chlamydomonas reinhardtii]	2	99
8	10	10.1	27.67	17.61006	17.61006	gi|1594899	enolase [Chlamydomonas reinhardtii]	2	99
8	10	10.1	27.67	17.61006	17.61006	gi|1594899	enolase [Chlamydomonas reinhardtii]	2	99
8	10	10.1	27.67	17.61006	17.61006	gi|1594899	enolase [Chlamydomonas reinhardtii]	0	10.6
8	10	10.1	27.67	17.61006	17.61006	gi|1594899	enolase [Chlamydomonas reinhardtii]	0	0
8	10	10.1	27.67	17.61006	17.61006	gi|1594899	enolase [Chlamydomonas reinhardtii]	0	0

8	10	10.1	27.67	17.61006	17.61006	gi|1594899	enolase [Chlamydomonas reinhardtii]	0	0
8	10	10.1	27.67	17.61006	17.61006	gi|1594899	enolase [Chlamydomonas reinhardtii]	0	0
8	10	10.1	27.67	17.61006	17.61006	gi|1594899	enolase [Chlamydomonas reinhardtii]	0	0
8	10	10.1	27.67	17.61006	17.61006	gi|1594899	enolase [Chlamydomonas reinhardtii]	0	0
9	8.2	8.24	30.39	12.39389	12.39389	gi|1594770	fumarate hydratase [Chlamydomonas rei	2	99
9	8.2	8.24	30.39	12.39389	12.39389	gi|1594770	fumarate hydratase [Chlamydomonas rei	2	99
9	8.2	8.24	30.39	12.39389	12.39389	gi|1594770	fumarate hydratase [Chlamydomonas rei	2	99
9	8.2	8.24	30.39	12.39389	12.39389	gi|1594770	fumarate hydratase [Chlamydomonas rei	2	99
9	8.2	8.24	30.39	12.39389	12.39389	gi|1594770	fumarate hydratase [Chlamydomonas rei	0.2	39
9	8.2	8.24	30.39	12.39389	12.39389	gi|1594770	fumarate hydratase [Chlamydomonas rei	0	5.67
9	8.2	8.24	30.39	12.39389	12.39389	gi|1594770	fumarate hydratase [Chlamydomonas rei	0	0
9	8.2	8.24	30.39	12.39389	12.39389	gi|1594770	fumarate hydratase [Chlamydomonas rei	0	0
9	8.2	8.24	30.39	12.39389	12.39389	gi|1594770	fumarate hydratase [Chlamydomonas rei	0	0
9	8.2	8.24	30.39	12.39389	12.39389	gi|1594770	fumarate hydratase [Chlamydomonas rei	0	0
9	8.2	8.24	30.39	12.39389	12.39389	gi|1594770	fumarate hydratase [Chlamydomonas rei	0	0
9	8.2	8.24	30.39	12.39389	12.39389	gi|1594770	fumarate hydratase [Chlamydomonas rei	0	0
9	8.2	8.24	30.39	12.39389	12.39389	gi|1594770	fumarate hydratase [Chlamydomonas rei	0	0
9	8.2	8.24	30.39	12.39389	12.39389	gi|1594770	fumarate hydratase [Chlamydomonas rei	0	0
10	8.1	8.08	26.15	13.17992	13.17992	gi|1594798	UDP-glucose dehydrogenase [Chlamydon	2	99
10	8.1	8.08	26.15	13.17992	13.17992	gi|1594798	UDP-glucose dehydrogenase [Chlamydon	2	99
10	8.1	8.08	26.15	13.17992	13.17992	gi|1594798	UDP-glucose dehydrogenase [Chlamydon	2	99
10	8.1	8.08	26.15	13.17992	13.17992	gi|1594798	UDP-glucose dehydrogenase [Chlamydon	1.7	98
10	8.1	8.08	26.15	13.17992	13.17992	gi|1594798	UDP-glucose dehydrogenase [Chlamydon	0.2	38
10	8.1	8.08	26.15	13.17992	13.17992	gi|1594798	UDP-glucose dehydrogenase [Chlamydon	0.1	22
10	8.1	8.08	26.15	13.17992	13.17992	gi|1594798	UDP-glucose dehydrogenase [Chlamydon	0.1	13.6
10	8.1	8.08	26.15	13.17992	13.17992	gi|1594798	UDP-glucose dehydrogenase [Chlamydon	0	0
10	8.1	8.08	26.15	13.17992	13.17992	gi|1594798	UDP-glucose dehydrogenase [Chlamydon	0	0
10	8.1	8.08	26.15	13.17992	13.17992	gi|1594798	UDP-glucose dehydrogenase [Chlamydon	0	0
10	8.1	8.08	26.15	13.17992	13.17992	gi|1594798	UDP-glucose dehydrogenase [Chlamydon	0	0
11	8	8	15.01	11.56187	11.56187	gi|1594773	catalase/peroxidase [Chlamydomonas rei	2	99
11	8	8	15.01	11.56187	11.56187	gi|1594773	catalase/peroxidase [Chlamydomonas rei	2	99
11	8	8	15.01	11.56187	11.56187	gi|1594773	catalase/peroxidase [Chlamydomonas rei	2	99
11	8	8	15.01	11.56187	11.56187	gi|1594773	catalase/peroxidase [Chlamydomonas rei	2	99
11	8	8	15.01	11.56187	11.56187	gi|1594773	catalase/peroxidase [Chlamydomonas rei	0	0
11	8	8	15.01	11.56187	11.56187	gi|1594773	catalase/peroxidase [Chlamydomonas rei	0	0
12	7.3	7.25	10.88	8.843537	5.442177	gi|4117904	photosystem I P700 chlorophyll a apopro	2	99
12	7.3	7.25	10.88	8.843537	5.442177	gi|4117904	photosystem I P700 chlorophyll a apopro	2	99
12	7.3	7.25	10.88	8.843537	5.442177	gi|4117904	photosystem I P700 chlorophyll a apopro	2	99
12	7.3	7.25	10.88	8.843537	5.442177	gi|4117904	photosystem I P700 chlorophyll a apopro	0.8	83
12	7.3	7.25	10.88	8.843537	5.442177	gi|4117904	photosystem I P700 chlorophyll a apopro	0.5	66
12	7.3	7.25	10.88	8.843537	5.442177	gi|4117904	photosystem I P700 chlorophyll a apopro	0	2.92
12	7.3	7.25	10.88	8.843537	5.442177	gi|4117904	photosystem I P700 chlorophyll a apopro	0	0
12	7.3	7.25	10.88	8.843537	5.442177	gi|4117904	photosystem I P700 chlorophyll a apopro	0	0
13	7.1	7.14	26.84	23.37662	23.37662	gi|136429	Trypsin precursor cRAP	2	99
13	7.1	7.14	26.84	23.37662	23.37662	gi|136429	Trypsin precursor cRAP	2	99
13	7.1	7.14	26.84	23.37662	23.37662	gi|136429	Trypsin precursor cRAP	2	99
13	7.1	7.14	26.84	23.37662	23.37662	gi|136429	Trypsin precursor cRAP	1	90

13	7.1	7.14	26.84	23.37662	23.37662	gi|136429	Trypsin precursor cRAP	0.1	28
13	7.1	7.14	26.84	23.37662	23.37662	gi|136429	Trypsin precursor cRAP	0	98
13	7.1	7.14	26.84	23.37662	23.37662	gi|136429	Trypsin precursor cRAP	0	24
13	7.1	7.14	26.84	23.37662	23.37662	gi|136429	Trypsin precursor cRAP	0	93
13	7.1	7.14	26.84	23.37662	23.37662	gi|136429	Trypsin precursor cRAP	0	64
13	7.1	7.14	26.84	23.37662	23.37662	gi|136429	Trypsin precursor cRAP	0	33
13	7.1	7.14	26.84	23.37662	23.37662	gi|136429	Trypsin precursor cRAP	0	0
13	7.1	7.14	26.84	23.37662	23.37662	gi|136429	Trypsin precursor cRAP	0	99
13	7.1	7.14	26.84	23.37662	23.37662	gi|136429	Trypsin precursor cRAP	0	99
13	7.1	7.14	26.84	23.37662	23.37662	gi|136429	Trypsin precursor cRAP	0	99
13	7.1	7.14	26.84	23.37662	23.37662	gi|136429	Trypsin precursor cRAP	0	99
13	7.1	7.14	26.84	23.37662	23.37662	gi|136429	Trypsin precursor cRAP	0	83
13	7.1	7.14	26.84	23.37662	23.37662	gi|136429	Trypsin precursor cRAP	0	82
13	7.1	7.14	26.84	23.37662	23.37662	gi|136429	Trypsin precursor cRAP	0	15
13	7.1	7.14	26.84	23.37662	23.37662	gi|136429	Trypsin precursor cRAP	0	11
13	7.1	7.14	26.84	23.37662	23.37662	gi|136429	Trypsin precursor cRAP	0	4
13	7.1	7.14	26.84	23.37662	23.37662	gi|136429	Trypsin precursor cRAP	0	3
13	7.1	7.14	26.84	23.37662	23.37662	gi|136429	Trypsin precursor cRAP	0	1
13	7.1	7.14	26.84	23.37662	23.37662	gi|136429	Trypsin precursor cRAP	0	0
13	7.1	7.14	26.84	23.37662	23.37662	gi|136429	Trypsin precursor cRAP	0	0
13	7.1	7.14	26.84	23.37662	23.37662	gi|136429	Trypsin precursor cRAP	0	56
13	7.1	7.14	26.84	23.37662	23.37662	gi|136429	Trypsin precursor cRAP	0	0
14	6.9	6.91	29.61	9.929078	9.929078	gi|1594652	phytoene desaturase [Chlamydomonas re	2	99
14	6.9	6.91	29.61	9.929078	9.929078	gi|1594652	phytoene desaturase [Chlamydomonas re	2	99
14	6.9	6.91	29.61	9.929078	9.929078	gi|1594652	phytoene desaturase [Chlamydomonas re	1.4	96
14	6.9	6.91	29.61	9.929078	9.929078	gi|1594652	phytoene desaturase [Chlamydomonas re	1.3	95
14	6.9	6.91	29.61	9.929078	9.929078	gi|1594652	phytoene desaturase [Chlamydomonas re	0.2	39
14	6.9	6.91	29.61	9.929078	9.929078	gi|1594652	phytoene desaturase [Chlamydomonas re	0	0
14	6.9	6.91	29.61	9.929078	9.929078	gi|1594652	phytoene desaturase [Chlamydomonas re	0	0
14	6.9	6.91	29.61	9.929078	9.929078	gi|1594652	phytoene desaturase [Chlamydomonas re	0	0
14	6.9	6.91	29.61	9.929078	9.929078	gi|1594652	phytoene desaturase [Chlamydomonas re	0	0
14	6.9	6.91	29.61	9.929078	9.929078	gi|1594652	phytoene desaturase [Chlamydomonas re	0	0
14	6.9	6.91	29.61	9.929078	9.929078	gi|1594652	phytoene desaturase [Chlamydomonas re	0	0
14	6.9	6.91	29.61	9.929078	9.929078	gi|1594652	phytoene desaturase [Chlamydomonas re	0	0
14	6.9	6.91	29.61	9.929078	9.929078	gi|1594652	phytoene desaturase [Chlamydomonas re	0	0
14	6.9	6.91	29.61	9.929078	9.929078	gi|1594652	phytoene desaturase [Chlamydomonas re	0	0
14	6.9	6.91	29.61	9.929078	9.929078	gi|1594652	phytoene desaturase [Chlamydomonas re	0	0
14	6.9	6.91	29.61	9.929078	9.929078	gi|1594652	phytoene desaturase [Chlamydomonas re	0	0
14	6.9	6.91	29.61	9.929078	9.929078	gi|1594652	phytoene desaturase [Chlamydomonas re	0	0
14	6.9	6.91	29.61	9.929078	9.929078	gi|1594652	phytoene desaturase [Chlamydomonas re	0	0
15	6.2	6.21	14.25	4.660453	4.660453	gi|4117907	photosystem I P700 chlorophyll a apopro	2	99
15	6.2	6.21	14.25	4.660453	4.660453	gi|4117907	photosystem I P700 chlorophyll a apopro	2	99
15	6.2	6.21	14.25	4.660453	4.660453	gi|4117907	photosystem I P700 chlorophyll a apopro	2	99
15	6.2	6.21	14.25	4.660453	4.660453	gi|4117907	photosystem I P700 chlorophyll a apopro	0.2	39
15	6.2	6.21	14.25	4.660453	4.660453	gi|4117907	photosystem I P700 chlorophyll a apopro	0	99
15	6.2	6.21	14.25	4.660453	4.660453	gi|4117907	photosystem I P700 chlorophyll a apopro	0	12
15	6.2	6.21	14.25	4.660453	4.660453	gi|4117907	photosystem I P700 chlorophyll a apopro	0	4

15	6.2	6.21	14.25	4.660453	4.660453	gi|4117907	photosystem I P700 chlorophyll a apopro	0	0
15	6.2	6.21	14.25	4.660453	4.660453	gi|4117907	photosystem I P700 chlorophyll a apopro	0	0
15	6.2	6.21	14.25	4.660453	4.660453	gi|4117907	photosystem I P700 chlorophyll a apopro	0	0
15	6.2	6.21	14.25	4.660453	4.660453	gi|4117907	photosystem I P700 chlorophyll a apopro	0	0
15	6.2	6.21	14.25	4.660453	4.660453	gi|4117907	photosystem I P700 chlorophyll a apopro	0	0
15	6.2	6.21	14.25	4.660453	4.660453	gi|4117907	photosystem I P700 chlorophyll a apopro	0	0
15	6.2	6.21	14.25	4.660453	4.660453	gi|4117907	photosystem I P700 chlorophyll a apopro	0	0
16	6.1	6.14	31.28	7.821229	7.821229	gi|1594873	flagellar associated protein [Chlamydomo	2	99
16	6.1	6.14	31.28	7.821229	7.821229	gi|1594873	flagellar associated protein [Chlamydomo	2	99
16	6.1	6.14	31.28	7.821229	7.821229	gi|1594873	flagellar associated protein [Chlamydomo	2	99
16	6.1	6.14	31.28	7.821229	7.821229	gi|1594873	flagellar associated protein [Chlamydomo	0.1	11
16	6.1	6.14	31.28	7.821229	7.821229	gi|1594873	flagellar associated protein [Chlamydomo	0	7
16	6.1	6.14	31.28	7.821229	7.821229	gi|1594873	flagellar associated protein [Chlamydomo	0	6.9
16	6.1	6.14	31.28	7.821229	7.821229	gi|1594873	flagellar associated protein [Chlamydomo	0	4.96
16	6.1	6.14	31.28	7.821229	7.821229	gi|1594873	flagellar associated protein [Chlamydomo	0	0.93
16	6.1	6.14	31.28	7.821229	7.821229	gi|1594873	flagellar associated protein [Chlamydomo	0	0
16	6.1	6.14	31.28	7.821229	7.821229	gi|1594873	flagellar associated protein [Chlamydomo	0	0
16	6.1	6.14	31.28	7.821229	7.821229	gi|1594873	flagellar associated protein [Chlamydomo	0	0
16	6.1	6.14	31.28	7.821229	7.821229	gi|1594873	flagellar associated protein [Chlamydomo	0	0
16	6.1	6.14	31.28	7.821229	7.821229	gi|1594873	flagellar associated protein [Chlamydomo	0	3
16	6.1	6.14	31.28	7.821229	7.821229	gi|1594873	flagellar associated protein [Chlamydomo	0	0
16	6.1	6.14	31.28	7.821229	7.821229	gi|1594873	flagellar associated protein [Chlamydomo	0	0
16	6.1	6.14	31.28	7.821229	7.821229	gi|1594873	flagellar associated protein [Chlamydomo	0	0
16	6.1	6.14	31.28	7.821229	7.821229	gi|1594873	flagellar associated protein [Chlamydomo	0	0
17	5.2	5.15	31.94	8.333334	6.746032	gi|1594876	vacuolar ATP synthase subunit B [Chlamy	2	99
17	5.2	5.15	31.94	8.333334	6.746032	gi|1594876	vacuolar ATP synthase subunit B [Chlamy	2	99
17	5.2	5.15	31.94	8.333334	6.746032	gi|1594876	vacuolar ATP synthase subunit B [Chlamy	1.2	93
17	5.2	5.15	31.94	8.333334	6.746032	gi|1594876	vacuolar ATP synthase subunit B [Chlamy	0	0
17	5.2	5.15	31.94	8.333334	6.746032	gi|1594876	vacuolar ATP synthase subunit B [Chlamy	0	0
17	5.2	5.15	31.94	8.333334	6.746032	gi|1594876	vacuolar ATP synthase subunit B [Chlamy	0	0
17	5.2	5.15	31.94	8.333334	6.746032	gi|1594876	vacuolar ATP synthase subunit B [Chlamy	0	0
17	5.2	5.15	31.94	8.333334	6.746032	gi|1594876	vacuolar ATP synthase subunit B [Chlamy	0	0
17	5.2	5.15	31.94	8.333334	6.746032	gi|1594876	vacuolar ATP synthase subunit B [Chlamy	0	0
17	5.2	5.15	31.94	8.333334	6.746032	gi|1594876	vacuolar ATP synthase subunit B [Chlamy	0	0
17	5.2	5.15	31.94	8.333334	6.746032	gi|1594876	vacuolar ATP synthase subunit B [Chlamy	0	0
18	4	4	29.23	29.23077	29.23077	gi|1594897	predicted protein [Chlamydomonas reinh	2	99
18	4	4	29.23	29.23077	29.23077	gi|1594897	predicted protein [Chlamydomonas reinh	2	99
19	3.4	3.38	26.43	6.904762	6.904762	gi|1594628	calreticulin 2, calcium-binding protein [Ch	2	99
19	3.4	3.38	26.43	6.904762	6.904762	gi|1594628	calreticulin 2, calcium-binding protein [Ch	1.3	95
19	3.4	3.38	26.43	6.904762	6.904762	gi|1594628	calreticulin 2, calcium-binding protein [Ch	0.1	16.9
19	3.4	3.38	26.43	6.904762	6.904762	gi|1594628	calreticulin 2, calcium-binding protein [Ch	0	0
19	3.4	3.38	26.43	6.904762	6.904762	gi|1594628	calreticulin 2, calcium-binding protein [Ch	0	0
20	3.1	3.12	26.13	6.479482	4.103672	gi|1594887	eukaryotic translation elongation factor 1	2	99
20	3.1	3.12	26.13	6.479482	4.103672	gi|1594887	eukaryotic translation elongation factor 1	0.9	87
20	3.1	3.12	26.13	6.479482	4.103672	gi|1594887	eukaryotic translation elongation factor 1	0.2	41
20	3.1	3.12	26.13	6.479482	4.103672	gi|1594887	eukaryotic translation elongation factor 1	0	0
20	3.1	3.12	26.13	6.479482	4.103672	gi|1594887	eukaryotic translation elongation factor 1	0	0

20	3.1	3.12	26.13	6.479482	4.103672	gi|1594887	eukaryotic translation elongation factor 1	0	0
20	3.1	3.12	26.13	6.479482	4.103672	gi|1594887	eukaryotic translation elongation factor 1	0	0
20	3.1	3.12	26.13	6.479482	4.103672	gi|1594887	eukaryotic translation elongation factor 1	0	0
20	3.1	3.12	26.13	6.479482	4.103672	gi|1594887	eukaryotic translation elongation factor 1	0	0
20	3.1	3.12	26.13	6.479482	4.103672	gi|1594887	eukaryotic translation elongation factor 1	0	0
20	3.1	3.12	26.13	6.479482	4.103672	gi|1594887	eukaryotic translation elongation factor 1	0	0
21	3	2.96	22.62	5.357143	2.97619	gi|1594706	ADP-glucose pyrophosphorylase large sul	2	99
21	3	2.96	22.62	5.357143	2.97619	gi|1594706	ADP-glucose pyrophosphorylase large sul	1	89
21	3	2.96	22.62	5.357143	2.97619	gi|1594706	ADP-glucose pyrophosphorylase large sul	0	0
21	3	2.96	22.62	5.357143	2.97619	gi|1594706	ADP-glucose pyrophosphorylase large sul	0	0
21	3	2.96	22.62	5.357143	2.97619	gi|1594706	ADP-glucose pyrophosphorylase large sul	0	0
21	3	2.96	22.62	5.357143	2.97619	gi|1594706	ADP-glucose pyrophosphorylase large sul	0	0
21	3	2.96	22.62	5.357143	2.97619	gi|1594706	ADP-glucose pyrophosphorylase large sul	0	0
22	2.6	2.63	12.86	5.099778	3.104213	gi|1594902	alpha tubulin 2 [Chlamydomonas reinhar	2	99
22	2.6	2.63	12.86	5.099778	3.104213	gi|1594902	alpha tubulin 2 [Chlamydomonas reinhar	0.4	59
22	2.6	2.63	12.86	5.099778	3.104213	gi|1594902	alpha tubulin 2 [Chlamydomonas reinhar	0.2	33
22	2.6	2.63	12.86	5.099778	3.104213	gi|1594902	alpha tubulin 2 [Chlamydomonas reinhar	0.1	15
22	2.6	2.63	12.86	5.099778	3.104213	gi|1594902	alpha tubulin 2 [Chlamydomonas reinhar	0	0
23	2.3	2.28	15.52	8.62069	0	gi|1594684	glutamyl-trna reductase [Chlamydomona	1.2	94
23	2.3	2.28	15.52	8.62069	0	gi|1594684	glutamyl-trna reductase [Chlamydomona	1	90
23	2.3	2.28	15.52	8.62069	0	gi|1594684	glutamyl-trna reductase [Chlamydomona	0.1	12
23	2.3	2.28	15.52	8.62069	0	gi|1594684	glutamyl-trna reductase [Chlamydomona	0	0
23	2.3	2.28	15.52	8.62069	0	gi|1594684	glutamyl-trna reductase [Chlamydomona	0	0
23	2.3	2.28	15.52	8.62069	0	gi|1594684	glutamyl-trna reductase [Chlamydomona	0	0
23	2.3	2.28	15.52	8.62069	0	gi|1594684	glutamyl-trna reductase [Chlamydomona	0	0
24	2.1	2.09	21.95	3.310104	3.310104	gi|1594633	dihydrolipoamide dehydrogenase [Chlam	2	99
24	2.1	2.09	21.95	3.310104	3.310104	gi|1594633	dihydrolipoamide dehydrogenase [Chlam	0.1	19
24	2.1	2.09	21.95	3.310104	3.310104	gi|1594633	dihydrolipoamide dehydrogenase [Chlam	0	0
24	2.1	2.09	21.95	3.310104	3.310104	gi|1594633	dihydrolipoamide dehydrogenase [Chlam	0	0
24	2.1	2.09	21.95	3.310104	3.310104	gi|1594633	dihydrolipoamide dehydrogenase [Chlam	0	0
24	2.1	2.09	21.95	3.310104	3.310104	gi|1594633	dihydrolipoamide dehydrogenase [Chlam	0	0
24	2.1	2.09	21.95	3.310104	3.310104	gi|1594633	dihydrolipoamide dehydrogenase [Chlam	0	0
24	2.1	2.09	21.95	3.310104	3.310104	gi|1594633	dihydrolipoamide dehydrogenase [Chlam	0	0
24	2.1	2.09	21.95	3.310104	3.310104	gi|1594633	dihydrolipoamide dehydrogenase [Chlam	0	0
25	2	2	30.38	3.653846	3.653846	gi|1594868	serine hydroxymethyltransferase [Chlamy	2	99
25	2	2	30.38	3.653846	3.653846	gi|1594868	serine hydroxymethyltransferase [Chlamy	0	0
25	2	2	30.38	3.653846	3.653846	gi|1594868	serine hydroxymethyltransferase [Chlamy	0	0
25	2	2	30.38	3.653846	3.653846	gi|1594868	serine hydroxymethyltransferase [Chlamy	0	0
25	2	2	30.38	3.653846	3.653846	gi|1594868	serine hydroxymethyltransferase [Chlamy	0	0
25	2	2	30.38	3.653846	3.653846	gi|1594868	serine hydroxymethyltransferase [Chlamy	0	0
25	2	2	30.38	3.653846	3.653846	gi|1594868	serine hydroxymethyltransferase [Chlamy	0	0
25	2	2	30.38	3.653846	3.653846	gi|1594868	serine hydroxymethyltransferase [Chlamy	0	0
25	2	2	30.38	3.653846	3.653846	gi|1594868	serine hydroxymethyltransferase [Chlamy	0	0
25	2	2	30.38	3.653846	3.653846	gi|1594868	serine hydroxymethyltransferase [Chlamy	0	0
25	2	2	30.38	3.653846	3.653846	gi|1594868	serine hydroxymethyltransferase [Chlamy	0	0
25	2	2	30.38	3.653846	3.653846	gi|1594868	serine hydroxymethyltransferase [Chlamy	0	0
25	2	2	30.38	3.653846	3.653846	gi|1594868	serine hydroxymethyltransferase [Chlamy	0	0

25	2	2	30.38	3.653846	3.653846	gi|1594868	serine hydroxymethyltransferase [Chlamy	0	0
26	2	2	25.48	1.906412	1.906412	gi|1594686	chaperonin 60B2 [Chlamydomonas reinh	2	99
26	2	2	25.48	1.906412	1.906412	gi|1594686	chaperonin 60B2 [Chlamydomonas reinh	0	0
26	2	2	25.48	1.906412	1.906412	gi|1594686	chaperonin 60B2 [Chlamydomonas reinh	0	0
26	2	2	25.48	1.906412	1.906412	gi|1594686	chaperonin 60B2 [Chlamydomonas reinh	0	0
26	2	2	25.48	1.906412	1.906412	gi|1594686	chaperonin 60B2 [Chlamydomonas reinh	0	0
26	2	2	25.48	1.906412	1.906412	gi|1594686	chaperonin 60B2 [Chlamydomonas reinh	0	0
26	2	2	25.48	1.906412	1.906412	gi|1594686	chaperonin 60B2 [Chlamydomonas reinh	0	0
26	2	2	25.48	1.906412	1.906412	gi|1594686	chaperonin 60B2 [Chlamydomonas reinh	0	0
26	2	2	25.48	1.906412	1.906412	gi|1594686	chaperonin 60B2 [Chlamydomonas reinh	0	0
26	2	2	25.48	1.906412	1.906412	gi|1594686	chaperonin 60B2 [Chlamydomonas reinh	0	0
27	2	2	17.9	3.11284	3.11284	gi|1594673	ADP-glucose pyrophosphorylase small su	2	99
27	2	2	17.9	3.11284	3.11284	gi|1594673	ADP-glucose pyrophosphorylase small su	0	0
27	2	2	17.9	3.11284	3.11284	gi|1594673	ADP-glucose pyrophosphorylase small su	0	0
27	2	2	17.9	3.11284	3.11284	gi|1594673	ADP-glucose pyrophosphorylase small su	0	0
27	2	2	17.9	3.11284	3.11284	gi|1594673	ADP-glucose pyrophosphorylase small su	0	0
27	2	2	17.9	3.11284	3.11284	gi|1594673	ADP-glucose pyrophosphorylase small su	0	0
27	2	2	17.9	3.11284	3.11284	gi|1594673	ADP-glucose pyrophosphorylase small su	0	0
28	2	2	19.19	3.783784	3.783784	gi|1594716	predicted protein [Chlamydomonas reinh	2	99
28	2	2	19.19	3.783784	3.783784	gi|1594716	predicted protein [Chlamydomonas reinh	0	0
28	2	2	19.19	3.783784	3.783784	gi|1594716	predicted protein [Chlamydomonas reinh	0	0
28	2	2	19.19	3.783784	3.783784	gi|1594716	predicted protein [Chlamydomonas reinh	0	0
28	2	2	19.19	3.783784	3.783784	gi|1594716	predicted protein [Chlamydomonas reinh	0	0
29	1.3	1.3	7.143	2.813853	2.813853	gi|1594697	predicted protein [Chlamydomonas reinh	1.3	95
29	1.3	1.3	7.143	2.813853	2.813853	gi|1594697	predicted protein [Chlamydomonas reinh	0	0
29	1.3	1.3	7.143	2.813853	2.813853	gi|1594697	predicted protein [Chlamydomonas reinh	0	0
29	1.3	1.3	7.143	2.813853	2.813853	gi|1594697	predicted protein [Chlamydomonas reinh	0	0
29	1.3	1.3	7.143	2.813853	2.813853	gi|1594697	predicted protein [Chlamydomonas reinh	0	0
30	1.2	1.23	8.071	2.362205	0	gi|4117903	photosystem II 47 kDa protein [Chlamydo	1	89
30	1.2	1.23	8.071	2.362205	0	gi|4117903	photosystem II 47 kDa protein [Chlamydo	0.3	46
30	1.2	1.23	8.071	2.362205	0	gi|4117903	photosystem II 47 kDa protein [Chlamydo	0	0
30	1.2	1.23	8.071	2.362205	0	gi|4117903	photosystem II 47 kDa protein [Chlamydo	0	0
31	0.8	0.77	18.45	2.30608	0	gi|1594907	selenium binding protein [Chlamydomon	0.8	83
31	0.8	0.77	18.45	2.30608	0	gi|1594907	selenium binding protein [Chlamydomon	0	0
31	0.8	0.77	18.45	2.30608	0	gi|1594907	selenium binding protein [Chlamydomon	0	0
31	0.8	0.77	18.45	2.30608	0	gi|1594907	selenium binding protein [Chlamydomon	0	0
31	0.8	0.77	18.45	2.30608	0	gi|1594907	selenium binding protein [Chlamydomon	0	0
32	0.7	0.72	15.38	3.846154	0	gi|1594771	S-Adenosylmethionine synthetase [Chlam	0.7	81
32	0.7	0.72	15.38	3.846154	0	gi|1594771	S-Adenosylmethionine synthetase [Chlam	0	0
32	0.7	0.72	15.38	3.846154	0	gi|1594771	S-Adenosylmethionine synthetase [Chlam	0	0
32	0.7	0.72	15.38	3.846154	0	gi|1594771	S-Adenosylmethionine synthetase [Chlam	0	0
32	0.7	0.72	15.38	3.846154	0	gi|1594771	S-Adenosylmethionine synthetase [Chlam	0	0
33	0.6	0.64	34.17	2.68714	0	gi|1594808	alanine aminotransferase [Chlamydomor	0.6	77
33	0.6	0.64	34.17	2.68714	0	gi|1594808	alanine aminotransferase [Chlamydomor	0	0
33	0.6	0.64	34.17	2.68714	0	gi|1594808	alanine aminotransferase [Chlamydomor	0	0
33	0.6	0.64	34.17	2.68714	0	gi|1594808	alanine aminotransferase [Chlamydomor	0	0
33	0.6	0.64	34.17	2.68714	0	gi|1594808	alanine aminotransferase [Chlamydomor	0	0

33	0.6	0.64	34.17	2.68714	0	gi|1594808	alanine aminotransferase [Chlamydomon	0	0
33	0.6	0.64	34.17	2.68714	0	gi|1594808	alanine aminotransferase [Chlamydomon	0	0
33	0.6	0.64	34.17	2.68714	0	gi|1594808	alanine aminotransferase [Chlamydomon	0	0
33	0.6	0.64	34.17	2.68714	0	gi|1594808	alanine aminotransferase [Chlamydomon	0	0
33	0.6	0.64	34.17	2.68714	0	gi|1594808	alanine aminotransferase [Chlamydomon	0	0
33	0.6	0.64	34.17	2.68714	0	gi|1594808	alanine aminotransferase [Chlamydomon	0	0
34	0.5	0.5	11.93	0	0	gi|4117906	photosystem II protein D2 [Chlamydomo	0.3	47
34	0.5	0.5	11.93	0	0	gi|4117906	photosystem II protein D2 [Chlamydomo	0.2	41
34	0.5	0.5	11.93	0	0	gi|4117906	photosystem II protein D2 [Chlamydomo	0	0
34	0.5	0.5	11.93	0	0	gi|4117906	photosystem II protein D2 [Chlamydomo	0	0
34	0.5	0.5	11.93	0	0	gi|4117906	photosystem II protein D2 [Chlamydomo	0	0
35	0.5	0.47	27.17	1.968504	0	gi|1594697	pyruvate kinase [Chlamydomonas reinha	0.5	66
35	0.5	0.47	27.17	1.968504	0	gi|1594697	pyruvate kinase [Chlamydomonas reinha	0	0
35	0.5	0.47	27.17	1.968504	0	gi|1594697	pyruvate kinase [Chlamydomonas reinha	0	0
35	0.5	0.47	27.17	1.968504	0	gi|1594697	pyruvate kinase [Chlamydomonas reinha	0	0
35	0.5	0.47	27.17	1.968504	0	gi|1594697	pyruvate kinase [Chlamydomonas reinha	0	0
35	0.5	0.47	27.17	1.968504	0	gi|1594697	pyruvate kinase [Chlamydomonas reinha	0	0
35	0.5	0.47	27.17	1.968504	0	gi|1594697	pyruvate kinase [Chlamydomonas reinha	0	0
35	0.5	0.47	27.17	1.968504	0	gi|1594697	pyruvate kinase [Chlamydomonas reinha	0	0
35	0.5	0.47	27.17	1.968504	0	gi|1594697	pyruvate kinase [Chlamydomonas reinha	0	0
35	0.5	0.47	27.17	1.968504	0	gi|1594697	pyruvate kinase [Chlamydomonas reinha	0	0

Sequence	Mc	Cleava	dMas	Prec M	Prec m	Theor	Theo	Theo	Sc	Spectrum	Time
FPGQLNADLR			-1	1129	565	1130	566	2	13	1.1.1.810.5	29.1462
GHYTEGAELIDSVLDVVR			0.01	1972	658	1972	658	3	21	1.1.1.965.3	54.9247
GHYTEGAELIDSVLDVVRK		missed	-2	2098	700	2100	701	3	18	1.1.1.925.4	52.1874
INVYFNEATGGR			-0	1340	671	1340	671	2	14	1.1.1.813.2	29.6882
LAVNLIPFPR			0	1139	570	1139	570	2	14	1.1.1.882.3	45.0549
LHFFMVGFTPLTSR			0.02	1652	552	1652	552	3	16	1.1.1.912.2	49.9313
SGPYGQIFRPDNFVFGQTGAGNNWAK			0.01	2827	943	2827	943	3	22	1.1.1.893.2	47.5633
LHFFMVGFTPLTSR	Oxidation(		-0	1668	557	1668	557	3	15	1.1.1.885.2	45.702
VSEQFTAMFR	Oxidation(		0.01	1231	616	1231	616	2	12	1.1.1.808.3	28.5795
YLTASALFR			-0	1041	521	1041	521	2	10	1.1.1.844.3	36.835
EVDEQMLNVQNK	Glu->pyro-		-0	1431	478	1431	478	3	7	1.1.1.864.3	41.2795
GHYTEGAELIDSVLDV	Oxi	cleave	0.04	1733	579	1733	579	3	9	1.1.1.877.3	43.9944
LAVNLIPFPR	Deamidat		0.02	1183	592	1183	592	2	7	1.1.1.1058.	62.5349
NSSYFVEWIPNNVK			-0	1696	849	1696	849	2	5	1.1.1.885.5	45.856
Sequence	Mc	Cleava	dMas	Prec M	Prec m	Theor	Theo	Theo	Sc	Spectrum	Time
CVLLGAVHGIVEALFR	Carbamid		0.01	1753	585	1753	585	3	17	1.1.1.1071.	64.1166
FGPTEEYIVR			0.01	1210	606	1210	606	2	14	1.1.1.815.2	30.152
GMLSVYNSFNEADKK	Oxi	missed	0.02	1718	574	1718	574	3	17	1.1.1.803.2	27.5224
TDGTLGEVFEQISSSDFVILLISDAAQAK			-0	3054	1019	3054	###	3	16	1.1.1.1583.	79.0657
VAIGLRPDSPSWAEAEACGFSK	Methyl(E)		0	2361	788	2361	788	3	21	1.1.1.860.5	40.4526
CVLLGAVHGIVEALFRR	Ca	missed	0.01	1909	478	1909	478	4	12	1.1.1.1031.	60.8319
DINVVLVAPK			-0	1067	534	1067	534	2	13	1.1.1.828.2	33.1177
DSIAEAGMDIK	Oxidation(		0.01	1165	583	1165	583	2	11	1.1.1.783.4	23.1895
CVLLGAVHGIVEALFR	Carbamid		0	1767	590	1767	590	3	12	1.1.1.1060.	63.1024
IDQTYMWK			0	1084	543	1084	543	2	9	1.1.1.804.3	27.7544
QSVESITGPISR			0.01	1273	637	1273	637	2	12	1.1.1.796.4	26.2068
FPMGKIDQTYMWKVGQKVR		missed	0.06	2311	771	2311	771	3	5	1.1.1.1008.	58.3069
GGRDKYPLLKEAFKGIKK	De	missed	-0.1	2048	684	2048	684	3	6	1.1.1.978.2	55.8439
GVAFMVDNCSYTAR	Dethiomet		0.07	1543	772	1543	772	2	9	1.1.1.1070.	63.9356
LGSRKWAPR	Oxi	missed	0.01	1086	544	1086	544	2	7	1.1.1.784.4	23.4214
SRVLSGSRQQAAAK	De	missed	-0	1477	739	1477	739	2	8	1.1.1.868.2	42.1071
SVVQAVQRFDRF	De	cleave	0	1452	485	1452	485	3	6	1.1.1.854.3	39.0573
VSAAVHLDFNTK			-0	1301	651	1301	651	2	6	1.1.1.836.2	34.924
Sequence	Mc	Cleava	dMas	Prec M	Prec m	Theor	Theo	Theo	Sc	Spectrum	Time
ALGQLTPPEIVK			-0	1265	633	1265	633	2	14	1.1.1.835.3	34.7432
AVENINAIIAPALK			0.01	1436	719	1436	719	2	17	1.1.1.866.4	41.796
LTTENICQVVGDDILVTNPVR	Carbamid		0.02	2369	791	2369	791	3	17	1.1.1.913.2	50.1128
SGETEDSFIADLAVGLASGQIK			0.02	2207	737	2207	737	3	19	1.1.1.1031.	60.8833
VNQIGTITESIEAVR			-0	1629	815	1629	815	2	17	1.1.1.866.5	41.8473
EKPEEPLSFMAK			-0	1405	469	1405	469	3	10	1.1.1.815.4	30.2548
AIDAKAVNAL	De	cleave	0	985.5	494	986	494	2	7	1.1.1.789.2	24.4793
AVNALLLK	Deamidat		-0	841.5	422	842	422	2	7	1.1.1.805.2	27.9349

AVNALLLKVNQI	De	cleave	0.01	1297	649	1297	649	2	7	1.1.1.835.4	34.7946
EAVELRDGDKSK	De	cleave	0.03	1328	444	1328	444	3	9	1.1.1.805.4	28.0377
KVEEVLNLCVK	Ca	missed	-0	1330	444	1330	444	3	6	1.1.1.812.4	29.5589
SFMAKALGQLT	De	cleave	-0	1167	584	1167	584	2	6	1.1.1.826.3	32.7043
IEVEDFPAFIVVDDKGNDFFQK		missed	0	2571	858	2571	858	3	17	1.1.1.962.3	54.3809
ITGEGVFLEALER			-0	1433	717	1433	717	2	16	1.1.1.892.3	47.382
VLQVAPEALTLLADSAMR	Oxidation(		0.02	1913	639	1913	639	3	16	1.1.1.936.2	52.6424
VNLNRPMQEVLAQLSSFPIR			0.01	2311	771	2311	771	3	21	1.1.1.1008.	58.3069
FVALELLK			0	931.6	467	932	467	2	10	1.1.1.869.2	42.2883
SLTAFLTDK			-0	994.5	498	995	498	2	9	1.1.1.838.4	35.4913
EALTLLADSAMRDVAH	De	cleave	0.06	1713	572	1713	572	3	9	1.1.1.877.2	43.9431
FVALELLK			-0	931.5	467	932	467	2	6	1.1.1.816.3	30.4354
GLPQYAKDHIIY		cleave	0.04	1417	709	1417	709	2	6	1.1.1.873.5	43.3725
GVFRAYTSTNLRYSQVAPLDMFSEK	Ox	cleave	0.04	3496	1166	3496	###	3	7	1.1.1.1190.	68.5085
IAKGGGSANKTSLY	De	cleave	-0	1383	692	1383	692	2	8	1.1.1.858.4	39.9362
NTGTNLPAQI	De	cleave	-0	1029	515	1029	515	2	7	1.1.1.782.2	22.8547
PTSGNALGRAYRDL	Ox	cleave	-0	1507	754	1507	754	2	8	1.1.1.846.2	37.2486
TVKLASTRYL	Ox	cleave	0	1167	584	1167	584	2	6	1.1.1.816.4	30.4867
AWNSDKLPIYEPGLLEVVQEAR		missed	0.03	2526	843	2526	843	3	22	1.1.1.925.3	52.136
EIGFIVYALGKPLDQFLQK			0.01	2178	727	2178	727	3	16	1.1.1.1027.	60.4155
TLAEVYAHWIPR			0.01	1455	486	1455	486	3	14	1.1.1.870.4	42.6236
LTANAFLAQR			0.01	1104	553	1104	553	2	13	1.1.1.813.5	29.8424
MVKPAFIFDGR			0.01	1280	428	1280	428	3	12	1.1.1.835.5	34.846
ILTANLWSAELAK			-0	1429	715	1429	715	2	10	1.1.1.872.4	43.0886
IAIYGFAFK			0	1029	515	1029	515	2	10	1.1.1.877.4	44.0458
AKLTANAFLAQRISSINAI	De	cleave	-0	2004	669	2004	669	3	8	1.1.1.908.3	49.4108
FEWDRPNYSR			0.02	1369	457	1369	457	3	8	1.1.1.799.5	26.9542
TANAFLAQR	De	cleave	0.03	991.5	497	992	497	2	6	1.1.1.805.3	27.9863
VGGPTMAMVALK	Ox	cleave	0.02	1190	596	1190	596	2	7	1.1.1.806.2	28.1669
APGVQTPVIVR			-0	1136	569	1136	569	2	15	1.1.1.794.3	25.6912
GFFEVTHDISALTAADFLR			0.01	2109	704	2109	704	3	20	1.1.1.997.2	57.4422
GPILLEDYHLVEK			0	1525	509	1525	509	3	16	1.1.1.852.5	38.6953
LGPNYLLLPVNAPR			0.02	1536	769	1536	769	2	14	1.1.1.876.4	43.8133
AFDAARQER	For	missed	0.05	1107	554	1107	554	2	9	1.1.1.781.4	22.7255
MQAGARWRAFDAAR	De	cleave	-0	1623	542	1623	542	3	7	1.1.1.797.3	26.3874
ALYGFDFLLSSK			-0	1360	681	1360	681	2	16	1.1.1.923.2	51.8757
QILIEPVFAQWIQAAHGK			-0	2048	684	2048	684	3	20	1.1.1.978.2	55.8439
TPLANLVYWK			-0	1204	603	1204	603	2	14	1.1.1.872.5	43.14
DFGYSFPCDGPGR	Carbamid		0	1474	738	1474	738	2	12	1.1.1.834.3	34.5108
FSQGLAQDPTTR			0.01	1320	661	1320	661	2	11	1.1.1.778.5	22.0811
FSQGLAQDPTTRR		missed	-0	1476	493	1476	493	3	9	1.1.1.767.3	20.6128
DKPVALSI		cleave	0.02	841.5	422	841	422	2	6	1.1.1.802.4	27.3933
TKLFPKFSQGLAQDPTTR	De	cleave	-0.1	2035	679	2035	679	3	6	1.1.1.890.5	47.0194
IQVRLGEHNIDVLEGNEQFINAAKII	Ox	missed	0.16	6061	1011	6061	###	6	17	1.1.1.1014.	59.0861
LGEHNIDVLEGNEQFINAAKIITHPN	De	missed	0.12	4517	904	4517	904	5	17	1.1.1.997.4	57.545
LGEHNIDVLEGNEQFINAAKIITHPN	For	missed	0.15	5559	927	5559	927	6	23	1.1.1.1016.	59.3974
LSSPATLNSR			-0	1045	523	1045	523	2	11	1.1.1.764.3	20.2784

VATVSLPR			-0	841.5	422	842	422	2	10	1.1.1.785.2	23.5506
IQVRLGEHNIDVLEGNEQFINAAKII	Ox	missed	0.16	6061	1011	6061	###	6	16	1.1.1.1014.	59.1376
IQVRLGEHNIDVLEGNEQFINAAKII	Ca	missed	0.09	6054	1010	6054	###	6	13	1.1.1.1015.	59.2161
LGEHNIDVLEGNEQFINAAKIITHPN	Ox	missed	0.08	4504	902	4504	902	5	15	1.1.1.995.2	57.0796
LGEHNIDVLEGNEQFINAAKIITHPN	Ox	missed	0.08	4504	902	4504	902	5	14	1.1.1.995.3	57.131
LGEHNIDVLEGNEQFINAAKIITHPN	De	missed	0.03	4504	1127	4504	###	4	14	1.1.1.996.4	57.3124
LGEHNIDVLEGNEQFINAAKIITHPN	Ca	missed	0.12	4517	904	4517	904	5	8	1.1.1.997.3	57.4937
LGEHNIDVLEGNEQFINAAKIITHPN	Ox	missed	0.13	5545	925	5545	925	6	20	1.1.1.1016.	59.4489
LGEHNIDVLEGNEQFINAAKIITHPN	Me	missed	0.11	5559	927	5559	927	6	22	1.1.1.1016.	59.5518
LGEHNIDVLEGNEQFINAAKIITHPN	For	missed	0.16	5558	927	5558	927	6	21	1.1.1.1024.	59.9229
LGEHNIDVLEGNEQFINAAKIITHPN	Ox	missed	0.12	5561	928	5561	928	6	19	1.1.1.1030.	60.7021
LGEHNIDVLEGNEQFINAAKIITHPN	Ox	missed	0.12	5561	928	5561	928	6	15	1.1.1.1030.	60.7535
LGEHNIDVLEGNEQFINAAKIITHPN	Ox	missed	0.14	5561	928	5561	928	6	15	1.1.1.1030.	60.6508
LGEHNIDVLEGNEQFINAAKIITHPN	De	missed	0.17	5559	928	5559	927	6	13	1.1.1.1029.	60.521
LGEHNIDVLEGNEQFINAAKIITHPN	Ca	missed	0.04	5575	930	5575	930	6	13	1.1.1.1008.	58.3583
LGEHNIDVLEGNEQFINAAKIITHPN	Ca	missed	0.11	5558	927	5558	927	6	12	1.1.1.1024.	59.9744
LGEHNIDVLEGNEQFINAAKIITHPN	Gly	missed	0.14	5615	937	5615	937	6	12	1.1.1.984.3	56.5219
LGEHNIDVLEGNEQFINAAKIITHPN	De	missed	0.06	5575	930	5575	930	6	12	1.1.1.1008.	58.2555
LGEHNIDVLEGNEQFINAAKIITHPN	Gly	missed	0.11	5617	937	5617	937	6	11	1.1.1.983.2	56.2378
LGEHNIDVLEGNEQFINAAKIITHPN	De	missed	0.09	5617	937	5617	937	6	11	1.1.1.983.3	56.2892
LSSPATLNSR			0.01	1045	523	1045	523	2	11	1.1.1.769.2	20.8187
VATVSLPR			0.01	841.5	422	842	422	2	6	1.1.1.802.4	27.3933
IQFAIGLLPAIIFGQK			-0	1728	865	1728	865	2	15	1.1.1.1162.	66.8431
SDEDIIAATMTELER			0.01	1693	847	1693	847	2	15	1.1.1.924.3	52.0057
LAAEQIVNDYNYK			-0	1540	771	1540	771	2	13	1.1.1.827.4	32.988
KLTTVDHLLFSR		missed	-0	1429	477	1429	477	3	12	1.1.1.827.5	33.0394
NFFLAGDFTK			-0	1159	580	1159	580	2	10	1.1.1.867.4	42.0286
DVLGGKVAAWK		missed	-0	1143	572	1143	572	2	7	1.1.1.830.2	33.5819
DVLGGKVAAWK		missed	-0	1143	572	1143	572	2	6	1.1.1.833.4	34.381
EAFRPSQRTPIK	Glu	missed	-0	1413	472	1413	472	3	6	1.1.1.830.5	33.7362
GRSDEDIIAATMTELERL	Ox	cleave	0.02	2035	679	2035	679	3	7	1.1.1.890.5	47.0194
KLLVPDQWKPNPYFSQLKEL	Ox	cleave	-0	2458	820	2458	820	3	8	1.1.1.1187.	68.3274
KLTTVDHLLFSR		missed	-0	1429	477	1429	477	3	5	1.1.1.825.5	32.5749
KLTTVDHLLFSR		missed	-0	1429	477	1429	477	3	4	1.1.1.860.3	40.3497
LASMEGAIFSGK		cleave	0	1210	606	1210	606	2	8	1.1.1.815.2	30.152
LFPTEIK			-0	846.5	424	846	424	2	7	1.1.1.799.3	26.8515
PSQRTPIK	De	cleave	-0	926.5	464	927	464	2	6	1.1.1.779.5	22.3129
QANLVAKGASCPRVAVR	Gln	missed	-0	1797	600	1797	600	3	7	1.1.1.851.3	38.36
QANLVAKGASCPRVAVRRVAGR	De	missed	-0	2369	791	2369	791	3	8	1.1.1.913.2	50.1128
QEAKALSSKLKDAPR		cleave	0.03	1641	548	1641	548	3	6	1.1.1.1056.	62.2241
AIMADLYPSFAK			-0	1326	664	1326	664	2	16	1.1.1.864.4	41.3308
IAVDRNPVETSFEK		missed	0.01	1604	536	1604	536	3	18	1.1.1.798.5	26.7223
LLDAGVDPK			-0	926.5	464	927	464	2	14	1.1.1.779.5	22.3129
EIPLPHDLLLNR			-0	1429	477	1429	477	3	11	1.1.1.857.2	39.7034
AIMADLYPSFAK	Oxidation(		-0	1342	672	1342	672	2	15	1.1.1.838.5	35.5427
EIPLPHDLLLNR	Glu->pyro-		0.01	1411	471	1411	471	3	10	1.1.1.859.3	40.1172
EIPLPHDLLLNR			-0	1429	477	1429	477	3	9	1.1.1.860.3	40.3497

EIPLPHDLLLNRAIMADLYPSFAK	De	missed	0.04	2737	914	2737	913	3	6	1.1.1.1174.	67.2632
GVLFARSSR		missed	-0	991.5	497	992	497	2	5	1.1.1.805.3	27.9863
ISHLNWVCIFLGFHSFGLY	Ox	cleave	-0	2341	781	2341	781	3	8	1.1.1.979.2	55.9227
LLDAGVDPK			-0	926.5	927	927	928	1	6	1.1.1.1016.	59.5518
NALAATSLTWGGEL		cleave	-0	1403	702	1403	702	2	7	1.1.1.830.3	33.6334
SSRLIPDK	De	missed	0.02	915.5	459	916	459	2	7	1.1.1.784.5	23.4728
TISTPEREAKKVKI	Pro	cleave	-0	1641	821	1641	821	2	9	1.1.1.1061.	63.2321
ALYGLTDAAALTDLK			-0	1535	768	1535	768	2	19	1.1.1.883.4	45.3392
NSAVSPLVAWIYYQPK			-0	1835	613	1835	613	3	15	1.1.1.959.2	54.042
VTGVNVFPTTR			-0	1190	596	1190	596	2	14	1.1.1.806.2	28.1669
QISSSSVQFLQDR			-0	1494	748	1494	748	2	10	1.1.1.822.5	31.8784
ILNVLDR			0.01	841.5	422	842	422	2	9	1.1.1.805.2	27.9349
YVATFFNR			-0	1017	509	1017	509	2	9	1.1.1.819.3	31.08
VVTNLYTFAK			-0	1155	578	1155	578	2	9	1.1.1.826.4	32.7557
EAAANTAFNAIVSAYNANK			0	1939	647	1939	647	3	9	1.1.1.890.4	46.9681
ALYGLTDAAALTDLK			-0.1	1535	513	1535	513	3	6	1.1.1.904.3	48.5825
ASVDVVIDESFYDGKNPTQITLDDFI	Car	cleave	0.13	4504	902	4504	902	5	10	1.1.1.995.2	57.0796
EAAANTAFNAIVSAYNANKQLAATYLSI		missed	-0.1	2914	972	2914	973	3	6	1.1.1.955.2	53.6759
GNNNTITVSGSQLVF	De	cleave	0.03	1553	519	1553	519	3	8	1.1.1.903.2	48.2985
ILNVLDR			0.01	841.5	422	842	422	2	9	1.1.1.802.4	27.3933
LANQRLLR	De	cleave	-0	984.6	493	985	493	2	6	1.1.1.795.4	25.9747
QLAATYLSK	Deamidat		-0	994.5	498	995	498	2	5	1.1.1.838.4	35.4913
TATADPGPLNR	Ox	cleave	0.03	1129	565	1129	565	2	7	1.1.1.777.4	21.798
YVATFFNR	Oxidation(		0	1033	517	1033	517	2	6	1.1.1.860.2	40.2983
IPLFSAAGLPHNDIAAQICR	Carbamid		-0	2163	722	2163	722	3	18	1.1.1.880.4	44.6411
TIFNSLDLAWSLLR			0.01	1648	825	1648	825	2	16	1.1.1.1084.	64.5669
YAEIVEIR			0.01	991.5	497	992	497	2	11	1.1.1.805.3	27.9863
AVDALSSELAFK	Pro	cleave	0	1292	647	1292	647	2	8	1.1.1.902.2	48.0658
EVSAAREEVPGRR		missed	0.05	1455	486	1455	486	3	6	1.1.1.917.3	51.0953
EVSAAREEVPGRRGYPGYMYTDLATIYI		missed	-0.1	3633	909	3633	909	4	6	1.1.1.1010.	58.6693
LDITGASINPAERTYPEEMIQTGISTI	De	cleave	0.04	4120	1031	4120	###	4	6	1.1.1.952.2	53.4911
LGDGSLR			-0	716.4	717	716	717	1	5	1.1.1.892.3	47.382
RGQVLEVDGTR	Ox	missed	-0	1245	416	1245	416	3	6	1.1.1.841.2	36.0862
TVAGVSGPLVVVECVKKPK	Car	missed	-0	1966	493	1966	493	4	5	1.1.1.824.4	32.2915
VDALSSELAFK	Ox	cleave	0.05	1211	606	1211	606	2	7	1.1.1.791.5	25.0977
AKPLVEQLIAITSGTDAGAK			-0	1982	662	1982	662	3	17	1.1.1.908.2	49.3594
ASAAQKEEIAALVTELSR			0.01	1886	630	1886	630	3	19	1.1.1.888.5	46.5543
SGSIFDNILVTDDLEAAKK		missed	-0	2035	679	2035	679	3	14	1.1.1.890.5	47.0194
FVGFELWQVK			0	1252	627	1252	627	2	12	1.1.1.905.4	48.8667
GIWVAPDIDNPDYVHDDKLYNFK		missed	0.01	2733	684	2733	684	4	11	1.1.1.879.3	44.4596
KEEDEKKAKDAP	Ox	cleave	0.01	1403	702	1403	702	2	7	1.1.1.843.2	36.5512
KVHVILTYKGKNYLIKKDIKAETDQLT	Ox	missed	-0.2	5545	925	5545	925	6	8	1.1.1.1016.	59.4489
IQVHTLLDALNSFVVVPER			0.01	2149	717	2149	717	3	22	1.1.1.1010.	58.7206
LLFELGGIPER			-0	1243	622	1243	622	2	12	1.1.1.879.2	44.4082
LINLLGVK			-0	868.6	435	869	435	2	10	1.1.1.845.3	37.0675
DDFVNKSVPILPISGWLGDNLITK	Ox	missed	0.03	2752	918	2752	918	3	9	1.1.1.1118.	65.6258
ETGGKKMENPVGLK	Ox	missed	0.02	1503	752	1503	752	2	7	1.1.1.971.2	55.2417

ETGGKKMENPVGLK		missed	0.01	1487	744	1487	744	2	5	1.1.1.1047.	61.7258
ETGGKKMENPVGLK		missed	0.01	1487	744	1487	744	2	5	1.1.1.1049.	61.9337
GYSPIGFVRCGRSACRISGINWKVG	Ca	missed	-0.1	4395	880	4395	880	5	7	1.1.1.977.2	55.7138
KGEKIQVHTLLDALNSF	De	cleave	0.02	1913	639	1913	639	3	8	1.1.1.936.2	52.6424
MENPVGLK			-0	886.4	444	886	444	2	5	1.1.1.790.3	24.763
VDKAGPGDNVGMNIK	Ox	missed	-0	1530	511	1530	511	3	6	1.1.1.847.3	37.5324
AIEDVLILSGDHLYR			-0	1713	572	1713	572	3	17	1.1.1.877.2	43.9431
AKPAVPIGGAYR			-0	1199	401	1199	401	3	12	1.1.1.780.4	22.4933
EAEGIYIRSGIVVIDKGALVPDNT	Ox	cleave	-0	2544	637	2544	637	4	6	1.1.1.836.5	35.0783
EFGLMKIDEK	Glu	missed	0.01	1143	572	1143	572	2	7	1.1.1.830.2	33.5819
IASMGIYVFKKSVLLQL		cleave	-0	1909	478	1909	478	4	7	1.1.1.1031.	60.8319
NMGSGVRFGGDGFVEVLAATQTP	De	cleave	0.02	2686	896	2686	896	3	8	1.1.1.963.3	54.5107
SWLLEDTKNRAIEDVLI		cleave	-0	2014	672	2014	672	3	6	1.1.1.891.2	47.098
LIAQVISSLTASLR			0.01	1471	736	1471	736	2	16	1.1.1.998.2	57.6235
EIVDLALDR			0	1043	522	1043	522	2	11	1.1.1.827.3	32.9367
SLDIERPTYTNLNR			0.01	1691	565	1691	565	3	11	1.1.1.810.2	28.992
FDGALNVDITEFQTNLVPYPR	Deamidat		-0	2409	804	2409	804	3	11	1.1.1.957.2	53.8847
FDGALNVDITEFQTNLVPYPR			-0	2408	603	2408	603	4	7	1.1.1.804.5	27.8571
TSGVPLDELRPYLFLLR			0.01	1988	664	1988	664	3	13	1.1.1.999.3	57.8048
NIAPAINELEHGIVYNVDDLKEVVAANK			0	3048	763	3048	763	4	13	1.1.1.1000.	57.8832
FVDISVPR			0.01	931.5	467	932	467	2	9	1.1.1.816.3	30.4354
AVEELSKGIVNK		missed	-0	1286	644	1286	644	2	6	1.1.1.836.3	34.9755
KEVVAANKEGR		cleave	0	1200	601	1200	601	2	7	1.1.1.884.5	45.6234
NIAPAINELEHGIVYNVDDLKEVVAANK			-0	3048	763	3048	763	4	5	1.1.1.1063.	63.594
SKAETIRAAEFEK	Ox	missed	-0.1	1495	499	1495	499	3	6	1.1.1.773.4	21.1798
VTPGVPGVKPVVIELTDFK			-0	1994	666	1994	666	3	16	1.1.1.891.3	47.1494
LGVSKTSFKGNSK	Ca	missed	-0	1395	698	1395	698	2	11	1.1.1.1070.	64.0384
ALLAASGRRATAVGR	De	missed	0.06	1471	736	1471	736	2	6	1.1.1.998.2	57.6235
DREIARLAQR	Ox	cleave	0	1243	622	1243	622	2	7	1.1.1.879.2	44.4082
LLINGRPIDYHTGVIASK			0.01	1966	493	1966	493	4	6	1.1.1.824.4	32.2915
MAASMAATQQR			0	1165	583	1165	583	2	5	1.1.1.783.4	23.1895
RGFVPVNEK		missed	0.02	1045	523	1045	523	2	5	1.1.1.845.4	37.1188
SAKANASAKRFNGLVAEAMSSCIP	De	missed	-0	3254	1086	3254	###	3	6	1.1.1.1289.	71.1748
VTPGVPGVK	Ox	cleave	0.07	868.6	435	869	435	2	7	1.1.1.845.3	37.0675
SYQQQVLSNSQALAGALAK			0.02	1976	660	1976	660	3	15	1.1.1.875.3	43.632
ALSSAHAVAGQR			0.05	1167	584	1167	584	2	5	1.1.1.816.4	30.4867
ALSSAHAVAGQR			-0	1167	584	1167	584	2	5	1.1.1.826.3	32.7043
ESTPDINALKKDVETFAMR	Ox	missed	0.01	2180	728	2180	728	3	7	1.1.1.1011.	58.9018
GAMIFYRK	De	missed	0.04	985.5	494	986	494	2	6	1.1.1.817.5	30.7188
INFAVFPGLQGGPHNHTIAGLACAL	De	missed	-0	3593	1199	3593	###	3	7	1.1.1.1009.	58.4368
LADMAHISGLVAADLVPSPF	Ox	cleave	-0	2039	681	2039	681	3	7	1.1.1.948.2	53.2791
MGSPALTSRGFVEK	Ox	missed	-0.1	1495	499	1495	499	3	6	1.1.1.766.2	20.4841
MGSPALTSRGFVEK	Ox	missed	-0	1511	505	1511	505	3	7	1.1.1.885.3	45.7533
MQRTVQGISQLAR		missed	-0	1487	744	1487	744	2	6	1.1.1.1057.	62.3539
SLRGPRGAMIFYR	Ox	missed	-0	1539	514	1539	514	3	8	1.1.1.921.5	51.6156
YYGGNEFIDQAER	Deamidat		0.02	1562	782	1562	782	2	7	1.1.1.871.4	42.8561
YYGGNEFIDQAER	Deamidat		0.01	1562	782	1562	782	2	8	1.1.1.873.3	43.2697

YYGGNEFIDQAER			0.03	1561	781	1561	781	2	6	1.1.1.874.2	43.4508
DIIGILEAAIR			-0	1183	592	1183	592	2	14	1.1.1.1058.	62.5349
ALSYPIKLIANNAGTNGSVVMQR	De	missed	-0	2369	791	2369	791	3	8	1.1.1.913.2	50.1128
EVELEDPVENIGAK	Glu->pyro-		0.05	1539	514	1539	514	3	8	1.1.1.903.3	48.3499
GILEAAIR		cleave	-0	841.5	422	842	422	2	6	1.1.1.785.2	23.5506
IVAAGTNPVQLTRGMEK	De	missed	-0	1802	602	1802	602	3	7	1.1.1.826.5	32.8071
IVAAGTNPVQLTRGMEKTVNALVK	De	missed	0.09	5559	928	5559	927	6	5	1.1.1.1029.	60.521
LATVVGVTIGPK	Oxidation(		-0	1170	586	1170	586	2	8	1.1.1.947.2	53.2004
LNERIAR	Ox	missed	-0	902.4	452	902	452	2	7	1.1.1.788.4	24.35
PIKLIANNAGTNGSVVMQR		cleave	0.03	1982	662	1982	662	3	7	1.1.1.908.2	49.3594
VSISKEATTIVGDGRTQQQVEGRVK		missed	-0	2685	672	2685	672	4	7	1.1.1.891.2	47.098
EGVEDFLILSGDHLYR			0.02	1862	622	1862	622	3	15	1.1.1.910.4	49.6712
DGIVVVIK			-0	841.5	422	842	422	2	6	1.1.1.785.2	23.5506
DGIVVVIK			-0	841.5	422	842	422	2	5	1.1.1.802.4	27.3933
DIPVSNCLNSNVTKIYCLTQF	Ca	cleave	0.01	2486	830	2486	830	3	7	1.1.1.1032.	60.9617
DSHIPAGTII			0.01	1023	512	1023	512	2	8	1.1.1.814.3	29.9715
EASAFGLMK			0.06	952.5	477	952	477	2	7	1.1.1.809.4	28.8628
FLPPSKVMDCDVNMSIIGDGCVIKA	Ca	missed	0.03	3054	1019	3053	###	3	7	1.1.1.1583.	79.0657
TIGGVVITVARPAK			-0	1381	461	1381	461	3	14	1.1.1.807.2	28.2962
IEGAKLNFQTPEDVKAYVESLK	De	cleave	-0	2480	828	2480	828	3	6	1.1.1.1184.	67.6313
KDPMNINRIYIKQLKNSGVVK	De	cleave	-0.1	2458	820	2458	820	3	8	1.1.1.1188.	68.4048
NSGVVKVQALK	De	missed	-0.1	1143	572	1143	572	2	8	1.1.1.830.2	33.5819
VGKATLPAK	Ox	missed	-0	915.5	459	916	459	2	8	1.1.1.784.5	23.4728
TGALVGGVLGAPR			-0	1167	584	1167	584	2	12	1.1.1.816.4	30.4867
EQALHNPLFIESR	Glu	cleave	-0.1	1535	513	1535	513	3	8	1.1.1.830.4	33.6848
RAKLEKR	Ox	missed	-0	915.5	459	916	459	2	9	1.1.1.784.5	23.4728
TGALVGGVLGAPR	Oxidation(		-0	1183	592	1183	592	2	6	1.1.1.946.2	53.1217
TGALVGGVLGAPR	Oxidation(		0.02	1183	592	1183	592	2	5	1.1.1.1058.	62.5349
KAQLGEIFEFDR		missed	0.01	1452	485	1452	485	3	12	1.1.1.854.3	39.0573
LAFYDYIGNNPAK			-0	1485	743	1485	743	2	11	1.1.1.854.4	39.1087
ELFRDPR		cleave	0.04	931.5	467	931	467	2	6	1.1.1.816.3	30.4354
KLGDTSSLR	Ox	missed	0.01	991.5	497	992	497	2	6	1.1.1.805.3	27.9863
LLVLPALGSGR			-0	1095	548	1095	548	2	11	1.1.1.856.4	39.5737
ATPLDAYKNGAREKLLYVPCVVPDH	Ca	cleave	-0.1	3069	768	3069	768	4	9	1.1.1.892.5	47.4848
DSPLKVVSGLPEDTPEQPVLPTVAGVTL		missed	0.01	3014	754	3014	754	4	5	1.1.1.1062.	63.4131
GLLPLEVRFLHE	Ox	cleave	0.05	1438	480	1438	480	3	7	1.1.1.821.3	31.5438
VWVGGLVHK	Oxidation(		0.03	1010	506	1010	506	2	8	1.1.1.817.2	30.5646
FVIGGPHGDAGLTGR			-0	1453	485	1453	485	3	12	1.1.1.802.3	27.3419
GGNKR	Ox	missed	-0	546.3	547	546	547	1	4	1.1.1.781.5	22.7769
GGNKRYQKTAAYGHFGRDDPDFTV	De	missed	-0	3633	909	3633	909	4	7	1.1.1.1010.	58.6693
HLNPSGRFVIGGPHGDAGLTGRK	De	cleave	-0	2375	793	2375	793	3	8	1.1.1.1025.	60.1046
NLDLKRGGNKR	Ox	missed	0	1286	644	1286	644	2	6	1.1.1.836.3	34.9755
LYGGTLVGYFLDER			0.01	1602	802	1602	802	2	12	1.1.1.956.3	53.8061
AGNNGVSTSWAVGGTR	Deamidat		0.04	1535	513	1535	513	3	6	1.1.1.904.3	48.5825
AGNNGVSTSWAVGGTRLKSAMPQPD		cleave	0.1	3495	1166	3495	###	3	7	1.1.1.1185.	67.7608
ENLQELIK			-0	985.5	494	986	494	2	7	1.1.1.817.5	30.7188
GGVGAYTDSRGNPLVR	De	missed	0.01	1620	541	1620	541	3	6	1.1.1.812.5	29.6103

GNPLVREEVARFIEKRDGVPSNPDH	Oxi	missed	0	4076	1020	4076	###	4	8	1.1.1.996.3	57.2609
RGWGLSVEELQRALQEAR	De	missed	0.03	2098	700	2098	700	3	6	1.1.1.925.4	52.1874
RRAHMVTDGFNAL	Oxi	cleave	0.05	1503	752	1503	752	2	7	1.1.1.964.2	54.6919
TNVGNPHALGAKPLTF	Oxi	cleave	0	1652	552	1652	552	3	7	1.1.1.912.2	49.9313
TQYAVRGELYLRAEQLR	Iod	missed	0.01	2192	1097	2192	###	2	8	1.1.1.1059.	62.8187
VEDMFPADAIAR			0.04	1334	668	1334	668	2	5	1.1.1.837.2	35.1562
LVFPEEVLPR			0.01	1198	600	1198	600	2	11	1.1.1.865.3	41.5121
NILLNEGIR			0	1041	521	1041	521	2	10	1.1.1.813.3	29.7396
NILLNEGIR	Deamidate		0	1043	522	1043	522	2	8	1.1.1.827.3	32.9367
TIAIGTYQEKR	De	cleave	0.01	1280	428	1280	428	3	6	1.1.1.835.5	34.846
TRWCQLGGLWAF	Ca	cleave	-0	1495	499	1495	499	3	7	1.1.1.773.4	21.1798
SVEVLEELLR			0.01	1186	594	1186	594	2	11	1.1.1.903.5	48.4527
DDILAKTDSVMVARGDLGMEIPTEI	Oxi	cleave	0.08	2752	918	2752	918	3	10	1.1.1.1118.	65.6258
EAEASLDYYAMFK			0.06	1537	513	1537	513	3	6	1.1.1.856.2	39.4709
KLAQDVKPGSQILCADGSIVLEVVST	De	missed	-0.1	3495	1166	3495	###	3	7	1.1.1.1187.	68.2246
QVLGERGRSIK	De	missed	-0	1243	622	1243	622	2	5	1.1.1.879.2	44.4082
TDSVMVARGDLGMEIPTEKIFLAQKMN		missed	0.04	3380	1128	3380	###	3	6	1.1.1.1187.	68.276
TDSVMVARGDLGMEIPTEKIFLAQK	Ca	missed	0.08	5617	937	5617	937	6	5	1.1.1.983.3	56.2892
VENQEGIQNF	De	cleave	0.02	1179	590	1179	590	2	7	1.1.1.888.2	46.4
VENQEGIQNFDDI	Oxi	cleave	0.1	1553	519	1553	519	3	9	1.1.1.907.2	49.2293
YAMFKNILK	Oxi	cleave	-0	1143	572	1143	572	2	8	1.1.1.830.2	33.5819
