# Supplementary material for: Metabolic labeling with stable isotope nitrogen (15N) to follow amino acid and protein turnover of three plastid proteins in Chlamydomonas reinhardtii
Source: Proteome Sci. 2014 Mar 3;12:14. doi: 10.1186/1477-5956-12-14 (PMC3943399; doi:10.1186/1477-5956-12-14)
Supplement: Additional file 2 — List of proteins with a minimum of two unique peptides with percent confidence levels of 95 and greater. [file 1477-5956-12-14-S2.pdf]

Sequences for various other proteins

N	Unus	Total	%Cov	%Cov(95)	Accessions	Names	Cor	Conf	Sequence	Modificatic	Cleavages
6	15	15	30.69977	20.54176	gi|1594718	beta tubulin 1 [C	2	99	FPGQLNADLR		
6	15	15	30.69977	20.54176	gi|1594718	beta tubulin 1 [C	2	99	GHYTEGAELIDSVLDVVR		
6	15	15	30.69977	20.54176	gi|1594718	beta tubulin 1 [C	2	99	GHYTEGAELIDSVLDVVI		missed R-K
6	15	15	30.69977	20.54176	gi|1594718	beta tubulin 1 [C	2	99	INVYFNEATGGR		
6	15	15	30.69977	20.54176	gi|1594718	beta tubulin 1 [C	2	99	LAVNLIPFPR		
6	15	15	30.69977	20.54176	gi|1594718	beta tubulin 1 [C	2	99	LHFFMVGFTPLTSR		
6	15	15	30.69977	20.54176	gi|1594718	beta tubulin 1 [C	2	99	SGPYGQIFRPDNFVFGQTGAGNNW		
6	15	15	30.69977	20.54176	gi|1594718	beta tubulin 1 [C	0	99	LHFFMVGF	Oxidation(M)@5	
N	Unus	Total	%Cov	%Cov(95)	Accessions	Names	Cor	Conf	Sequence	Modificatic	Cleavages
7	14	14	40.18018	18.55856	gi|1594893	acetohydroxy ac	2	99	CVLLGAVH	Carbamidomethyl(C)@	
7	14	14	40.18018	18.55856	gi|1594893	acetohydroxy ac	2	99	FGPTEEYIVR		
7	14	14	40.18018	18.55856	gi|1594893	acetohydroxy ac	2	99	GMLSVYNS	Oxidation(	missed K-K
7	14	14	40.18018	18.55856	gi|1594893	acetohydroxy ac	2	99	TDGTLGEVFEQISSSDFVILLISDAAQ		
7	14	14	40.18018	18.55856	gi|1594893	acetohydroxy ac	2	99	VAIGLRPDS	Methyl(E)@16; Carban	
7	14	14	40.18018	18.55856	gi|1594893	acetohydroxy ac	2	97	CVLLGAVH	Carbamido	missed R-R
7	14	14	40.18018	18.55856	gi|1594893	acetohydroxy ac	2	97	DINVVLVAPK		
N	Unus	Total	%Cov	%Cov(95)	Accessions	Names	Cor	Conf	Sequence	Modificatic	Cleavages
8	10	10	27.67296	17.61006	gi|1594899	enolase [Chlamy	2	99	ALGQLTPPEIVK		
8	10	10	27.67296	17.61006	gi|1594899	enolase [Chlamy	2	99	AVENINAIIAPALK		
8	10	10	27.67296	17.61006	gi|1594899	enolase [Chlamy	2	99	LTTENICQV	Carbamidomethyl(C)@	
8	10	10	27.67296	17.61006	gi|1594899	enolase [Chlamy	2	99	SGETEDSFIADLAVGLASGQIK		
8	10	10	27.67296	17.61006	gi|1594899	enolase [Chlamy	2	99	VNQIGTITESIEAVR		
N	Unus	Total	%Cov	%Cov(95)	Accessions	Names	Cor	Conf	Sequence	Modificatic	Cleavages
9	8.2	8.2	30.39049	12.39389	gi|1594770	fumarate hydrat	2	99	IEVEDFPAFIVVDDKGN		missed K-G
9	8.2	8.2	30.39049	12.39389	gi|1594770	fumarate hydrat	2	99	ITGEGVFLEALER		
9	8.2	8.2	30.39049	12.39389	gi|1594770	fumarate hydrat	2	99	VLQVAPEA	Oxidation(M)@17	
9	8.2	8.2	30.39049	12.39389	gi|1594770	fumarate hydrat	2	99	VNLNRPMQEVLAQLSSFPIR		
10	8.1	8.1	26.15063	13.17992	gi|1594798	UDP-glucose del	2	99	AWNSDKLPIYEPGLLEV		missed K-L
10	8.1	8.1	26.15063	13.17992	gi|1594798	UDP-glucose del	2	99	EIGFIVYALGKPLDQFLQK		
10	8.1	8.1	26.15063	13.17992	gi|1594798	UDP-glucose del	2	99	TLAEVYAHWIPR		
N	Unus	Total	%Cov	%Cov(95)	Accessions	Names	Cor	Conf	Sequence	Modificatic	Cleavages
11	8	8	15.01014	11.56187	gi|1594773	catalase/peroxic	2	99	APGVQTPVIVR		
11	8	8	15.01014	11.56187	gi|1594773	catalase/peroxic	2	99	GFFEVTHDISALTAADFLR		
11	8	8	15.01014	11.56187	gi|1594773	catalase/peroxic	2	99	GPILLEDYHLVEK		
11	8	8	15.01014	11.56187	gi|1594773	catalase/peroxic	2	99	LGPNYLLLPVNAPR		
N	Unus	Total	%Cov	%Cov(95)	Accessions	Names	Cor	Conf	Sequence	Modificatic	Cleavages
12	7.3	7.3	10.88435	5.442177	gi|4117904	photosystem I P	2	99	ALYGFDFLLSSK		
12	7.3	7.3	10.88435	5.442177	gi|4117904	photosystem I P	2	99	QILIEPVFAQWIQAAHGK		

12	7.3	7.3	10.88435	5.442177	gi|4117904	photosystem I P	2	99	TPLANLVYWK		
N	Unus	Total	%Cov	%Cov(95)	Accessions	Names	Coı	Conf	Sequence	Modificatic	Cleavages
13	7.1	7.1	26.83983	23.37662	gi|136429	Trypsin precursc	2	99	IQVRLGEHI	Oxidation(I	missed R-L
13	7.1	7.1	26.83983	23.37662	gi|136429	Trypsin precursc	2	99	LGEHNIDVI	Deamidate	missed K-I(
13	7.1	7.1	26.83983	23.37662	gi|136429	Trypsin precursc	2	99	LGEHNIDVI	Formyl(K)@	missed K-I(
N	Unus	Total	%Cov	%Cov(95)	Accessions	Names	Coı	Conf	Sequence	Modificatic	Cleavages
14	6.9	6.9	29.60993	9.929078	gi|1594652	phytoene desatı	2	99	IQFAIGLLPAIIFGQK		
14	6.9	6.9	29.60993	9.929078	gi|1594652	phytoene desatı	2	99	SDEDIIAATMTELER		
14	6.9	6.9	29.60993	9.929078	gi|1594652	phytoene desatı	1	96	LAAEQIVNDYNYK		
14	6.9	6.9	29.60993	9.929078	gi|1594652	phytoene desatı	1	95	KLTTVDHLLFSR		missed K-L
N	Unus	Total	%Cov	%Cov(95)	Accessions	Names	Coı	Conf	Sequence	Modificatic	Cleavages
15	6.2	6.2	14.24767	4.660453	gi|4117907	photosystem I P	2	99	AIMADLYPSFAK		
15	6.2	6.2	14.24767	4.660453	gi|4117907	photosystem I P	2	99	IAVDRNPVETSFEK		missed R-N
15	6.2	6.2	14.24767	4.660453	gi|4117907	photosystem I P	2	99	LLDAGVDPK		
N	Unus	Total	%Cov	%Cov(95)	Accessions	Names	Coı	Conf	Sequence	Modificatic	Cleavages
16	6.1	6.1	31.28492	7.821229	gi|1594873	flagellar associat	2	99	ALYGLTDAAALTDLK		
16	6.1	6.1	31.28492	7.821229	gi|1594873	flagellar associat	2	99	NSAVSPLVAWIYYQPK		
16	6.1	6.1	31.28492	7.821229	gi|1594873	flagellar associat	2	99	VTGVNVFPTTR		
N	Unus	Total	%Cov	%Cov(95)	Accessions	Names	Coı	Conf	Sequence	Modificatic	Cleavages
17	5.2	5.2	31.94444	6.746032	gi|1594876	vacuolar ATP syı	2	99	IPLFSAAGLI	Carbamidomethyl(C)@	
17	5.2	5.2	31.94444	6.746032	gi|1594876	vacuolar ATP syı	2	99	TIFNSLDLAWSLLR		
N	Unus	Total	%Cov	%Cov(95)	Accessions	Names	Coı	Conf	Sequence	Modificatic	Cleavages
18	4	4	29.23077	29.23077	gi|1594897	predicted protei	2	99	AKPLVEQLIAITSGTDAGAK		
18	4	4	29.23077	29.23077	gi|1594897	predicted protei	2	99	ASAAQKEEIAALVTELSR		
N	Unus	Total	%Cov	%Cov(95)	Accessions	Names	Coı	Conf	Sequence	Modificatic	Cleavages
19	3.4	3.4	26.42857	6.904762	gi|1594628	calreticulin 2, ca	2	99	SGSIFDNILVTDDLEAAK		missed K-K
19	3.4	3.4	26.42857	6.904762	gi|1594628	calreticulin 2, ca	1	95	FVGFELWQVK		
N	Unus	Total	%Cov	%Cov(95)	Accessions	Names	Coı	Conf	Sequence	Modificatic	Cleavages
21	3	3	22.61905	2.97619	gi|1594706	ADP-glucose pyı	2	99	AIEDVLILSGDHLYR		
27	2	2	17.89883	3.11284	gi|1594673	ADP-glucose pyı	2	99	EGVEDFLILSGDHLYR		
N	Unus	Total	%Cov	%Cov(95)	Accessions	Names	Coı	Conf	Sequence	Modificatic	Cleavages
28	2	2	19.18919	3.783784	gi|1594716	predicted protei	2	99	TIGGVVITVARPAK		
29	1.3	1.3	7.142857	2.813853	gi|1594697	predicted protei	1	95	TGALVGGVLGAPR		

dMass	Prec MW	Prec m/z	Theor MW	Theor m/z	Theor z	Sc	Spectrum	Time
-1.01038	1128.578	565.2961	1129.588	565.8013	2	13	1.1.1.810.5	29.1462
0.005322	1971.995	658.3391	1971.99	658.3373	3	21	1.1.1.965.3	54.9247
-1.95831	2098.127	700.3829	2100.085	701.0357	3	18	1.1.1.925.4	52.1874
-0.00798	1339.644	670.8293	1339.652	670.8333	2	14	1.1.1.813.2	29.6882
0.001191	1138.688	570.351	1138.686	570.3504	2	14	1.1.1.882.3	45.0549
0.016534	1651.871	551.6309	1651.854	551.6254	3	16	1.1.1.912.2	49.9313
0.011012	2827.358	943.4598	2827.347	943.4562	3	22	1.1.1.893.2	47.5633
-0.00079	1667.849	556.9568	1667.849	556.9571	3	15	1.1.1.885.2	45.702

dMass	Prec MW	Prec m/z	Theor MW	Theor m/z	Theor z	Sc	Spectrum	Time
0.006084	1752.977	585.3329	1752.971	585.3309	3	17	1.1.1.1071.	64.1166
0.007084	1209.61	605.8123	1209.603	605.8088	2	14	1.1.1.815.2	30.152
0.016563	1717.815	573.6122	1717.798	573.6067	3	17	1.1.1.803.2	27.5224
-0.04417	3053.5	1018.841	3053.544	1018.855	3	16	1.1.1.1583.	79.0657
0.003188	2361.146	788.0558	2361.142	788.0547	3	21	1.1.1.860.5	40.4526
0.013134	1909.085	478.2786	1909.072	478.2753	4	12	1.1.1.1031.	60.8319
-0.00888	1066.63	534.3221	1066.639	534.3266	2	13	1.1.1.828.2	33.1177

dMass	Prec MW	Prec m/z	Theor MW	Theor m/z	Theor z	Sc	Spectrum	Time
-0.01113	1264.728	633.3713	1264.739	633.3768	2	14	1.1.1.835.3	34.7432
0.006845	1435.847	718.9306	1435.84	718.9272	2	17	1.1.1.866.4	41.796
0.019407	2369.245	790.7557	2369.226	790.7493	3	17	1.1.1.913.2	50.1128
0.016882	2207.113	736.7115	2207.096	736.7059	3	19	1.1.1.1031.	60.8833
-0.01565	1628.858	815.4361	1628.873	815.444	2	17	1.1.1.866.5	41.8473

dMass	Prec MW	Prec m/z	Theor MW	Theor m/z	Theor z	Sc	Spectrum	Time
0.001025	2571.254	858.092	2571.253	858.0917	3	17	1.1.1.962.3	54.3809
-0.00341	1432.753	717.3837	1432.756	717.3854	2	16	1.1.1.892.3	47.382
0.019822	1913.049	638.6903	1913.029	638.6837	3	16	1.1.1.936.2	52.6424
0.01127	2311.258	771.4267	2311.247	771.423	3	21	1.1.1.1008.	58.3069
0.026741	2526.339	843.1201	2526.312	843.1112	3	22	1.1.1.925.3	52.136
0.014666	2178.224	727.0818	2178.209	727.0769	3	16	1.1.1.1027.	60.4155
0.010654	1454.778	485.9332	1454.767	485.9296	3	14	1.1.1.870.4	42.6236

dMass	Prec MW	Prec m/z	Theor MW	Theor m/z	Theor z	Sc	Spectrum	Time
-0.00274	1135.669	568.8416	1135.671	568.8429	2	15	1.1.1.794.3	25.6912
0.010861	2109.064	704.0286	2109.053	704.025	3	20	1.1.1.997.2	57.4422
0.003287	1524.822	509.2813	1524.819	509.2802	3	16	1.1.1.852.5	38.6953
0.016639	1535.899	768.9568	1535.882	768.9485	2	14	1.1.1.876.4	43.8133

dMass	Prec MW	Prec m/z	Theor MW	Theor m/z	Theor z	Sc	Spectrum	Time
-0.00839	1359.699	680.8568	1359.707	680.861	2	16	1.1.1.923.2	51.8757
-0.02172	2048.099	683.707	2048.121	683.7142	3	20	1.1.1.978.2	55.8439

-0.02287	1203.642	602.8284	1203.665	602.8399	2	14	1.1.1.872.5	43.14

dMass	Prec MW	Prec m/z	Theor MW	Theor m/z	Theor z	Sc	Spectrum	Time
0.15751	6061.254	1011.216	6061.097	1011.19	6	17	1.1.1.1014.	59.0861
0.118557	4517.373	904.4818	4517.254	904.458	5	17	1.1.1.997.4	57.545
0.154034	5558.917	927.4934	5558.763	927.4677	6	23	1.1.1.1016.	59.3974

dMass	Prec MW	Prec m/z	Theor MW	Theor m/z	Theor z	Sc	Spectrum	Time
-0.01861	1728.015	865.0149	1728.034	865.0242	2	15	1.1.1.1162.	66.8431
0.012552	1692.8	847.4073	1692.788	847.4011	2	15	1.1.1.924.3	52.0057
-0.00647	1539.75	770.8825	1539.757	770.8857	2	13	1.1.1.827.4	32.988
-0.03999	1428.769	477.2636	1428.809	477.2769	3	12	1.1.1.827.5	33.0394

dMass	Prec MW	Prec m/z	Theor MW	Theor m/z	Theor z	Sc	Spectrum	Time
-0.00833	1325.661	663.8376	1325.669	663.8418	2	16	1.1.1.864.4	41.3308
0.009778	1603.83	535.6174	1603.821	535.6141	3	18	1.1.1.798.5	26.7223
-0.01234	926.495	464.2548	926.5073	464.2609	2	14	1.1.1.779.5	22.3129

dMass	Prec MW	Prec m/z	Theor MW	Theor m/z	Theor z	Sc	Spectrum	Time
-0.00171	1534.823	768.4186	1534.824	768.4194	2	19	1.1.1.883.4	45.3392
-0.00472	1834.957	612.6596	1834.962	612.6612	3	15	1.1.1.959.2	54.042
-0.00534	1189.64	595.8274	1189.646	595.83	2	14	1.1.1.806.2	28.1669

dMass	Prec MW	Prec m/z	Theor MW	Theor m/z	Theor z	Sc	Spectrum	Time
-0.00849	2163.117	722.0464	2163.126	722.0492	3	18	1.1.1.880.4	44.6411
0.005996	1647.904	824.9595	1647.898	824.9565	2	16	1.1.1.1084.	64.5669

dMass	Prec MW	Prec m/z	Theor MW	Theor m/z	Theor z	Sc	Spectrum	Time
-0.00241	1982.102	661.7081	1982.105	661.7089	3	17	1.1.1.908.2	49.3594
0.014054	1886.025	629.6823	1886.011	629.6776	3	19	1.1.1.888.5	46.5543

dMass	Prec MW	Prec m/z	Theor MW	Theor m/z	Theor z	Sc	Spectrum	Time
-0.04113	2035.006	679.3427	2035.047	679.3564	3	14	1.1.1.890.5	47.0194
0.003059	1251.668	626.8414	1251.665	626.8399	2	12	1.1.1.905.4	48.8667

dMass	Prec MW	Prec m/z	Theor MW	Theor m/z	Theor z	Sc	Spectrum	Time
-0.00804	1712.902	571.9745	1712.91	571.9772	3	17	1.1.1.877.2	43.9431
0.021811	1861.943	621.6549	1861.921	621.6476	3	15	1.1.1.910.4	49.6712

dMass	Prec MW	Prec m/z	Theor MW	Theor m/z	Theor z	Sc	Spectrum	Time
-0.00531	1380.84	461.2873	1380.845	461.289	3	14	1.1.1.807.2	28.2962
-0.00849	1166.669	584.3416	1166.677	584.3459	2	12	1.1.1.816.4	30.4867
