# Supplementary material for: Metabolic labeling with stable isotope nitrogen (15N) to follow amino acid and protein turnover of three plastid proteins in Chlamydomonas reinhardtii
Source: Proteome Sci. 2014 Mar 3;12:14. doi: 10.1186/1477-5956-12-14 (PMC3943399; doi:10.1186/1477-5956-12-14)
Supplement: Additional file 3: Table S1 — Identification of unique peptides and percent coverage for accepted proteins. [file 1477-5956-12-14-S3.pdf]

Additional Table 1. Identification of unique peptides and percent coverage for accepted proteins. Same colored blocks identify the overlapping sections. Only one version of the peptide including any extensions was considered unique and placed in the “Unique” column.

ATP synthase CF1 α subunit (NP_958406.1) (total length = 508 aa; total aa from unique list = 219; protein sequence coverage = 43 %

**Overlapping**	**Unique**
AIESPAPGIVAR	AIESPAPGIVAR
ASSVAQVLNTLK	ASSVAQVLNTLK
IAEIPVGEAYLGR	IAEIPVGEAYLGR
MVDFGIVFQVGDGIAR	LKLELAQFAELEAFSQFASDLDQATQNQLAR
STLTFTPEAEGLVK	MVDFGIVFQVGDGIAR
TAIAVDTILNQK	QAINEYLEEFKSQAK
VVDGLARPVDGK	LREILKQPQSSPLSVEEQVASLYAGTNGYLDKLEVSQVR
SYLANSYPK	RSVYEPLATGLVAVDAMIPVGR
LELAQFAELEAFSQFASDLDQATQNQLAR	STLTFTPEAEGLVK
LKLELAQFAELEAFSQFASDLDQATQNQLAR	TAIAVDTILNQK
QAINEYLEEFK	TPEELSNLIKDLIEQYTPEVK
QAINEYLEEFKSQAK	VVDGLARPVDGK
LREILKQPQSSPLSVEEQVASLYAGTNGYLDKLEVSQVR	
EILKQPQSSPLSVEEQVASLYAGTNGYLDKLEVSQVR	
QPQSSPLSVEEQVASLYAGTNGYLDKLEVSQVR	
RSVYEPLATGLVAVDAMIPVGR	
SVYEPLATGLVAVDAMIPVGR	
DLIEQYTPEVK	
TPEELSNLIK	
TPEELSNLIKDLIEQYTPEVK	

ATP synthase CF1 β subunit (NP_958414) (total length 491 aa; total aa from unique list = 199); protein sequence coverage = 40.5 %

**Overlapping**	**Unique**
AHGGVSVFAGVGER	AHGGVSVFAGVGER
QDVLFFIDNIFR	DVNKQDVLFFIDNIFR
DVNKQDVLFFIDNIFR	FLSQPFFVAEVFTGSPGKYVSLAETIEGFGK
FLSQPFFVAEVFTGSPGKYVSLAETIEGFGK	FVQAGAEVSALLGR
FVQAGAEVSALLGR	GMEVVDTGKPLSVPVGK
GMEVVDTGKPLSVPVGK	GQVPNIYNALTIR
GQVPNIYNALTIR	IFNVLGEPVDNMGNVK
IFNVLGEPVDNMGNVK	IVQIIGPVLDIVFAK
IVQIIGPVLDIVFAK	TAPAFVDLDTR
TAPAFVDLDTR	TVLIMELINNIAK
TVLIMELINNIAK	VALTALTMAEYFR
VALTALTMAEYFR	YKELQDIIAILGLDELSEEDRLIVAR
YKELQDIIAILGLDELSEEDR	
YKELQDIIAILGLDELSEEDRLIVAR	
ELQDIIAILGLDELSEEDRLIVAR	
IGLFGGAGVGK	

Ribulose-1,5-bisphosphate carboxylase/oxygenase large subunit (RuBisCo- ABA42220) (total length = 475 aa; total aa from unique list = 142); protein sequence coverage = 29.9 %

**Overlapping**	**Unique**
DDENVNSQPFMR	DRFLFVAEAIYK
DRFLFVAEAIYK	ELGVPIIMHDYLTGGFTANTSLAIYCR
ELGVPIIMHDYLTGGFTANTSLAIYCR	EVTLGFVDLMRDDYVEKDR
EVTLGFVDLMR	GGLDFTKDDENVNSQPFMR
EVTLGFVDLMRDDYVEK	GLLGCTIKPK
EVTLGFVDLMRDDYVEKDR	LTYYTPDYVVR
FLFVAEAIYK	TFVGPPHGIQVER
GGLDFTKDDENVNSQPFMR	WSPELAAACEVWK
GLLGCTIKPK	DTDILAAFR
LTYYTPDYVVR	FEFDTIDKL
TFVGPPHGIQVER	
WSPELAAACEVWK	
DTDILAAFR	
FEFDTIDKL	

Mitochondrial F1F0 ATP synthase, α subunit (EDP07337) (total length = 569 aa; total aa from unique list =170); protein sequence coverage = 29.9 %

**Overlapping**	**Unique**
AVDALVPIGR	AVDALVPIGR
EVAAFAQFGSDLDAATQYVLER	EVAAFAQFGSDLDAATQYVLER
GMALNLQADHVGVVVFGNDSLIHQGDLVYR	GMALNLQADHVGVVVFGNDSLIHQGDLVYR
GYLDKVPVNQITACEDVILK	GYLDKVPVNQITACEDVILK
TAVALDCILHQNYLNGLTNKK	TAVALDCILHQNYLNGLTNKK
TGQIVNVPVGPGTLGR	TGQIVNVPVGPGTLGR
VGSAAQFPGMK	VGSAAQFPGMK
VLSVGDGIAR	VLSVGDGIAR
VVDALGQPIDGK	VVDALGQPIDGKGPLTNVR
VVDALGQPIDGKGPLTNVR	HAVIIYDDLSK
HAVIIYDDLSK	

S-Adenosyl homocysteine hydrolase (EDP03365.1) (total length = 483 aa; total aa from non-overlapping peptides = 144); protein sequence coverage = 29.8 %

**Unique**
ALSVDHVNGR
DGTLPNPDSTDNAEFK
FIFPDGHGVIVLAEGR
ITGSLHMTIQTAVLIETLTALGAEVR
LSADQAAYINVPVDGPYKPAHYR
NNAIVGNIGHFDNEVDMAGLYAWPGIK
TAFIAGYGDVGK
VMGVSEETTTGVKR
