# Supplementary material for: Metabolic labeling with stable isotope nitrogen (15N) to follow amino acid and protein turnover of three plastid proteins in Chlamydomonas reinhardtii
Source: Proteome Sci. 2014 Mar 3;12:14. doi: 10.1186/1477-5956-12-14 (PMC3943399; doi:10.1186/1477-5956-12-14)
Supplement: Additional file 4 — List of peptides identified for each of the five proteins at various time points in the experiments. [file 1477-5956-12-14-S4.pdf]

ATP synthase CF1 alpha subunit		time (hr)	1	4	8	16	32	64	128
MS/MS		MS							
AIESPAPGIVAR		AIESPAPGIVA	0.967	0.97	0.97296	0.79241389	0.503851	0.399635	0.299685
ASSVAQVLNTLK		ASSVAQVLNT	0.998	1	0.95225	0.67658123	0.413117	0.303767	0.21067
DLIEQYTPEVK		DLIEQYTPEVK	0.988	0.98	0.97601	0.74314637	0.430988	0.379736	0.255034
IAEIPVGEAYLGR		IAEIPVGEAYL	0.969	0.97	0.96919	0.76875913	0.432598	0.305571	0.228241
QAINEYLEEFK		QAINEYLEEFK	0.991	0.99	0.95295	0.71401771	0.435499	0.302573	0.214098
SVYEPLATGLVAVDAMIPVGR		SVYEPLATGLV	0.984	0.99	0.95362	0.75454028	0.551497	0.387145	0.324001
TPEELSNLIK		TPEELSNLIK	0.98	0.99	0.96654	0.73776269	0.430602	0.344858	0.255482
MVDFGIVFQVGDGIAR		MVDFGIVFQVGDGIA		0.99	0.93255	0.70628158	0.55379	0.449113	0.407564
STLTFTPEAEGLVK		STLTFTPEAEGLVK			0.96408	0.68270078	0.503866	0.293686	0.253154
SYLANSYPK		SYLANSYPK		0.89		0.6673004	0.388549	0.35827	0.203731
TPEELSNLIKDLIEQYTPEVK		TPEELSNLIKDLIEQYTPEVK			0.95909	0.73409205	0.452853	0.340571	0.247233
VVDGLARPVDGK									
LELAQFAELEAFSQFASDLDQATQNQLAR									
LKLELAQFAELEAFSQFASDLDQATQNQLAR									
LREILKQPQSSPLSVEEQVASLYAGTNGYLDKLEVSQVR									
QAINEYLEEFKSQAK									
QPQSSPLSVEEQVASLYAGTNGYLDKLEVSQVR									
RSVYEPLATGLVAVDAMIPVGR									
TAIAVDTILNQK									
EILKQPQSSPLSVEEQVASLYAGTNGYLDKLEVSQVR									
(pink)		peptides with > 95 % confidence and used in the degradation studies.							
(purple)		peptides with >95% confidence level but did not appear **at all time** points							
MS/MS peptides missing from MS dataset		peptides with >95 % confidence; did not appear at any time point .							

ATP synthase CF1 beta subunit		time (hr)	1	4	8	16	32	64	128	
MS/MS		MS								
AHGGVSVFAGVGER		AHGGVSVFAGVGER	0.94	0.97	0.96	0.75	0.48	0.42	0.37	
DVNKQDVLFFIDNIFR		DVNKQDVLFFIDNIFR	0.99	0.99	0.96	0.81	0.48	0.38		
FVQAGAEVSALLGR		FVQAGAEVSALLGR	0.98	0.98	0.93	0.75	0.45	0.42	0.46	
GMEVVDTGKPLSVPVGK		GMEVVDTGKPLSVPVGK	0.95	0.97	0.96	0.74	0.41	0.34	0.3	
GQVPNIYNALTIR		GQVPNIYNALTIR	0.96	0.96	0.98	0.72	0.44	0.35	0.3	
TAPAFVDLDTR		TAPAFVDLDTR	0.99	0.98	0.92	0.72	0.46	0.38	0.33	
TVLIMELINNIAK		TVLIMELINNIAK	0.99	0.99	0.98	0.75	0.44	0.37	0.32	
VALTALTMAEYFR		VALTALTMAEYFR	0.97	0.97	0.92	0.69	0.4	0.43	0.28	
ELQDIIAILGLDELSEEDRLIVAR		ELQDIIAILGLDELSEEDRLIVAR		0.97	0.98	0.85	0.56	0.5	0.33	
FLSQPFFVAEVFTGSPGKYVSLAETIEGFGK		FLSQPFFVAEVFTGSPGKYVSLAETIE[illegible]		0.99	0.98	0.83	0.58	0.5		
IVQIIGPVLDIVFAK		IVQIIGPVLDIVFAK		1	0.96	0.69	0.4	0.34		
QDVLFFIDNIFR		QDVLFFIDNIFR		0.97	0.97	0.8	0.46	0.4	0.47	
IFNVLGEPVDNMGNVK										
YKELQDIIAILGLDELSEEDR										
YKELQDIIAILGLDELSEEDRLIVAR										
IGLFGGAGVGK										
		peptides with > 95 % confidence and used in the degradation studies.								
		peptides with >95% confidence level but did not appear at all time points								
MS/MS peptides missing from MS dataset		peptides with >95 % confidence; did not appear at any time point .								

Ribulose-1,5-bisphosphate carbo	time (hr)	1	4	8	16	32	64	128
DDENVNSQPFMR	DDENVNSQPFMR	0.99	0.92	0.91	0.87	0.57	0.38	0.264032
DTDILAAFR	DTDILAAFR	0.96	0.96	0.959	0.92	0.602	0.43	0.294327
EVTLGFVDLMRDDYVEK	EVTLGFVDLMRDDYVEK	0.99	0.98	0.982	0.86	0.572		0.473163
FLFVAEAIYK	FLFVAEAIYK	0.99	0.99	0.987	0.85	0.555	0.36	0.2511
GGLDFTKDDENVNSQPFMR	GGLDFTKDDENVNSQPFMR	0.99	0.94	0.901	0.84	0.623	0.38	0.293118
GLLGCTIKPK	GLLGCTIKPK	1	1	0.917	0.81	0.575	0.38	0.254072
	LGCTIKPK	0.95	0.99	0.902	0.79	0.549	0.33	0.213115
LTYYTPDYVVR	LTYYTPDYVVR	0.98	0.98	0.97	0.91	0.521	0.33	
TFVGPPHGIQVER	TFVGPPHGIQVER	0.96	0.97	0.965	0.86	0.568	0.4	0.292152
WSPELAAACEVWK	WSPELAAACEVWK	1	0.99	0.999	0.86	0.535	0.35	0.32444
EVTLGFVDLMR	EVTLGFVDLMR		0.98	0.97	0.91	0.699	0.43	0.316465
DRFLFVAEAIYK	**MS dataset**							
FEFDTIDKL								
ELGVPIIMHDYLTGGFTANTSLAIYCR								
EVTLGFVDLMRDDYVEKDR								
	peptides with > 95 % confidence and used in the degradation studies.							
	peptides with >95% confidence level but did not appear **<u>at all time</u>** points							
MS/MS peptides missing from M	peptides with >95 % confidence; did not appear at any time point .							
