# Supplementary material for: Metabolic labeling with stable isotope nitrogen (15N) to follow amino acid and protein turnover of three plastid proteins in Chlamydomonas reinhardtii
Source: Proteome Sci. 2014 Mar 3;12:14. doi: 10.1186/1477-5956-12-14 (PMC3943399; doi:10.1186/1477-5956-12-14)
Supplement: Additional file 5 — List of the peptides for the three plastid proteins. [file 1477-5956-12-14-S5.pdf]

ATP Synthase CF1 α	$R_{t} = {}^{15}N / ({}^{15}N + {}^{14}N\}$						
**time (hr)**	**1**	**4**	**8**	**16**	**32**	**64**	**128**
**AIESPAPGIVAR**	0.966663	0.972502	0.972959	0.792414	0.503851	0.399635	0.299685
**ASSVAQVLNTLK**	0.997772	0.995054	0.952251	0.676581	0.413117	0.303767	0.21067
**DLIEQYTPEVK**	0.987638	0.976084	0.976013	0.743146	0.430988	0.379736	0.255034
**IAEIPVGEAYLGR**	0.969459	0.970181	0.969187	0.768759	0.432598	0.305571	0.228241
**QAINEYLEEFK**	0.991361	0.992658	0.952955	0.714018	0.435499	0.302573	0.214098
**SVYEPLATGLVAVDAMIPVGR**	0.983748	0.987991	0.953621	0.75454	0.551497	0.387145	0.324001
**TPEELSNLIK**	0.980119	0.988945	0.966543	0.737763	0.430602	0.344858	0.255482

ATP Synthase CF1 β	$R_{t\,=}\ ^{15}N/\ (^{15}N+\ ^{14}N\}$						
**time (hr)**	**1**	**4**	**8**	**16**	**32**	**64**	**128**
**AHGGVSVFAGVGER**	0.938843	0.967584	0.964389	0.749406	0.483626	0.420408	0.367815
**DVNKQDVLFFIDNIFR**	0.989786	0.991313	0.964217	0.814891	0.484986	0.378057	
**FVQAGAEVSALLGR**	0.975997	0.978602	0.931793	0.746925	0.449177	0.416002	0.455801
**GMEVVDTGKPLSVPVGK**	0.946142	0.968896	0.957486	0.73594	0.411922	0.338031	0.29708
**GQVPNIYNALTIR**	0.958091	0.962212	0.975895	0.720643	0.436667	0.345791	0.304967
**TAPAFVDLDTR**	0.986083	0.981989	0.915119	0.715164	0.461506	0.376451	0.328967
**TVLIMELINNIAK**	0.993206	0.991957	0.977695	0.750675	0.437887	0.372755	0.315051
**VALTALTMAEYFR**	0.965646	0.974121	0.923083	0.687823	0.397342	0.4283	0.277961

Rubisco	$R_{t\,=}\ {}^{15}N/\ ({}^{15}N+\ {}^{14}N\}$						
**time (hr)**	**1**	**4**	**8**	**16**	**32**	**64**	**128**
**DDENVNSQPFMR**	0.990366	0.919348	0.91046	0.871574	0.569506	0.377464	0.264032
**DTDILAAFR**	0.96007	0.957836	0.95913	0.919012	0.601616	0.425887	0.294327
**EVTLGFVDLMRDDYVEK**	0.9851	0.984065	0.981522	0.860627	0.572392		0.473163
**FLFVAEAIYK**	0.985988	0.991389	0.986901	0.850569	0.555156	0.363867	0.2511
**GGLDFTKDDENVNSQPFMR**	0.986761	0.941911	0.901304	0.839144	0.623325	0.37981	0.293118
**GLLGCTIKPK**	1	0.999	0.916656	0.808232	0.574678	0.380129	0.254072
**LGCTIKPK**	0.952653	0.991117	0.901868	0.79185	0.548577	0.327486	0.213115
**LTYYTPDYVVR**	0.981076	0.981002	0.970039	0.905598	0.52072	0.333977	
**TFVGPPHGIQVER**	0.961156	0.973465	0.965399	0.864789	0.568267	0.396561	0.292152
**WSPELAAACEVWK**	1	0.993201	0.999	0.86064	0.535257	0.352601	0.32444
